# HOXD10 attenuates renal fibrosis by inhibiting NOX4-induced ferroptosis

**DOI:** 10.1038/s41419-024-06780-w

**Published:** 2024-06-06

**Authors:** Xin Li, Tian-Kui Ma, Pu Wang, Hang Shi, Sang Hai, Yu Qin, Yun Zou, Wan-Ting Zhu, Hui-Min Li, Yan-Nong Li, Li Yin, Yan-Yan Xu, Qi Yang, Shuang Zhang, Hong Ding

**Affiliations:** 1 Nephrology Department, Fourth Hospital of China Medical University, Shenyang, China; 2 Biological Therapy Department, First Hospital of China Medical University, Shenyang, China; 3 General Practice Department, Fourth Hospital of China Medical University, Shenyang, China; 4 Intensive Care Unit Department, Sun Yat-sen Memorial Hospital, Guangzhou, China

**Keywords:** Cell death, Chronic kidney disease

## Abstract

In chronic kidney disease (CKD), renal fibrosis is an unavoidable result of various manifestations. However, its pathogenesis is not yet fully understood. Here, we revealed the novel role of Homeobox D10 (HOXD10) in CKD-related fibrosis. HOXD10 expression was downregulated in CKD-related in vitro and in vivo fibrosis models. UUO model mice were administered adeno-associated virus (AAV) containing HOXD10, and HOXD10 overexpression plasmids were introduced into human proximal tubular epithelial cells induced by TGF-β1. The levels of iron, reactive oxygen species (ROS), lipid ROS, the oxidized glutathione/total glutathione (GSSG/GSH) ratio, malonaldehyde (MDA), and superoxide dismutase (SOD) were determined using respective assay kits. Treatment with AAV–HOXD10 significantly attenuated fibrosis and renal dysfunction in UUO model mice by inhibiting NOX4 transcription, ferroptosis pathway activation, and oxidative stress. High levels of NOX4 transcription, ferroptosis pathway activation and profibrotic gene expression induced by TGF-β1/erastin (a ferroptosis agonist) were abrogated by HOXD10 overexpression in HK-2 cells. Moreover, bisulfite sequencing PCR result determined that HOXD10 showed a hypermethylated level in TGF-β1-treated HK-2 cells. The binding of HOXD10 to the NOX4 promoter was confirmed by chromatin immunoprecipitation (ChIP) analysis and dual-luciferase reporter assays. Targeting HOXD10 may represent an innovative therapeutic strategy for fibrosis treatment in CKD.

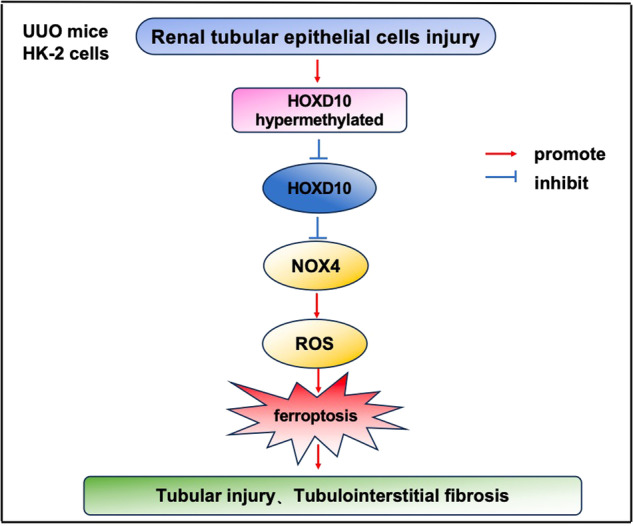

## Introduction

Chronic kidney disease (CKD), which mounting evidence suggests that the incidence is on the rise, posing a significant threat to human health [1]. Renal fibrosis occurs in almost all CKD patients and contributes to end-stage renal disease [2, 3]. Presently, a variety of treatment strategies for renal fibrosis have been rigorously assessed in mouse models. These include: (1) linagliptin, an inhibitor for dipeptidyl peptidase-4 [4], (2) empagliflozin, an inhibitor for sodium-dependent glucose transporter 2 [5, 6], (3) Sirtuin3 activation strategies [7], (4) glycolysis inhibitors [8], (5) mineralocorticoid antagonists [9], (6) angiotensin converting enzyme inhibitors and Ang-II receptor blockers [4], (7) AcSDKP, an endogenous antifibrotic peptide associated with phosphorylation of fibroblast growth factor receptor 1 [10], and (8) glucocorticoid receptor targeting in endothelial and podocyte cells [11, 12]. However, currently available clinical therapies only delay or prevent the progression of CKD to ESRD, the only option for treating this condition is kidney transplantation. Thus, a better understanding of renal fibrosis will help identify new biomarkers and antifibrotic treatments for CKD.

The defining feature of renal interstitial fibrosis (RIF) is the abnormal extracellular matrix (ECM) deposition. In the progression of RIF, myofibroblasts may arise from epithelial cells undergoing epithelial-to-mesenchymal transition (EMT), from endothelial cells via endothelial-to-mesenchymal transition, or from M2 macrophages originating in the bone marrow through macrophage-to-mesenchymal transition. These myofibroblasts are closely associated with excessive ECM production in the tubular interstitium [13, 14]. Additionally, studies have shown that signaling pathway including transforming growth factor-β (TGF-β), Wnt, Hedgehog and Notch are pivotal in triggering the mesenchymal transition processes [15]. Moreover, studies have revealed an association between RIF and ferroptosis. The characteristic of ferroptosis is iron-dependent lipid peroxides accumulation, which is a novel regulated cell death pattern that promotes the development of fibrotic diseases, such as idiopathic pulmonary fibrosis, liver fibrosis and cardiomyopathy [16–18]. Previous studies have suggested that under the condition of ischemia/reperfusion-induced acute kidney injury (AKI), ferroptosis plays a vital role and that NADPH oxidase 4 (NOX4), which is enriched in kidney and is responsible for reactive oxygen species (ROS) generation, functioning as a key ferroptosis regulator by increasing ferroptosis-dependent cytotoxicity via oxidative stress-induced lipid peroxidation, thus contributing to oxidative stress injury, ECM accumulation, EMT and renal fibrosis [19, 20]. Although AKI and CKD are interconnected, experimental research about ferroptosis and renal fibrosis in CKD patients is lacking.

Homeobox D10 (HOXD10), which is a tumor suppressor, is aberrantly hypermethylated and expressed at low levels in many tumor cells, such as colon adenocarcinoma cells, clear cell renal cell carcinoma cells and hepatocellular carcinoma cells, and it participates in multiple cellular processes, such as apoptosis, proliferation, migration, invasion and EMT [21–23]. HOXD10 has been confirmed to be crucial for nephrogenesis and renal development, and HOXD10 gene dysfunction leads to ureteric bud induction loss, branching reducing and nephrogenesis decreasing [24, 25]. Interestingly, the development of pulmonary fibrosis is closely associated with the hypermethylation of HOXD10 [26]. Nevertheless, the relationship between HOXD10 expression and renal fibrosis in individuals with CKD is currently unknown.

A recent study indicated that in unilateral ureteral obstruction (UUO) mice and TGF-β1-stimulated human proximal tubular epithelial cells (HK-2 cells), tectorigenin may ameliorate renal fibrosis and HK-2 cell injury by inhibiting ferroptosis by regulating ferroptosis biomarker expression, as shown by alterations in glutathione peroxidase 4 (GPX4), 4-hydroxy-2-nonenal (4-HNE) level, cystine/glutamate antiporter solute carrier family 7 member 11 (SLC7A11), alongside the modulation of NOX4 expression [27]. We predicted the existence of an HOXD10 binding site within the NOX4 promoter region. To date, the mechanism on HOXD10 regulates NOX4 is unclear. We were interested in exploring whether HOXD10 could regulate renal fibrosis via NOX4-mediated ferroptosis in CKD patients.

In our research, we investigate the hypothesis that HOXD10 acts on the NOX4 promoter to reduce its transcriptional activity, thus providing protection from renal fibrosis and injury by affecting NOX4-dependent ferroptosis in both UUO-induced in vivo models and TGF-β1-treated HK-2 cell models. We further examined if the HOXD10 gene exhibits hypermethylation and reduced expression in TGF-β1-stimulated HK-2 cells. This study introduces the initial evidence suggesting that HOXD10 may serve as a new target for renal fibrosis treatment.

## Methods

### Clinical renal biopsy samples

The Ethics Committee of the Fourth Affiliated Hospital of China Medical University approved all the experimental procedures (Number: EC-2023-KS-027) and followed the tenets of the Declaration of Helsinki of 1975, as revised in 1983. CKD is diagnosed based on international guidelines (estimated glomerular filtration rate (eGFR) <60 ml/min per 1.73 m^2^ is required), the presence of a kidney damage marker, or both persisting for at least 3 months. The renal tissue sections that were studied were the residual portions of diagnostic renal biopsies from CKD patients (*n* = 20). Normal kidney tissues were obtained from nephrectomies that were performed on tumor patients (*n* = 20). The patients’ biochemical data were retrieved from the hospital management system. All the recruited subjects were informed of the use of their renal biopsy samples and signed the informed consents.

### Cell culture

From the American Type Culture Collection (ATCC, Manassas, VA, USA), we purchased HK-2 cells and cultured the cells with medium containing DMEM/F12 (1:1; Grand Island, NY, USA) added with FBS (Grand Island, NY, USA, 10%), penicillin (Grand Island, NY, USA, 100 U/mL) and streptomycin (Grand Island, NY, USA, 100 µg/mL) in 5% CO_2_ at 37 °C. We divided the cells into three groups: (1) control group (Sham group); (2) the dimethyl sulfoxide (DMSO) control group (DMSO group), in which DMSO concentration <0.1% and acts as the solvent control; and (3) the TGF-β1 group (TGF-β1 group), in which recombinant human TGF-β1 protein (10 ng/mL, R&D Systems, Minneapolis, MN, USA) was administered. The cells were treated with DMSO or TGF-β1 for 48 h.

### Plasmids, 5-aza-2′-deoxycytidine (5-Aza, the demethylation drug), erastin (the ferroptosis agonist), ferrostatin-1 (the ferroptosis inhibitor) and transfection

An HOXD10 overexpression plasmid, synthesized using the pcDNA3.1 expression vector, was acquired from Sangon Biotechnology (Shanghai, China). HK-2 cells exposed to 5-Aza (Sigma-Aldrich, St. Louis, MO, USA) dissolved in DMSO at concentrations of 1, 2, or 5 µM for 48 h as a demethylation treatment. HK-2 cells were exposed to the ferroptosis agonist erastin (Selleck, Houston, TX, USA) dissolved in DMSO at a concentration of 10 μM for 48 h to induce ferroptosis. HK-2 cells exposed to the ferroptosis inhibitor ferrostatin-1 (Selleck, Houston, TX, USA) diluted in DMSO at a concentration of 1 μM for 48 h to prevent ferroptosis. The concentrations of these drugs were chosen according to previous studies [28–31].

The HOXD10 overexpression plasmid was transfected at a dose of 2000 ng. In TGF-β1 group, cells were transfected with the empty vector or HOXD10 overexpression plasmids and named TGF-β1 + Vector or TGF-β1 + HOXD10 (+) group.

### Animal experiments

We received ethical approval from the Ethics Committee of China Medical University (KT2023264) and the Institutional Animal Care and Use Committee (IACUC) of China Medical University approved all the animal experimental procedures. From the Model Animal Research Center of Nanjing University (Nanjing, China), we acquired 7-week-old C57BL/6 mice (*n* = 40, male) and raised them at the Laboratory Animal Centre of China Medical University. Food and water ad libitum were provided to the mice. Feeding environment included a 12-h:12-h light/dark cycle at 22 ± 2.0 °C with a humidity of 50% ± 20%.

We provided the adeno-associated virus (AAV) related information and transfection method in the Supplementary Methods. We divided the mice into four groups: (1) UUO + AAV–HOXD10 group; (2) UUO + AAV–Vector group; (3) UUO group; and (4) sham group. Each group included 10 mice. An RIF model was established via UUO in the mice during the 10th week. Briefly, UUO model mice were established by ligating the left lateral ureter, and the sham group underwent surgery that involved kidney exposure but not ligation. At 12 weeks, the mice were euthanized, and we collected blood and kidney tissue samples for related indexes detection.

### Biochemistry detection

The levels of blood urea nitrogen (BUN) and serum creatinine in mice were detected by an automatic biochemical analyzer (VITROS 950, Johnson & Johnson, NJ, USA).

### Kidney histopathological evaluation

Paraffin-embedded renal tissues from mice or humans were sectioned (3 µm thick) for Masson’s trichrome, picro-sirius red staining and hematoxylin and eosin (H&E). Sections underwent related staining as per the instructions provided with the kits. We then acquired the images with a Leica microscope. To analyze histopathological changes in the kidney, 10 high-magnification images (400×) were randomly chosen for each sample, and the scoring methodology utilized was derived from a prior investigation [32]. To analyze the fibrotic area, we randomly selected 10 high-magnification images (400×) from each sample. The blue or red area indicates the level of fibrosis based on the amount of collagen observed, which was assessed using image analysis software (Image-Pro Plus 6.0, Version X; Media Cybernetics, Silver Springs, MD, USA) to assign semiquantitative scores for the fibrotic areas.

### Chromatin immunoprecipitation (ChIP)

We performed ChIP in HK-2 cells by an anti-HOXD10 monoclonal antibody (sc-166235, Santa Cruz, CA, USA) or isotype mouse IgG (negative control) according to the protocol of the Simple ChIP Kit (91820S, Cell Signaling Technology, Danvers, MA, USA). We used specific primers to measure precipitated DNA by PCR for observing the binding of HOXD10 to NOX4 promoter (forward: 5ʹ*-*AATACCTGGCCTAAAGTCATATAGT-3ʹ; reverse: 3ʹ-TCCTAAGAATGCTTCATCTCAGTAA-5ʹ).

### Dual-luciferase reporter assay

The process was performed as our group’s previous report [33]. We provided the specific steps in the Supplementary Methods and included the restriction digestion map and vector backbone in Supplementary Fig. S1.

### Cell Counting Kit-8 (CCK-8) analysis

After transfecting cells with either empty plasmids or HOXD10 overexpression plasmids, they were treated with TGF-β1 for 24, 48 or 72 h. Subsequently, we added CCK-8 reagent (CK04-13; Dojindo, Kumamoto, Japan) to the medium, and incubated the cells for 1.5 h at 37 °C. We detected the absorbance by a microplate reader (BioTek, Vermont, USA).

### Bisulfite sequencing PCR (BSP) assay

The process was performed as previously described [34]. We provided the specific steps in the Supplementary Methods. PCR was performed (HOXD10-forward: 5ʹ-TTTGGAGGTTTTTAGAGTTGAGATT-3ʹ, HOXD10-reverse: 5ʹ-CACATAACAACCAAACCAATAAAAT-3ʹ). Agarose gel electrophoresis was employed to confirm the PCR products (Supplementary Fig. S2).

### Iron analysis

For HK-2 cells, we used an ultrasonic homogenizer to homogenize the collected cells. For mouse samples, we washed 15 mg of renal tissue from each group with 4 °C PBS and immediately homogenized the tissue with a vibrating homogenizer. An iron assay kit was purchased from Jiancheng Bioengineering Institute (A039-2-1, Nanjing, China) and was used to measure the relative iron concentrations in HK-2 cell lysates and renal tissue lysates. We measured the absorbance at 593 nm using a microplate reader (BioTek, Vermont, USA).

### qRT-PCR

The process was performed as our group’s previous report [33, 35, 36]. We provided the specific steps and the sequences of the primers in the Supplementary Methods and Table S1.

### Western blotting analysis

The process was performed as our group’s previous report [33, 35, 36]. We provided the specific steps and the antibody dilution ratios in the Supplementary Methods and Table S2.

### Immunohistochemical (IHC) staining

The process was performed as our group’s previous report [33]. We provided the specific steps and the antibody dilution ratios in the Supplementary Methods and Table S3.

### Immunofluorescence (IF) staining

The process was performed as our group’s previous report [33, 35, 36]. We provided the specific steps and the antibody dilution ratios in the Supplementary Methods and Table S4.

### Measurement of ROS Generation

H2-DCFDA staining was employed to assess ROS production in HK-2 cells. In brief, cells from each group were stained with 5 µM H2-DCFDA (Thermo Fisher, D399, Waltham, MA, USA) dissolved in PBS at room temperature for 20 min. Following staining, we use the fluorescence microscopy to analyze cells. Mitochondrial ROS were detected using MitoSOX staining, where cells were exposed to MitoSOX (5 µM, Thermo Fisher, M36007, Waltham, MA, USA) for 30 min and then examined under fluorescence microscopy. For ROS detection in renal tissue sections, DHE staining was utilized. Initially, the sections were deparaffinized and rehydrated. Subsequently, we employed dihydroethidium (20 µM, Beyotime Biotechnology, S0063, Shanghai, China) on the sections, and incubated them at room temperature for 15 min. Fluorescence microscopy was used to assess the sections, with nuclei stained by Hoechst dye.

### C11 staining

C11 staining with a C11 BODIPY 581/591 kit (Thermo Fisher, C10445, Waltham, MA, USA) was employed to evaluate lipid ROS generation in cells. We removed the cell culture media from each group after treatment and added 10 µM Image-iT® Lipid Peroxidation Sensor (Component A, Thermo Fisher, Waltham, MA, USA) to the cells and incubated the cells for 30 min at 37 °C, with nuclei stained using Hoechst dye. We washed the cells subsequently and evaluated lipid ROS generation via fluorescence microscopy.

### Measurement of renal oxidized glutathione/total glutathione (GSSG/GSH) ratio, superoxide dismutase (SOD) and malondialdehyde (MDA) levels

We purchased a GSH and GSSG Assay Kit from Beyotime (Shanghai, China) and SOD (A001-1) and MDA (A003-1) kits from Jiancheng Bioengineering Institute (Nanjing, China) to detect the levels of the ratio of GSSG to total GSH (reduced GSH + GSSG), SOD (hydroxylamine method) and MDA (thiobarbituric acid method) in fresh renal tissues. We utilized a microplate reader (BioTek, Vermont, USA) to measure the absorbance.

### Statistical analysis

We conducted the statistical analysis through GraphPad Prism 9.0 Software (San Diego, CA, USA) and SPSS 26.0 software (IBM, Armonk, New York, USA). Between groups, we use Student’s *t* test and one-way analysis of variance with post hoc tests to analyze the significance of differences. We presented the data as the means ± standard deviations of a minimum of three independent experiments. Statistically significant difference was presented by a value of *P* < 0.05. Each experiment was performed a minimum of three times independently.

## Results

### Low HOXD10 expression in renal biopsies from CKD patients

We recruited 20 CKD patients at The Fourth Hospital of China Medical University from July 2022 to May 2023. The average serum creatinine level was 462.3 ± 151.3 µmol/L, while the average eGFR was 15.2 ± 11.1 ml/min/1.73 m^2^. We measured HOXD10 expression in patients’ kidney biopsy specimen, and collected normal control kidney tissues from nephrectomies that were performed on tumor patients.

**Fig. 1:**
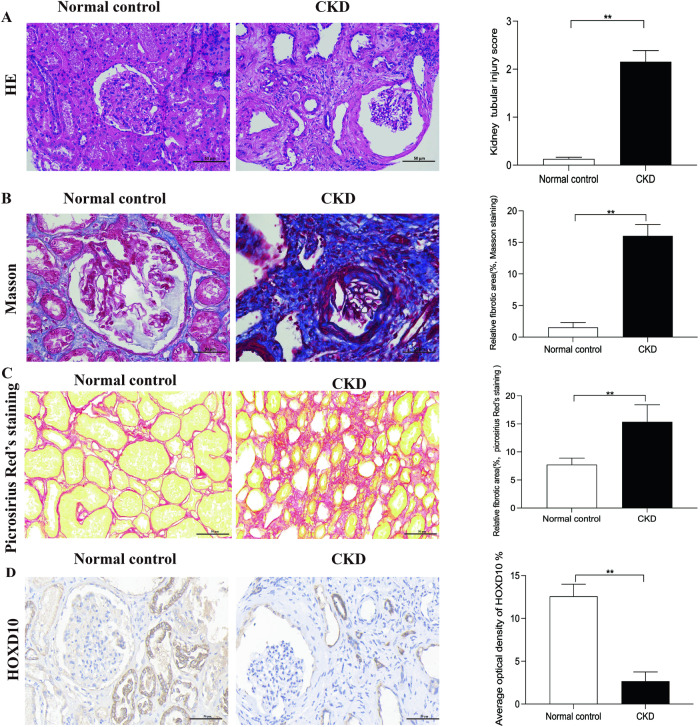
Expression of HOXD10 in renal biopsies. **A** H&E staining of kidney specimens from normal controls and CKD patients and relative tubular injury score. **B** Masson’s trichrome staining and semiquantitative analysis of average optical density of fibrotic area. **C** Picro-sirius red stainings and semiquantitative analysis of average optical density of fibrotic area. **D** IHC staining of HOXD10 protein in renal biopsies from normal controls and CKD patients and average optical density of HOXD10. The scale bar represents 50 µm. Magnification is 400×. Normal, normal kidney biopsies (*n* = 20). CKD, kidney biopsies from chronic kidney disease patients (*n* = 20). **P < 0.01.

H&E staining indicated renal pathological alterations in CKD patients, including tubular dilatation, interstitial edema, loss of brush border and epithelial desquamation (Fig. 1A). Picro-sirius red staining and Masson’s trichrome revealed a significant increase in collagen deposition, which indicated fibrosis in the kidneys of CKD patients (Fig. 1B, C). IHC staining revealed HOXD10 was mainly distributed in the tubulointerstitial area and a lower HOXD10 expression in CKD renal biopsies (Fig. 1D).

### TGF-β1 induces HOXD10 promoter hypermethylation and leads to the HOXD10 expression decreased in HK-2 cells

**Fig. 2:**
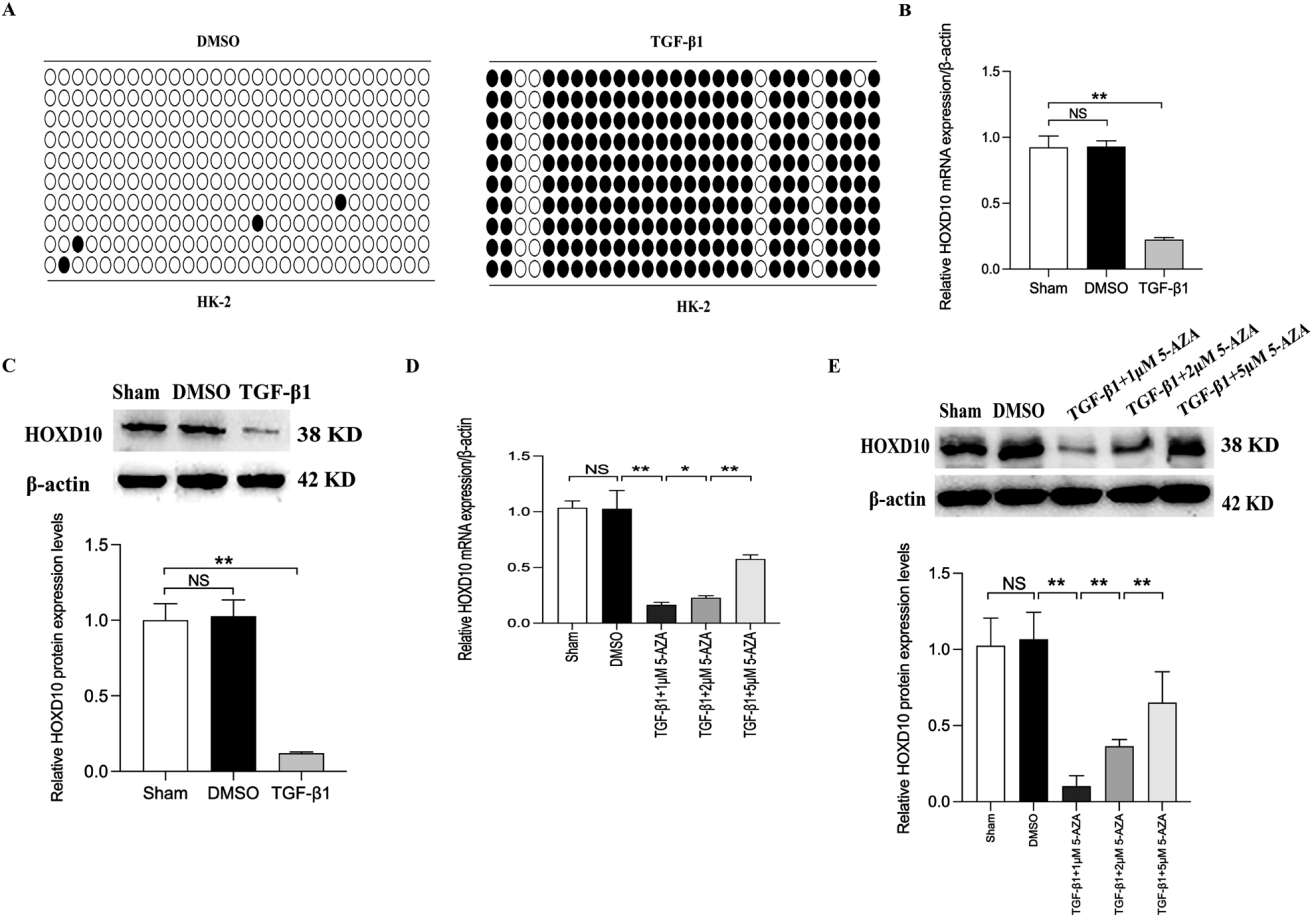
HOXD10 promoter hypermethylation leads to low HOXD10 expression in TGF-β1-induced HK-2 cells. **A** BSP results of the HOXD10 promoter hypermethylation in TGF-β1-induced HK-2 cells and normal control HK-2 cells. **B** qRT-PCR shows that the mRNAs of HOXD10 are downregulated in TGF-β1-induced HK-2 cells. **C** Western blot analysis shows that the proteins of HOXD10 are downregulated in TGF-β1-induced HK-2 cells. **D** qRT-PCR shows that the mRNAs of HOXD10 are recovered after treatment with demethylation drug 5-Aza for 48 h in TGF-β1-induced HK-2 cells. **E** Western blot analysis shows that the proteins of HOXD10 are recovered after treatment with demethylation drug 5-Aza for 48 h in TGF-β1-induced HK-2 cells. In all panels, the data are representative of three independent experiments. Data are presented as the mean ± SD. *P < 0.05, **P < 0.01, NS not significant.

In a variety of cancers, HOXD10 is recognized as a tumor suppressor gene, and the low expression of HOXD10 is regulated by the hypermethylation of its promoter region [21, 22]. BSP analysis demonstrated a notable increase in HOXD10 promoter methylation in TGF-β1-treated HK-2 cells but no methylation in normal control HK-2 cells (Fig. 2A). In TGF-β1-treated HK-2 cells, significantly downregulated mRNA and protein expression of HOXD10 was observed, and between the sham group and the DMSO group, there were no changes in the HOXD10 expression (Fig. 2B, C). Moreover, in both the cytoplasm and nucleus of HK-2 cells, IF showed that HOXD10 was expressed (Fig. S3). The current study assessed HOXD10 gene expression in TGF-β1-treated HK-2 cells both before and after treatment with a DNA methylation transferase inhibitor to further confirm the association between HOXD10 gene expression level and the methylation status of the HOXD10 gene. The mRNA and protein expression of HOXD10 was increased following exposure to 5-Aza (Fig. 2D, E). These findings imply a possible connection between DNA methylation and the methylation status of the HOXD10 promoter. These data suggest that TGF-β1 increases the degree of HOXD10 promoter methylation, which is followed by the suppression of HOXD10 expression in HK-2 cells.

### HOXD10 directly inhibits NOX4 transcriptional activity in vitro

**Fig. 3:**
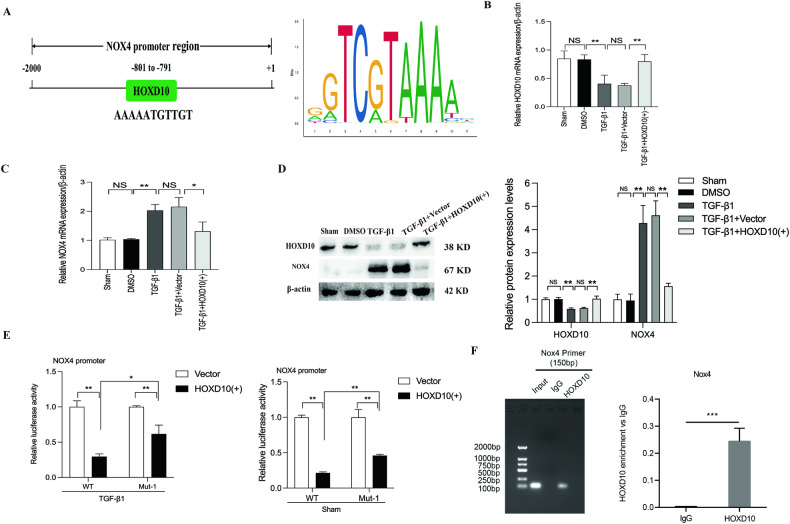
Regulatory effect of HOXD10 on the NOX4 promoter. **A** HOXD10 binding site on the promoter of NOX4 was predicted by the JASPAR tool. **B** Forty-eight hours after treatment with HOXD10 overexpression plasmids in TGF-β1-induced HK-2 cells, the mRNA expression levels of HOXD10 were assessed using qRT-PCR. **C** Forty-eight hours after treatment with HOXD10 overexpression plasmids in TGF-β1-induced HK-2 cells, the mRNA expression levels of NOX4 were assessed using qRT-PCR. **D** Forty-eight hours after treatment with HOXD10 overexpression plasmids in TGF-β1-induced HK-2 cells, the protein expression levels of HOXD10 and NOX4 were assessed using western blot analysis. **E** Luciferase reporter analysis elucidated the influence of HOXD10 overexpression on the luciferase activity of the NOX4 promoter reporter in each group. **F** ChIP analysis of the HOXD10 binding site. The DNA fragment corresponding to the promoter of NOX4 enriched by HOXD10 binding was evaluated by agarose gel electrophoresis or real-time quantitative PCR. In all panels, the data are representative of three independent experiments. Data are presented as the mean ± SD. *P < 0.05, **P < 0.01, ***P < 0.001, NS not significant.

In the NOX4 promoter, we predicted that HOXD10 binding sites exist by the JASPAR tool, indicating that HOXD10 may target NOX4 (Fig. 3A). To explore the effect of HOXD10 on NOX4 expression, we used HOXD10 overexpression plasmids to transfect HK-2 cells. The NOX4 mRNA and protein expression were significantly decreased by HOXD10 overexpression plasmids in TGF-β1-treated HK-2 cells (Fig. 3B–D). By luciferase reporter assay, we found that the NOX4 promoter luciferase activity was reduced by HOXD10 overexpression plasmids, and the luciferase activity was restored partly after mutation of the predicted site in HK-2 cells without or with TGF-β1 (Fig. 3E). These data proved that HOXD10 targeted the NOX4 promoter. The ChIP assay results further suggested that HOXD10 interacted with the promoter region of NOX4 (Fig. 3F). These results indicate that HOXD10 exerts a direct, negative influence on NOX4 transcription in HK-2 cells.

### HOXD10 overexpression inhibits ferroptosis, ECM accumulation and EMT in HK-2 cells induced by TGF-β1

**Fig. 4:**
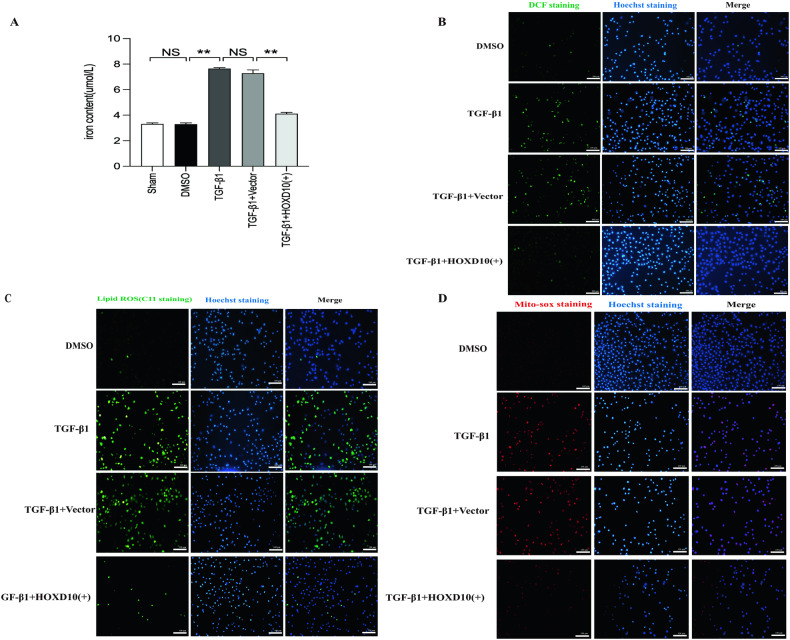
HOXD10 overexpression inhibits ferroptosis and oxidative stress in TGF-β1-induced HK-2 cells. **A** Iron concentrations in each HK-2 cells group. **B** DCF staining showed that HOXD10 overexpression plasmids alleviated TGF-β1-induced ROS generation in HK-2 cells (×200). Bar = 100 μm. **C** C11 staining showed that HOXD10 overexpression plasmids alleviated TGF-β1-induced lipid ROS generation in HK-2 cells (×200). Bar = 100 μm. **D** MitoSOX staining revealed that HOXD10 overexpression plasmids attenuated TGF-β1-induced mitochondrial ROS generation in HK-2 cells (×200). Bar = 100 μm. In all panels, the data are representative of three independent experiments. Data are presented as the mean ± SD. **P < 0.01, NS not significant.

**Fig. 5:**
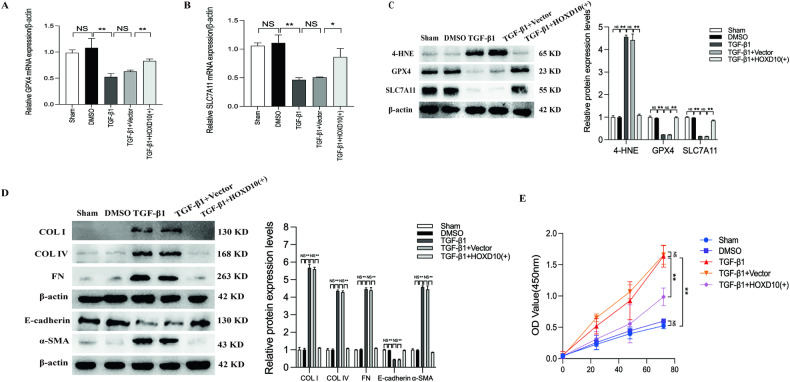
HOXD10 overexpression inhibits ferroptosis, ECM accumulation and EMT in TGF-β1-induced HK-2 cells. **A**, **B** Forty-eight hours after treatment with HOXD10 overexpression plasmids in TGF-β1-induced HK-2 cells, the mRNA expression levels of GPX4 and SLC7A11 were assessed using qRT-PCR. **C** Forty-eight hours after treatment with HOXD10 overexpression plasmids in TGF-β1-induced HK-2 cells, the protein expression levels of 4-HNE, GPX4 and SLC7A11 were assessed using western blot analysis. **D** Forty-eight hours after treatment with HOXD10 overexpression plasmids in TGF-β1-induced HK-2 cells, the protein expression levels of COL I, COL IV, FN, E-cadherin and α-SMA were assessed using western blot analysis. **E** After transfection with the HOXD10 overexpression plasmids, cell proliferation was detected with a CCK-8 assay at 0, 24, 48 and 72 h. In all panels, the data are representative of three independent experiments. Data are presented as the mean ± SD. *P < 0.05, **P < 0.01, NS not significant.

Ferroptosis is a type of iron-dependent cell death and is involved in kidney injury and fibrosis [37, 38]. In CKD, NOX4 is a modulator of ferroptosis in renal fibrosis process [27]; therefore, we speculated that HOXD10 may regulate renal fibrosis by regulating ferroptosis by targeting NOX4. The buildup of lipid peroxidation products, a hallmark of ferroptosis, necessitates a substantial quantity of iron for this. Thus, we first measured the iron content in HK-2 cells. The iron concentration was significantly greater in HK-2 cells treated with TGF-β1 compared to the sham group, and the HOXD10 overexpression plasmid treatment reduced the iron concentration in HK-2 cells treated with TGF-β1 (Fig. 4A). DCF and MitoSOX staining were employed to evaluate the status of oxidative stress, which is a key factor involved in ferroptosis. In addition, lipid ROS generation was assessed using C11 staining. Substantial increases in DCF-, C11- and MitoSOX-related fluorescence intensity were investigated in HK-2 cells treated with TGF-β1. Interestingly, all the increases in fluorescence intensity were attenuated by HOXD10 overexpression (Fig. 4B–D). The GPX4 and SLC7A11 mRNA and protein levels were both notably lower in HK-2 cells treated with TGF-β1 compared to the sham group. However, HOXD10 overexpression reversed these effects (Fig. 5A, B). Conversely, levels of 4-HNE increased under TGF-β1 treatment but decreased with HOXD10 overexpression (Fig. 5C). The ECM key components COL IV, COL I and FN and the EMT marker α-SMA were significantly increased in HK-2 cells treated with TGF-β1, which indicated a reversal of EMT and ECM accumulation and were markedly reduced by HOXD10 overexpression; furthermore, TGF-β1-induced HK-2 cell EMT was also reversed after HOXD10 overexpression, as the decreased protein levels of E-cadherin were restored by HOXD10 overexpression in HK-2 cells (Fig. 5D). Additionally, the results of CCK-8 assay demonstrated that the HK-2 cell proliferation induced by TGF-β1 was suppressed by HOXD10 overexpression (Fig. 5E). These results indicate that HOXD10 overexpression is highly effective at attenuating ferroptosis and limiting ECM accumulation and EMT in HK-2 cells treated with TGF-β1.

### HOXD10 overexpression ameliorated ECM accumulation and EMT by inhibiting ferroptosis in HK-2 cells

**Fig. 6:**
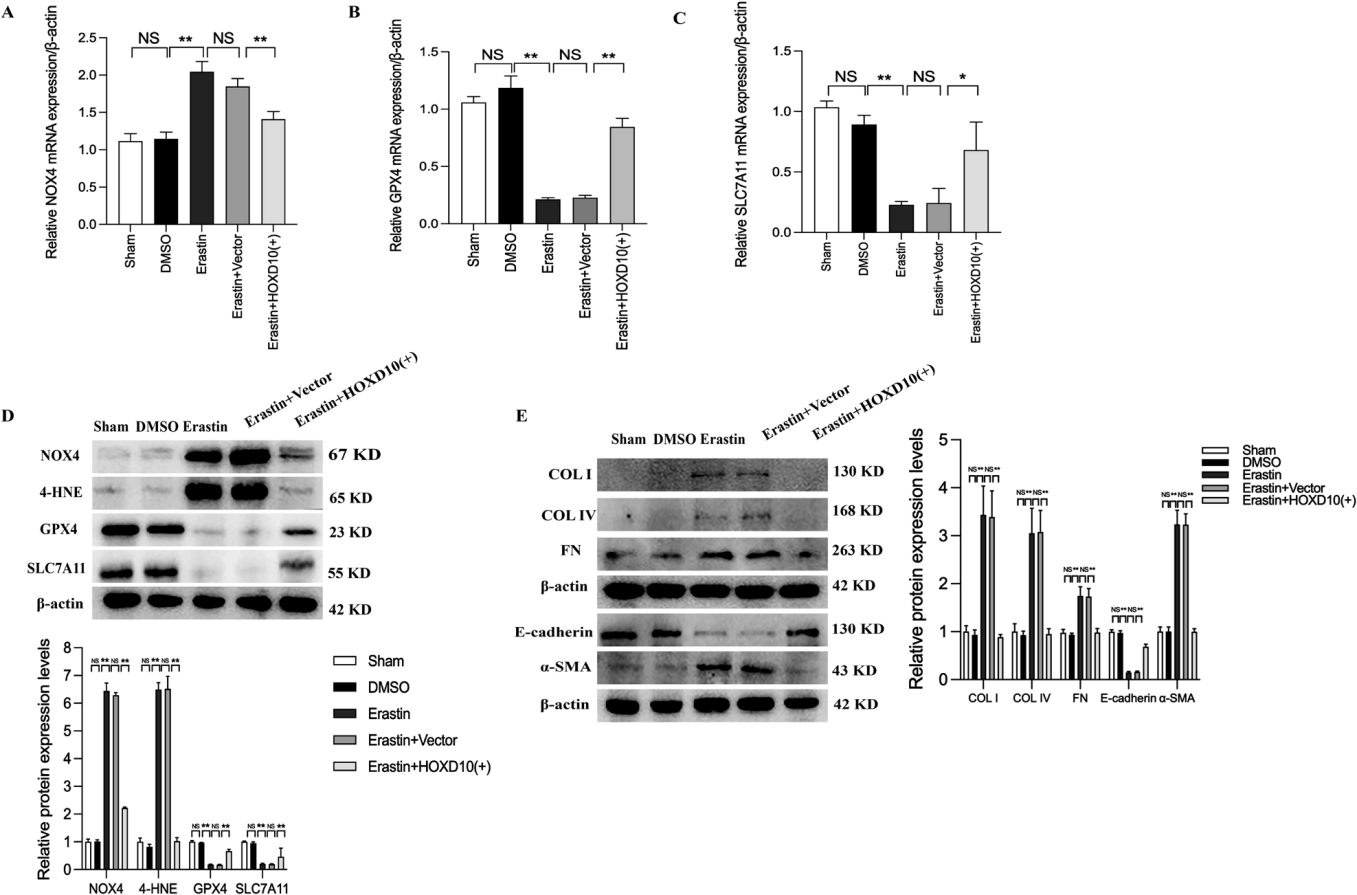
HOXD10 overexpression inhibits NOX4 transcription, ferroptosis, ECM accumulation and EMT in erastin-induced HK-2 cells. **A**–**C** Forty-eight hours after treatment with HOXD10 overexpression plasmids in erastin-induced HK-2 cells, the mRNA expression levels of NOX4, GPX4 and SLC7A11 were assessed using qRT-PCR. **D** Forty-eight hours after treatment with HOXD10 overexpression plasmids in erastin-induced HK-2 cells, the protein expression levels of NOX4, 4-HNE, GPX4 and SLC7A11 were assessed using western blot analysis. **E** Forty-eight hours after treatment with HOXD10 overexpression plasmids in erastin-induced HK-2 cells, the protein expression levels of COL I, COL IV, FN, E-cadherin and α-SMA were assessed using western blot analysis. In all panels, the data are representative of three independent experiments. Data are presented as the mean ± SD. *P < 0.05, **P < 0.01, NS not significant.

To explore the relationship between HOXD10 and ferroptosis and fibrosis, we used erastin, an agonist of ferroptosis, to establish a model of damage in HK-2 cells. The mRNA level of NOX4 was significantly higher compared to the sham group, and HOXD10 overexpression markedly reversed this effect in HK-2 cells treated with erastin (Fig. 6A). Similarly, GPX4 and SLC7A11 mRNA levels, which were decreased in erastin-treated cells, were restored by HOXD10 overexpression (Fig. 6B, C). The protein levels of NOX4 and 4-HNE were both notably upregulated compared to the sham group, and HOXD10 overexpression reversed these effects in HK-2 cells treated with erastin. Conversely, the protein levels of GPX4 and SLC7A11 exhibited a contrasting trend to those of NOX4 and 4-HNE (Fig. 6D). Moreover, the protein expression levels of COL IV, COL I, FN and α-SMA were significantly increased in HK-2 cells treated with erastin, but the levels of these molecules were significantly reduced by HOXD10 overexpression. Moreover, erastin-induced HK-2 cell EMT was also reversed by HOXD10 overexpression, as the decrease in the protein expression of E-cadherin was reversed in HOXD10-overexpressing HK-2 cells (Fig. 6E). These findings suggested that HOXD10 overexpression ameliorated ferroptosis, EMT and fibrosis in erastin-treated HK-2 cells.

To confirm the potential impact of ferroptosis inhibition on fibrosis in HK-2 cells, we utilized ferrostatin-1, a known ferroptosis inhibitor, to validate the role of ferroptosis in TGF-β1-induced fibrosis in HK-2 cells. As shown in Supplementary Fig. S4A–E, ferrostatin-1 treatment significantly suppressed the upregulation of NOX4, 4-HNE, COL I, COL IV, FN and α-SMA and reversed the decreases in GPX4, SLC7A11 and E-cadherin expression in HK-2 cells treated with TGF-β1. These findings indicated that ferroptosis inhibition may be a key factor that suppresses fibrosis in the context of ECM accumulation induced by TGF-β1. Collectively, these findings suggest that elevated levels of HOXD10 may potentially inhibit ECM accumulation and EMT in HK-2 cells through suppressing ferroptosis.

### HOXD10 overexpression reduces kidney injury and collagen deposition in UUO model mice

**Fig. 7:**
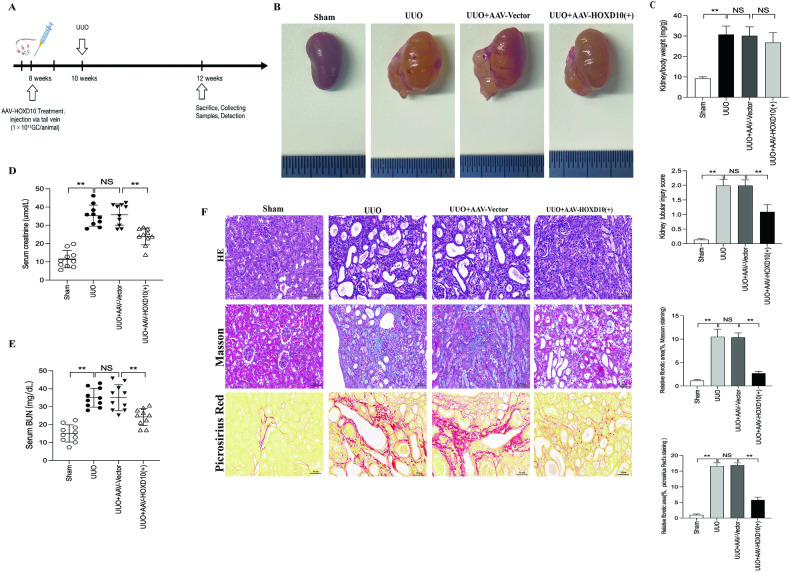
HOXD10 overexpression restores renal function and kidney injury in UUO mice. **A** Schematic of the mouse experimental protocol. **B** Morphology of the kidney samples. **C** Ratio of obstructed kidney/body weight in mice. **D** Serum creatinine and **E** Serum BUN in various groups at 12 weeks of age. **F** Representative images of H&E, Masson’s trichrome and picro-sirius red stainings and relative tubular injury score and relative fibrotic area of each group (×400). Bar = 50 μm. In all panels, the data are representative of three independent experiments. Data are presented as the mean ± SD of each mouse (*n* = 10 mice in each group). **P < 0.01, NS not significant.

To evaluate whether HOXD10 overexpression could protect the kidney from injury, we established an obstructive nephropathy model, which is characterized by inflammation and fibrosis, in mice [39]. The injection of AAV vectors encoding HOXD10 via the tail vein in 8-week-old mice induced the HOXD10 overexpression, followed by UUO surgery at 10 weeks (Fig. 7A). We collected kidney samples at 12 weeks of age (Fig. 7B). Immunofluorescence staining confirmed successful AAV-mediated GFP expression in the renal tubulointerstitium (Supplementary Fig. S5). The kidney weight/body weight ratio (kidney index, KI) was higher in the UUO group than in the sham group. But AAV–HOXD10 (+) treatment had no effect on the KI of the UUO group (Fig. 7C). Serum creatinine and BUN levels significantly increased in the UUO group compared to the sham group. The significant reversal of this effect was accomplished by HOXD10 overexpression (Fig. 7D, E). Moreover, histological analysis using H&E staining revealed that AAV–HOXD10 (+) treatment significantly ameliorated renal tubular dilation, interstitial edema, tubular epithelial cell necrosis/loss and brush border loss. Similarly, as shown by picro-sirius red staining and Masson’s trichrome, AAV–HOXD10 (+) dramatically attenuated collagen deposition and fibrosis in the tubulointerstitial area of UUO model mice (Fig. 7F). These results reveal that HOXD10 overexpression may improve renal dysfunction and reduce kidney injury in UUO model mice.

### HOXD10 overexpression transcriptionally represses NOX4 expression in UUO model mice

**Fig. 8:**
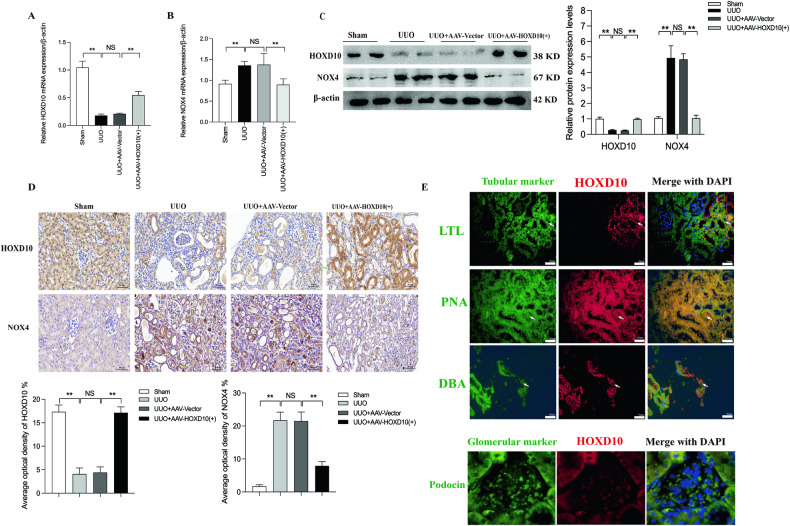
Effect of HOXD10 overexpression on the expression of NOX4 in UUO mice. **A**, **B** The effect of AAV–HOXD10 on the mRNA expression of HOXD10 and NOX4 in the renal cortical tissues was detected using qRT-PCR analysis. **C** The effect of AAV–HOXD10 on the protein expression of HOXD10 and NOX4 in the renal cortical tissues was detected using western blot analysis. **D** IHC detection of HOXD10 and NOX4 in mice kidney tissues of each group and average optical density of each index (×400). Bar = 50 μm. **E** Dual immunofluorescent staining for HOXD10 and various segment-specific tubular markers as well as glomerular marker podocin in kidneys from sham group mice. The following segment-specific tubular markers were used: proximal tubule, lotus tetragonolobus lectin (LTL); distal tubule, peanut agglutinin (PNA); and collecting duct, dolichos biflorus agglutinin (DBA). Arrows indicate positive tubules with colocalization of HOXD10 and specific tubular markers. Bar = 50 μm. In all panels, the data are representative of three independent experiments. Data are presented as the mean ± SD of each mouse (*n* = 10 mice in each group). **P < 0.01, NS not significant.

We evaluated the expression of NOX4 post-HOXD10 overexpression in UUO mice to investigate the physiological function of HOXD10. The mRNA level of HOXD10 significantly decreased in renal cortical tissue lysates from UUO model mice compared to sham mice, with a notable increase after HOXD10 overexpression (Fig. 8A). Conversely, NOX4 mRNA exhibited an opposite trend to that of HOXD10 (Fig. 8B). Similarly to the changes in mRNA expression, the protein expression of HOXD10 significantly increased and the protein expression of NOX4 significantly decreased after AAV–HOXD10 (+) treatment (Fig. 8C). IHC staining revealed lower expression levels of HOXD10 and higher expression levels of NOX4 in the renal tissues of UUO model mice than in those of the sham group. In UUO model mice, AAV–HOXD10 (+) reversed the renal low HOXD10 expression and the high NOX4 expression. Additionally, IHC results revealed that HOXD10 was expressed in both the tubulointerstitial and glomerulus areas. However, compare to the glomerulus area, HOXD10 was more strongly expressed in the tubulointerstitial area (Fig. 8D). We use respective segment-specific tubular cell markers—dolichos biflorus agglutinin (a collecting duct epithelium marker), peanut agglutinin (a distal convoluted tubule marker) and lotus tetragonolobus lectin (a proximal tubule marker) to costain HOXD10 by dual immunofluorescence in kidney tissues from sham mice. Moreover, we conducted dual immunofluorescence to costain HOXD10 with the glomerular marker podocin, and the results showed that HOXD10 showed almost no costaining with podocin, which indicated that very little HOXD10 located in the glomerular area. All these results showed that HOXD10 was enriched in the renal tubulointerstitial area and that it was localized in distal and proximal tubular segments as well as the collecting duct (Fig. 8E). In UUO mice kidney, these results indicate that HOXD10 overexpression may inhibit NOX4 expression transcriptionally.

### HOXD10 overexpression attenuated ferroptosis, EMT and fibrosis in the renal tissues of UUO model mice

**Fig. 9:**
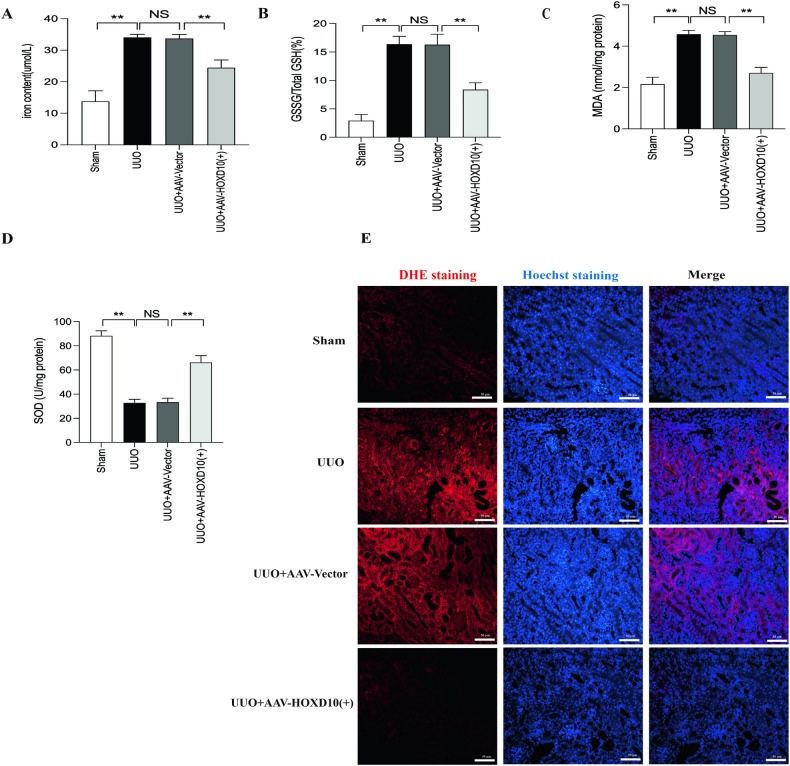
HOXD10 overexpression reduced ferroptosis and oxidative stress in UUO mice. **A** Iron concentrations in the kidney tissue lysates. **B** GSSG/total GSH ratio and **C** MDA and **D** SOD levels in the kidney tissues of each group. **E** DHE staining showed that AAV–HOXD10 alleviates ROS generation in UUO mice kidneys (×400). Bar = 50 μm. In all panels, the data are representative of three independent experiments. Data are presented as the mean ± SD of each mouse (*n* = 10 mice in each group). **P < 0.01, NS not significant.

To investigate the physiological function of HOXD10 in ferroptosis and fibrosis in UUO model mice, we detected the expression of ferroptosis and fibrosis markers after HOXD10 overexpression in UUO model mice. In UUO model mice, renal iron concentrations were found to be significantly higher compared to sham mice. However, treatment with AAV–HOXD10 (+) reduced renal iron levels (Fig. 9A). In the UUO group, the GSSG/GSH ratio and MDA content were both significantly elevated compared to sham mice. However, the administration of AAV–HOXD10 (+) in the UUO group successfully blocked this increase (Fig. 9B, C). In contrast, SOD levels were markedly lower in the UUO group and AAV–HOXD10 (+) treatment reversed the downregulation of SOD expression (Fig. 9D). DHE staining showed increased ROS generation in the UUO group, which was attenuated by AAV–HOXD10 (+) treatment (Fig. 9E).

**Fig. 10:**
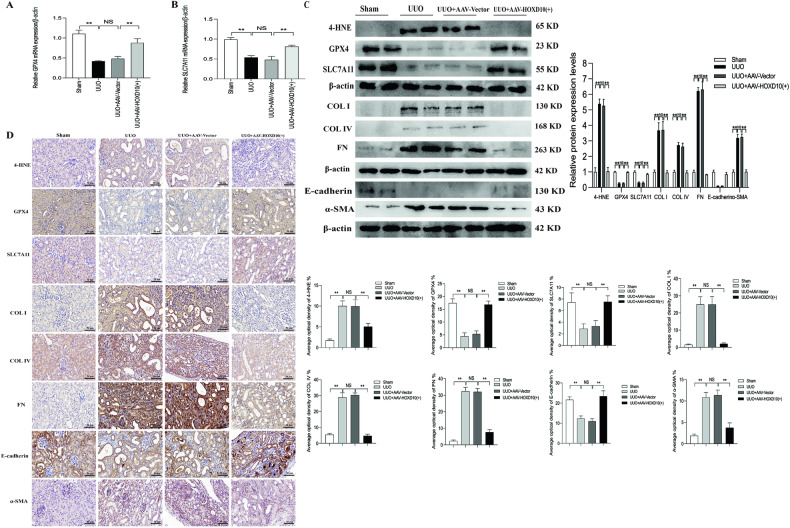
HOXD10 overexpression reduced ferroptosis, renal EMT and fibrosis in UUO mice. **A, B** The effect of AAV–HOXD10 on the mRNA expression of GPX4 and SLC7A11 in the renal cortical tissues was detected using qRT-PCR analysis. **C** The effect of AAV–HOXD10 on the protein expression of 4-HNE, GPX4, SLC7A11, COL I, COL IV, FN, E-cadherin and α-SMA in renal cortical tissues from various groups was detected using western blot analysis. **D** IHC detection of 4-HNE, GPX4, SLC7A11, COL I, COL IV, FN, E-cadherin and α-SMA in mice kidney tissues of various groups at the end of the study and average optical density of each index. (×400). Bar = 50 μm. In all panels, the data are representative of three independent experiments. Data are presented as the mean ± SD of each mouse (*n* = 10 mice in each group). **P < 0.01, NS not significant.

The mRNA levels of both GPX4 and SLC7A11 were significantly lower in the renal tissues of UUO model mice compared to sham mice. However, following treatment with AAV–HOXD10 (+) treatment, these alterations were reversed (Fig. 10A, B). Western blotting analysis revealed that the protein expression of 4-HNE was decreased, while the protein expression of GPX4, SLC7A11 and E-cadherin was increased by AAV–HOXD10 (+) treatment; moreover, the protein expression of COL I, COL IV, FN and α-SMA in the renal tissues of UUO model mice was decreased by AAV–HOXD10 (+) treatment (Fig. 10C). Similarly to the alterations in protein expression, IHC staining revealed high expression levels of 4-HNE, COL I, COL IV, FN and α-SMA and low expression levels of GPX4, SLC7A11 and E-cadherin in renal tissues of UUO model mice compared to the sham group. AAV–HOXD10 (+) suppressed the changes in the expression of these indicators in the renal tissues of UUO model mice (Fig. 10D). In UUO model mice, these results suggest that HOXD10 overexpression attenuates renal pathologies including renal ferroptosis, EMT and fibrosis.

## Discussion

We provided the initial evidence suggesting that HOXD10 plays a protective role against the kidney injury that is caused by ferroptosis and renal fibrosis in UUO model mice and HK-2 cells in this study. Increased intracellular iron concentrations lead to ferroptosis, resulting in elevated ROS levels [40]. The main source of renal ROS production is predominantly mediated by NADPH oxidases [41]. The current study revealed a marked increase in ROS production and iron overload, and HOXD10 overexpression reduced ROS production and iron overload in UUO model mouse kidneys and HK-2 cells induced by TGF-β1. This effect was accompanied by a significant increase in NOX4 expression, and HOXD10 overexpression suppressed NOX4 expression in UUO model mouse kidneys and HK-2 cells induced by TGF-β1. In UUO-induced renal fibrosis, a common condition is renal tubulointerstitial injury. In this study, we also investigated severe tubulointerstitial injuries (such as interstitial edema, brush border loss, tubular epithelial cell necrosis/loss and renal tubular dilation) in UUO model mice similar to previous studies [39]. HOXD10 overexpression effectively alleviated UUO-induced tubular injury and deterioration of renal histology, resulting in reduced creatinine and BUN levels. In TGF-β1-treated HK-2 cells, moreover, the HOXD10 gene was aberrantly hypermethylated and expressed at low levels. Through our mechanistic studies, we identified that HOXD10 may be an NOX4 transcription factor and negatively regulates NOX4 expression by targeting the NOX4 promoter. Moreover, HOXD10 overexpression inhibited the expression of ferroptosis- and fibrosis-related genes in a rodent model of UUO and in HK-2 cells induced by TGF-β1, revealing potential mechanism underlying CKD-related renal fibrosis. These findings indicated that the increase in NADPH oxidase-derived ROS production in CKD-related renal EMT and fibrosis was prevented by inhibiting ferroptosis via HOXD10 overexpression. Our data suggest that HOXD10 may be a novel target for alleviating renal fibrosis and renal tubular injury.

HOXD10 is an ANTP homeobox gene that is located on chromosome 2 (2p31). This gene cluster is divided into four groups (HOX A, B, C and D), and HOX regulates embryonic development and stem cell differentiation [42, 43]. Particularly, HOXD10 is a crucial gene responsible for regulating nephrogenesis and developmental processes [24, 25]. Many studies have reported that HOXD10 is regulated by epigenetics, as shown by the aberrant hypermethylation of its promoter, which leads to its low expression in tumor tissues or cells; moreover, HOXD10 serves as a tumor suppressor gene in several malignancies [21, 22]. The aberrant hypermethylation of the HOXD10 promoter has been confirmed to be related to lung fibrosis [26], but the relationship between HOXD10 and CKD-related fibrosis is currently unknown. In this study, we first found that in CKD-related fibrosis models, the HOXD10 promoter was aberrantly hypermethylated, and HOXD10 gene expression was low. Moreover, following treatment with the demethylation agent 5-Aza, there was an elevation in HOXD10 expression. Our data showed that HOXD10 is regulated by epigenetics during the progression of CKD-related fibrosis.

In kidney diseases, the involvement of multiple cell death mechanisms presents key targets for preventing tissue damage. Research on ferroptosis, a novel form of cell death, has mainly focused on its implications for AKI. Studies have revealed that ferroptosis inhibitors are effective against kidney injury in vivo and in vitro experiments [44, 45]. Nonetheless, the direct relationship between renal fibrosis and ferroptosis in CKD progression remains unclear. Ferroptosis is accompanied by inhibition of the glutamate–cystine antiporter system, xCT, and followed by decreased intracellular GSH levels and lipid ROS accumulation, which ultimately result in redox imbalance. Lipid ROS can accumulate via either the impaired detoxification of lipid peroxidation by reducing the expression of GPX4 or via the production of superoxide and hydrogen peroxide resulting from the upregulation of NOXs [46–49]. More precisely, 4-HNE is a primary end product of lipid peroxidation, and variations in its levels indirectly reflect the extent of tissue damage caused by peroxidation [50–52]. MDA is the primary aldehyde product resulting from lipid peroxidation, while SOD serves as a crucial antioxidant enzyme. Recent studies have verified increased MDA levels, increased GSSG/GSH ratios and decreased SOD levels in UUO model mice kidneys compared with that of control mice [37]. In the NOX family, NOX4 enrich in the renal proximal tubular cells, and it is responsible for controlling cellular ROS production and accumulation and has been reported to facilitate ferroptosis [53–55]. Accumulating evidence suggests that ROS can trigger ferroptosis, and decreasing NOX4 expression can prevent ferroptosis by reducing ROS production in a variety of diseases, including cardiac ischemia and reperfusion and Alzheimer’s disease [55–58]. Ferroptosis and oxidative stress promote ECM (FN, COL IV and COL I) accumulation and EMT (E-cadherin and α-SMA) in the kidneys of db/db and UUO model mice [59–61]. Furthermore, several studies have reported that NOX4 inhibition can mitigate renal fibrosis and inflammation via the ROS pathway in UUO and diabetic kidney injury mouse models [62–64]. Thus, NOX4 may inhibit EMT and renal fibrosis by regulating ROS production and ferroptosis.

In this research, we first confirmed that NOX4 is a new target by which HOXD10 performs its function. Our data demonstrated that HOXD10 targets the NOX4 promoter directly and regulates NOX4 expression in HK-2 cells negatively. This study presents the initial evidence of a correlation between HOXD10 and NOX4. Moreover, our study also showed that HOXD10 enriched in the tubulointerstitial area with the expression in both the cytoplasm and nucleus. After overexpression of HOXD10 in HK-2 cells induced by TGF-β1/erastin and in kidneys of UUO model mice, NOX4 transcription and ferroptosis were inhibited, as indicated by changes in ROS, lipid ROS, the GSSG/GSH ratio, MDA, and SOD levels and the amelioration of renal dysfunction, renal pathological injury, tubulointerstitial EMT and fibrosis. Thus, our data suggest that HOXD10 may inhibit CKD-related fibrosis progression by suppressing NOX4 gene expression, which ultimately inhibits ROS production and ferroptosis and ameliorates tubular injury and tubulointerstitial EMT as well as fibrosis in CKD-related fibrosis models.

In the pathophysiology of renal fibrosis, despite we first pointed out that HOXD10 plays a key role, there also have limitations in this work. For example, in other cell patterns (podocytes, endothelial cells and mesangial cells), the role of HOXD10 in CKD can not be excluded by our data. Additionally, to explore the role of HOXD10 in other CKD, further studies are needed to be performed such as in mesangial cells proliferation and podocyte injury. Additionally, this research only found the preventive effects of HOXD10 in an UUO model, but the treatment effects should also be fully explored. In the current study, we confirmed that the HOXD10 gene was aberrantly hypermethylated and expressed at low levels in the context of CKD-related fibrosis. HOXD10 negatively regulates NOX4 expression. Therefore, low HOXD10 expression in the context of CKD-related fibrosis can increase NOX4 expression and promote ferroptosis subsequently, which finally leads to tubular injury and tubulointerstitial fibrosis (see Graphical abstract).

Our research posits that in CKD-related fibrosis, an aberrant hypermethylation pattern suppresses HOXD10 expression, leading to elevated NOX4 levels which could enhance ferroptosis and consequent renal damage. These findings considered HOXD10 as a novel therapeutic target in CKD-related fibrosis treatment strategies. By overexpression of HOXD10, NOX4 transcription is reduced, which in turn inhibits ROS production and ferroptosis, eventually alleviating renal tubule dysfunction and injury as well as tubulointerstitial fibrosis in CKD patients. In the future, HOXD10 may also be used as a new therapeutic target for CKD-related fibrosis.

### Supplementary information

supplementary material

WB GEL1

## Data Availability

All datasets in the present study can be obtained from the corresponding author upon request.
