# Supplementary material for: HOXD10 attenuates renal fibrosis by inhibiting NOX4-induced ferroptosis
Source: Cell Death Dis. 2024 Jun 6;15(6):398. doi: 10.1038/s41419-024-06780-w (PMC11156659; doi:10.1038/s41419-024-06780-w)
Supplement: Supplementary file 1 — supplementary material [file 41419_2024_6780_MOESM1_ESM.docx]

**
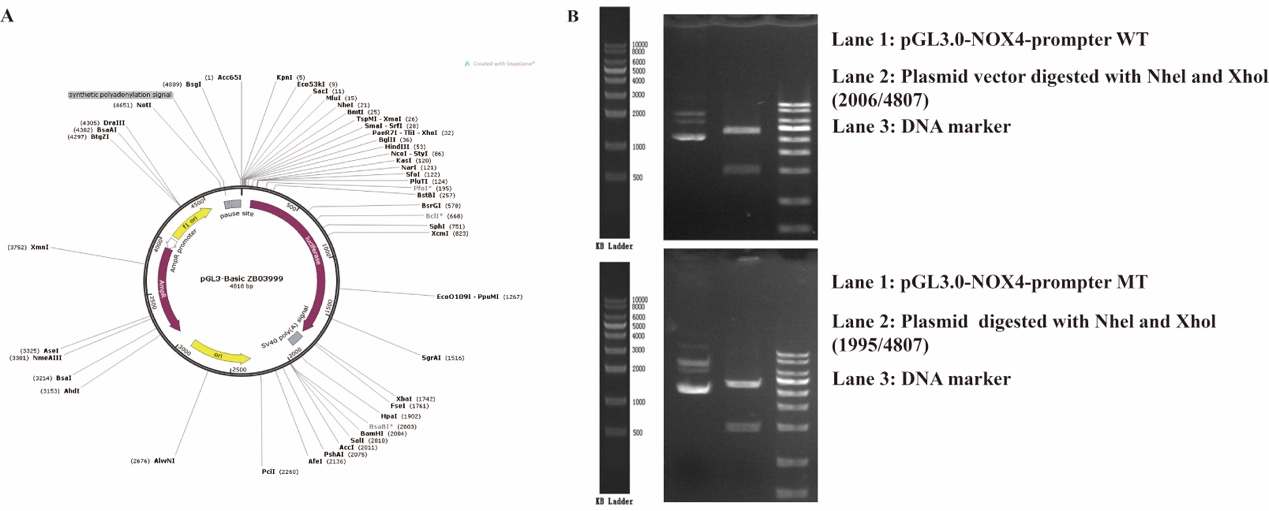
**

**Figure S1. HOXD10-promoter-luc plasmid backbone (A) and restriction digestion map (B).**

**
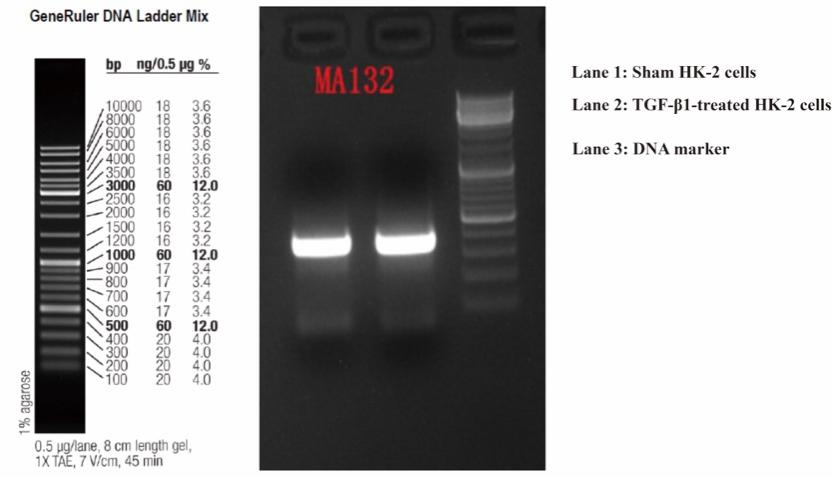
**

**Figure S2. Agarose gel electrophoresis of PCR products for BSP assay. Note: MA132: sample ID.**

**
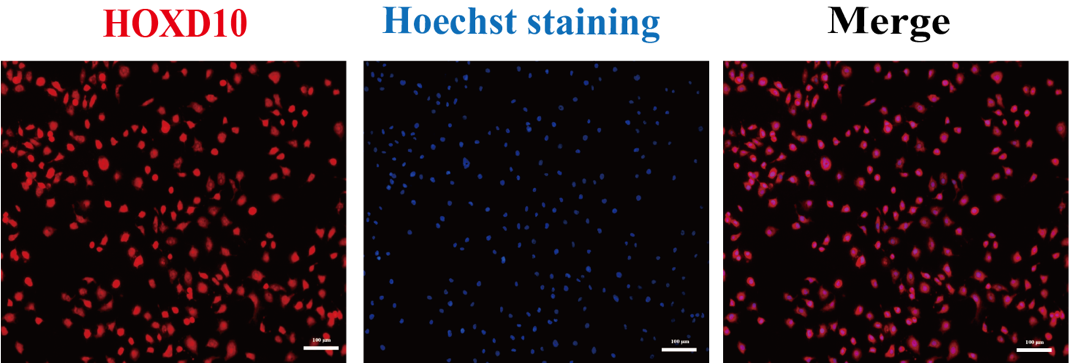
**

**Figure S3. Fluorescence microscopy images to detect HOXD10 expression in HK-2 cells. Bar=100μm**

**
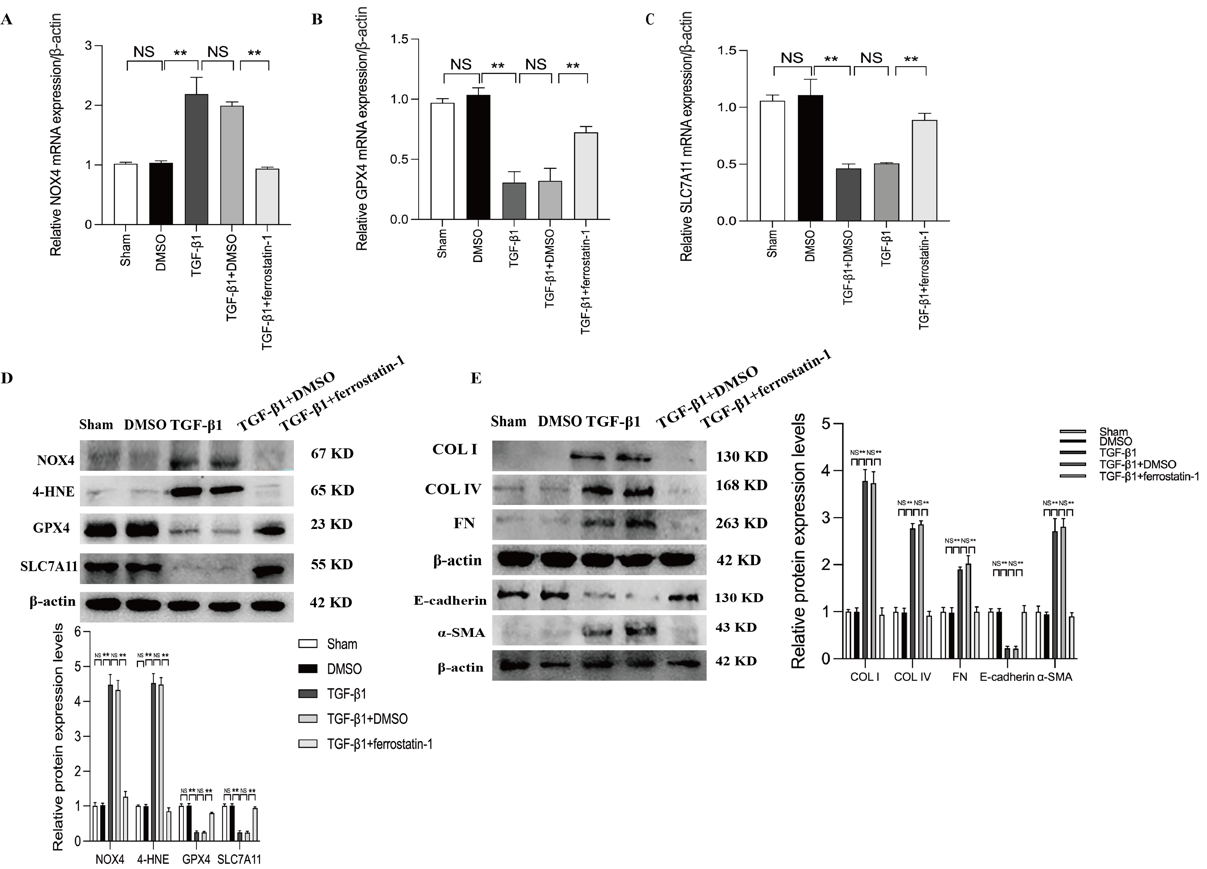
**

**Figure S4.** Ferrostatin-1could inhibit ferroptosis, ECM accumulation and EMT in TGF-β1-induced HK-2 cells. (A-C) Forty-eight hours after treatment with ferrostatin-1 in TGF-β1-induced HK-2 cells, the mRNA expression levels of NOX4, GPX4 and SLC7A11 were assessed using qRT–PCR. (D-E) Forty-eight hours after treatment with ferrostatin-1 in TGF-β1-induced HK-2 cells, the protein expression levels of NOX4, 4-HNE, GPX4, SLC7A11, COL I, COL IV, FN, E-cadherin and α-SMA were assessed using western blot analysis. In all panels, the data are representative of three independent experiments. Data are presented as the mean ± SD of each mouse (n=10 mice in each group). **P* < 0.05, ***P* < 0.01, NS, not significant.

**
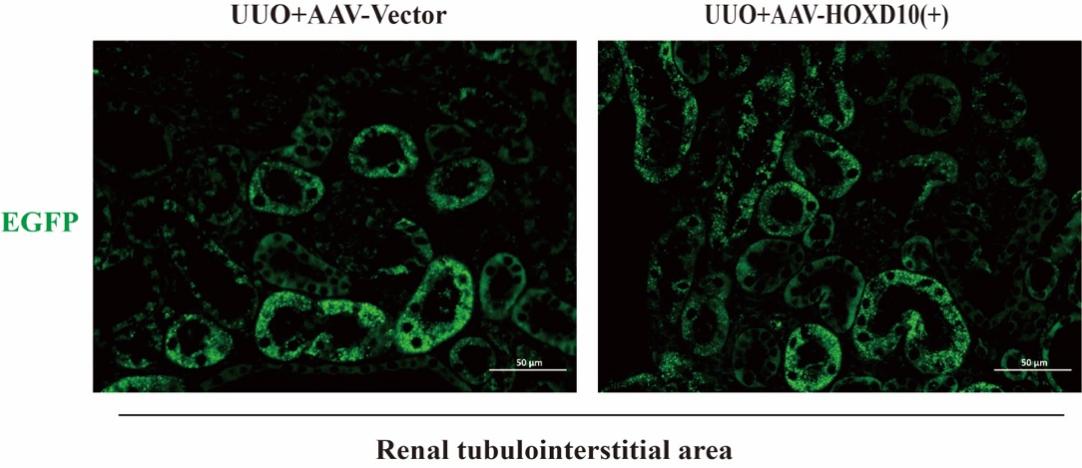
**

**Figure S5. Fluorescence microscopy images to detect green fluorescent protein (GFP) expression of AAV-Vector and AAV-HOXD10(+) in mice renal tubule interstitial area. Bar=50μm**

**Supplementary Table S1.** **Primer sequences of qRT-PCR**

| **Gene** | **Primer sequence（5’-3’）** |
| --- | --- |
| HOXD10  （mouse） | Reverse: TGGCGGTCAGTTCTCGGATTC  Forward: CCCTTACACCAAGCACCAAACG |
| HOXD10  （human） | Reverse: TGGTGGTTCACTTCTCTTTTGG  Forward: GACATGGGGACCTATGGAATGC |
| NOX4  （mouse） | Reverse: GCTGGTTCGGTTAAGACTGATGC  Forward: TGGTGGTGGTGCTATTCCTCATG |
| NOX4  （human） | Reverse: TGGATGTTCACAAAGTCAGGTCTG  Forward: TGGCTGGAGGCATTGGAGTC |
| GPX4  （mouse） | Reverse: ACGCAGCCGTTCTTATCAAT  Forward: CCGGCTACAATGTCAGGTTT |
| GPX4  （human） | Reverse: CCTCCTCCTTAAACGCACAC  Forward: AGATCCACGAATGTCCCAAG |
| SLC7A11  （mouse） | Reverse: GCCTACCATGAGCAGCTTTC  Forward: GATGCTGTGCTTGGTCTTGA |
| SLC7A11  （human） | Reverse: CCTGGGTTTCTTGTCCCATA  Forward: CCCAGATATGCATCGTCCTT |
| ACTB  （mouse） | Reverse: CACAGCTTCTCTTTGATGTCAC  Forward: CTACCTCATGAAGATCCTGACC |
| ACTB  （human） | Reverse: CGAGCTCTGAGCACTGGAGA  Forward: TGGCGTGTAAAGTCACCACC |

**Supplementary Table S2. The specific antibody source and dilution ratios of Western blot**

| **Antibody** | **Dilution ratio** | **Source** |
| --- | --- | --- |
| anti-HOXD10 | 1:1000 | ab138508, Abcam, USA |
| anti-NOX4 | 1:2000 | 14347-1-AP, Proteintech Co Ltd, USA |
| anti-4-HNE | 1:1000 | ab46545, Abcam, USA |
| anti-GPX4 | 1:2000 | 67763-1-Ig, Proteintech Co Ltd, USA |
| anti-SLC7A11 | 1:1000 | 26864-1-AP, Proteintech Co Ltd, USA |
| anti-Collagen I | 1:2000 | 14695-1-AP, Proteintech Co Ltd, USA |
| anti-Collagen IV | 1:2000 | ab6586, Abcam, USA |
| anti-Fibronectin | 1:2000 | 15613-1-AP, Proteintech Co Ltd, USA |
| anti-E-cadherin | 1:30000 | 20874-1-AP, Proteintech Co Ltd, USA |
| anti-α-SMA | 1:2000 | 14395-1-AP, Proteintech Co Ltd, USA |

**Supplementary Table S3.** **The primary antibody source and dilution ratios of IHC**

| **Antibody** | **Dilution ratio** | **Source** |
| --- | --- | --- |
| anti-HOXD10 | 1:100 | sc-166235, SANTA CRUZ, USA |
| anti-NOX4 | 1:200 | 14347-1-AP, Proteintech Co Ltd, USA |
| anti-4-HNE | 1:25 | ab48506, Abcam, USA |
| anti-GPX4 | 1:2000 | 67763-1-Ig, Proteintech Co Ltd, USA |
| anti-SLC7A11 | 1:100 | 26864-1-AP, Proteintech Co Ltd, USA |
| anti-Collagen I | 1:500 | 14695-1-AP, Proteintech Co Ltd, USA |
| anti-Collagen IV | 1:200 | ab6586, Abcam, USA |
| anti-Fibronectin | 1:500 | 15613-1-AP, Proteintech Co Ltd, USA |
| anti-E-cadherin | 1:10000 | 20874-1-AP, Proteintech Co Ltd, USA |
| anti-α-SMA | 1:3000 | 14395-1-AP, Proteintech Co Ltd, USA |

**Supplementary Table S4.** **The primary antibody source and dilution ratios of IF staining**

| **Antibody** | **Dilution ratio** | **Source** |
| --- | --- | --- |
| anti-Lotus Tetragonolobus Lectin (LTL) | 20μg/ml | FL-1321; VECTOR Laboratories,USA |
| anti-Peanut Agglutinin (PNA) | 20μg/ml | FL-1071; VECTOR Laboratories,USA |
| anti-Dolichos Biflorus Agglutinin (DBA) | 20μg/ml | FL1031; VECTOR Laboratories,USA |
| anti-HOXD10 | 1:100 | sc-166235, SANTA CRUZ, USA |
| anti-Podocin | 1:300 | 20384-1-AP, Proteintech Co Ltd, USA |

**Supplementary Methods**

**Adeno-associated virus (AAV) transfection**

We purchased adeno-associated virus (AAV) 2/9-HOXD10 (pAAV-ITR-CAG-Hoxd10 (mouse)-IRES-EGFP-WPRE-Sv40 polyA-ITR) and normal control AAV2/9-Vector (pAAV-ITR-CAG-MCS-IRES-EGFP-WPRE-Sv40 polyA-ITR) from SyngenTech (Beijing, China). The volume of the AAV recombinant vectors that was used for the mice experiments was 100 μL[10^11^ genome copies (GCs)/animal]. Recombinant AAV vectors or vehicle (PBS) were injected by the tail vein at the 8th week. We used qRT–PCR and Western blotting to measure the transfection efficiency.

**Dual-luciferase reporter assay**

The potential HOXD10 binding sites in the NOX4 promoter region were predicted by the JASPAR tool. Cells were cultured in 24-well plates. NOX4 wild-type (WT)/mutant (Mut) luciferase plasmids were constructed by inserting NOX4 promoter fragments containing WT or Mut HOXD10 binding sites into the pGL3.0 reporter vector (Sangon, Shanghai, China). After 48 hours of cotransfection with HOXD10 overexpression plasmids or empty vectors, we performed a reporter gene assay using a Dual Luciferase Assay System (Promega, Madison, WI, USA).

**Bisulfite Sequencing PCR (BSP) assay**

Briefly, we firstly used a DNeasy Blood & Tissue Kit (QIAGEN, Duesseldorf, Germany) according to the manufacturer’s protocol to isolate DNA from HK-2 cells that were treated with or without TGF-β1. Sequences that included 2,000 bp from upstream of the transcription initiation site to downstream of the transcription initiation site in the promoter regions were downloaded from NCBI. We employed the UCSC Genome Browser for predicting promoter-related CpG islands. Genomic DNA was exposed to sodium bisulfite, and PCR was performed by the following primers: HOXD10-Forward 5'-TTTGGAGGTTTTTAGAGTTGAGATT-3', and HOXD10-Reverse 5'-CACATAACAACCAAACCAATAAAAT-3'. We purified and recovered amplified bisulfate PCR products by a SanPre Column PCR Purification Kit (Sangon, Shanghai, China) and then subcloned and inserted into the pUC18-T vector system. Ten individual clones were used to obtain DNA sequencing.

**qRT–PCR**

We extracted total RNA from mouse kidneys and HK-2 cells using a TRIzol Reagent Kit (Invitrogen, Carlsbad, CA, USA). RNA was reverse transcribed into cDNA using a GoScript^TM^ Reverse Transcription System (Promega, Madison, WI, USA) according to the manufacturer’s protocol. The CFX96 PCR System was used to detect the mRNA expression levels (Bio-Rad, Hercules, CA, USA) with GoTaq® qPCR Master Mix (Promega, Madison, WI, USA). Relative mRNA expression was normalized to that of β-actin via the 2^-ΔΔCt^ method.

**Western blotting analysis**

Mouse renal tissues and HK-2 cells were lysed in RIPA buffer containing a protease inhibitor cocktail (Roche Diagnostics, Basel, Switzerland). The BCA kit was used to detect the protein contents (Thermo Scientific, Waltham, MA, USA). Protein samples were separated by SDS‒PAGE and transferred to PVDF membranes (Millipore, Boston, USA). 5% BSA-TBST was used to block the membranes for 1.5 h at room temperature, followed by incubation with primary antibodies overnight at 4 °C. The next day, the membranes were incubated with HRP-conjugated secondary antibodies at 37 °C for 1 h. ECL (Millipore, Boston, USA) was used to visualize the levels of the proteins on the membranes. ImageJ software was used to semiquantify protein expression, and β-actin was used as the loading control.

**Immunohistochemical (IHC) staining**

Deparaffinized and rehydrated sections (3 μm) were subjected to routine antigen retrieval and incubated with 3% H_2_O_2_ for 10 min to block endogenous peroxidase activity. 5% BSA was used to block these sections and then the sections were incubated with primary antibodies overnight at 4 °C. Next, the sections were incubated with secondary antibodies for 2 h. DAB was added for visualization. After restaining the nuclei, we sealed the sections with neutral gum and obtained the images under a light microscope (400×, Nikon, Tokyo, Japan).

**Immunofluorescence (IF) staining**

For mice kidney tissues, paraffin-embedded mouse kidney sections (3 μm) were prepared by a routine procedure. The sections were deparaffinized, rehydrated and then heated in sodium citrate buffer (0.01 mol/L, pH 6.0). After blocking with goat serum, the slides were incubated with primary antibodies overnight at 4 °C. Next, the slides were incubated with fluorescently labeled secondary antibodies at room temperature for 1 h, and the nuclei were stained with DAPI. For HK-2 cells, cells were fixed with 4% paraformaldehyde, blocked with goat serum and incubated with primary antibody overnight at 4°C. After washing with PBS, fluorescent-labeled secondary antibody was added. Hoechst was used to stain the nuclei. We used a fluorescence microscope to capture images and analyzed images by the LAS AF Lite (Leica, Wetzlar, Germany).
