# Supplementary material for: HOXD10 attenuates renal fibrosis by inhibiting NOX4-induced ferroptosis
Source: Cell Death Dis. 2024 Jun 6;15(6):398. doi: 10.1038/s41419-024-06780-w (PMC11156659; doi:10.1038/s41419-024-06780-w)

Figure 2C

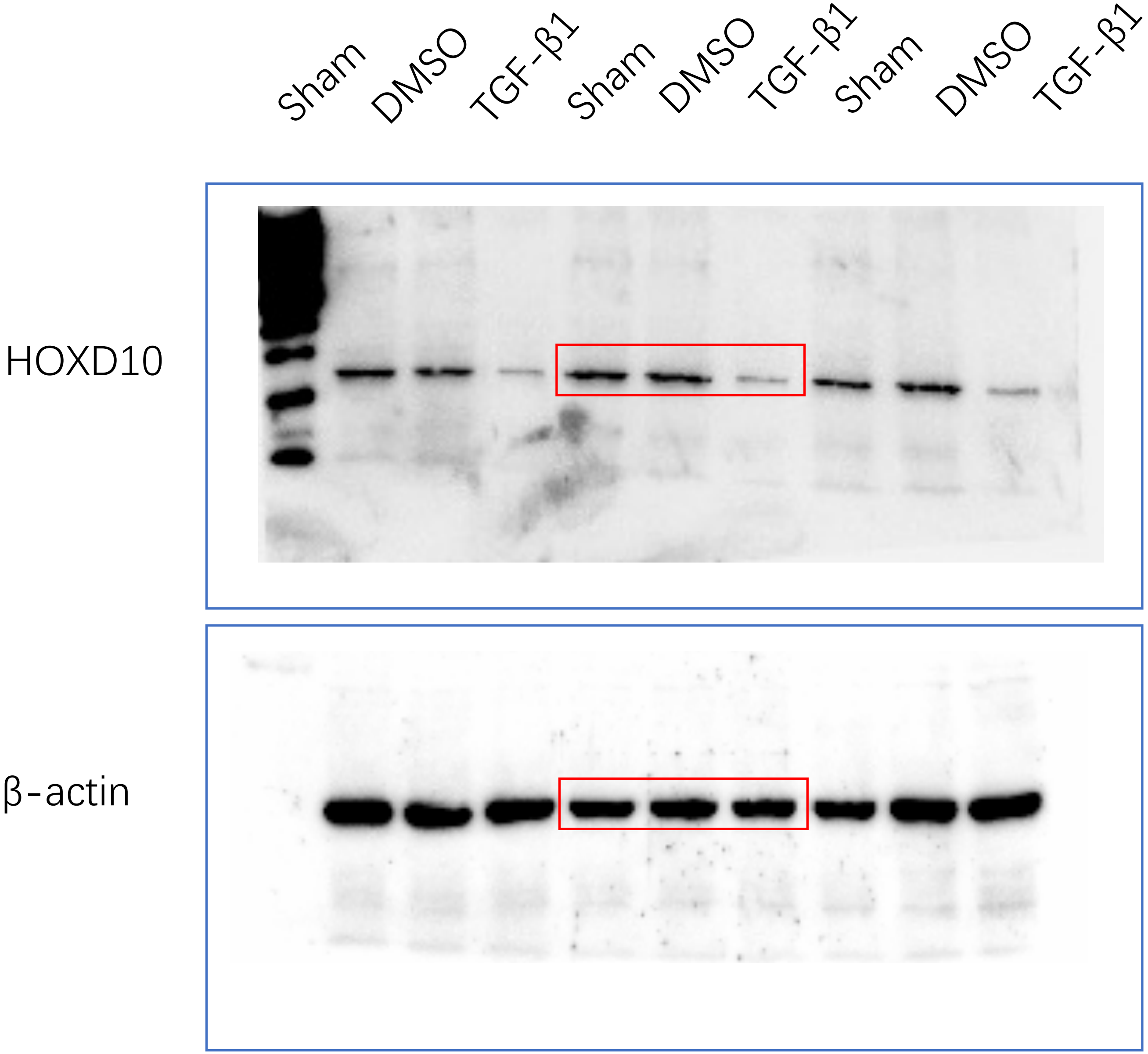

Figure 2E

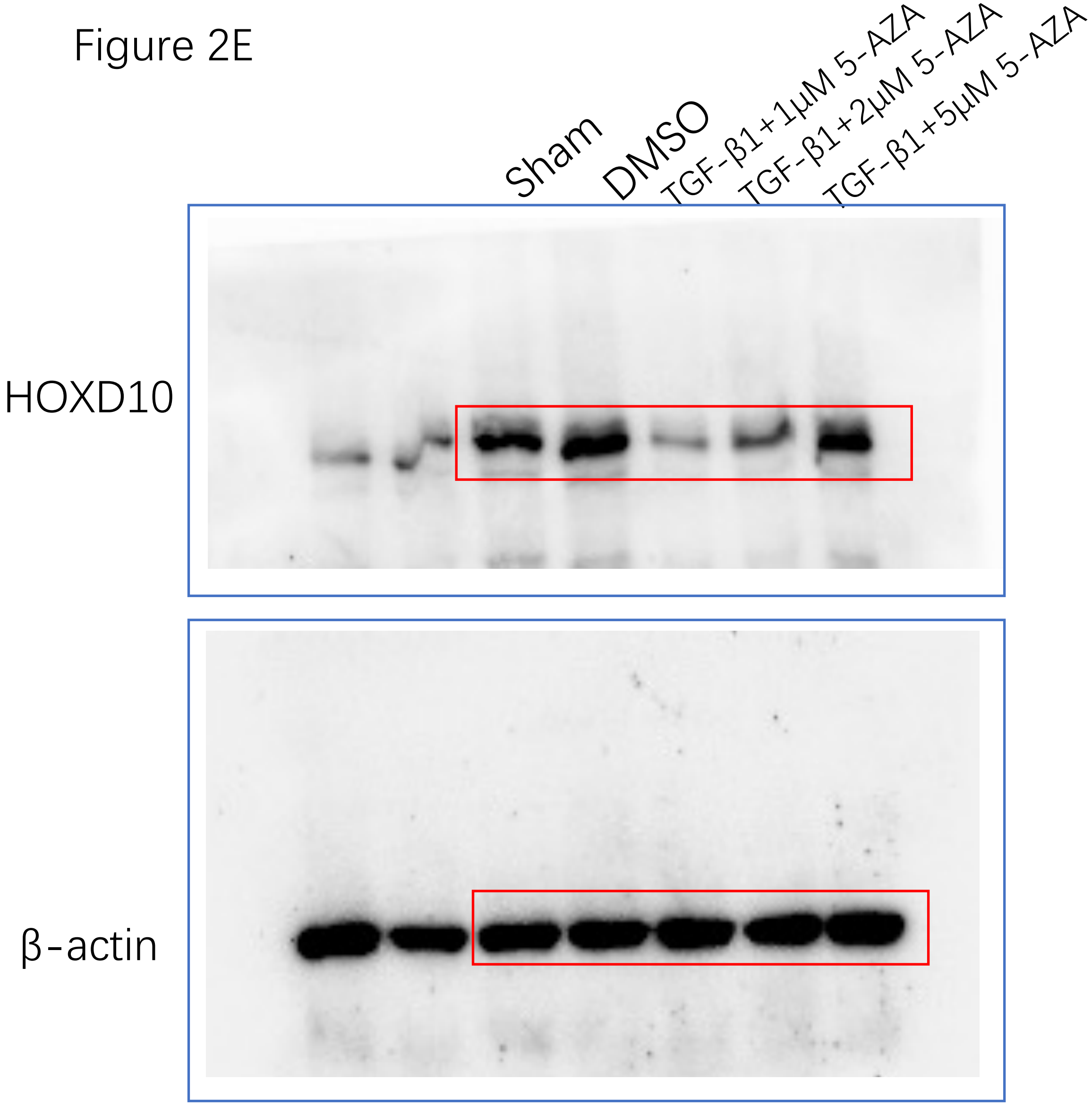

Figure 2E

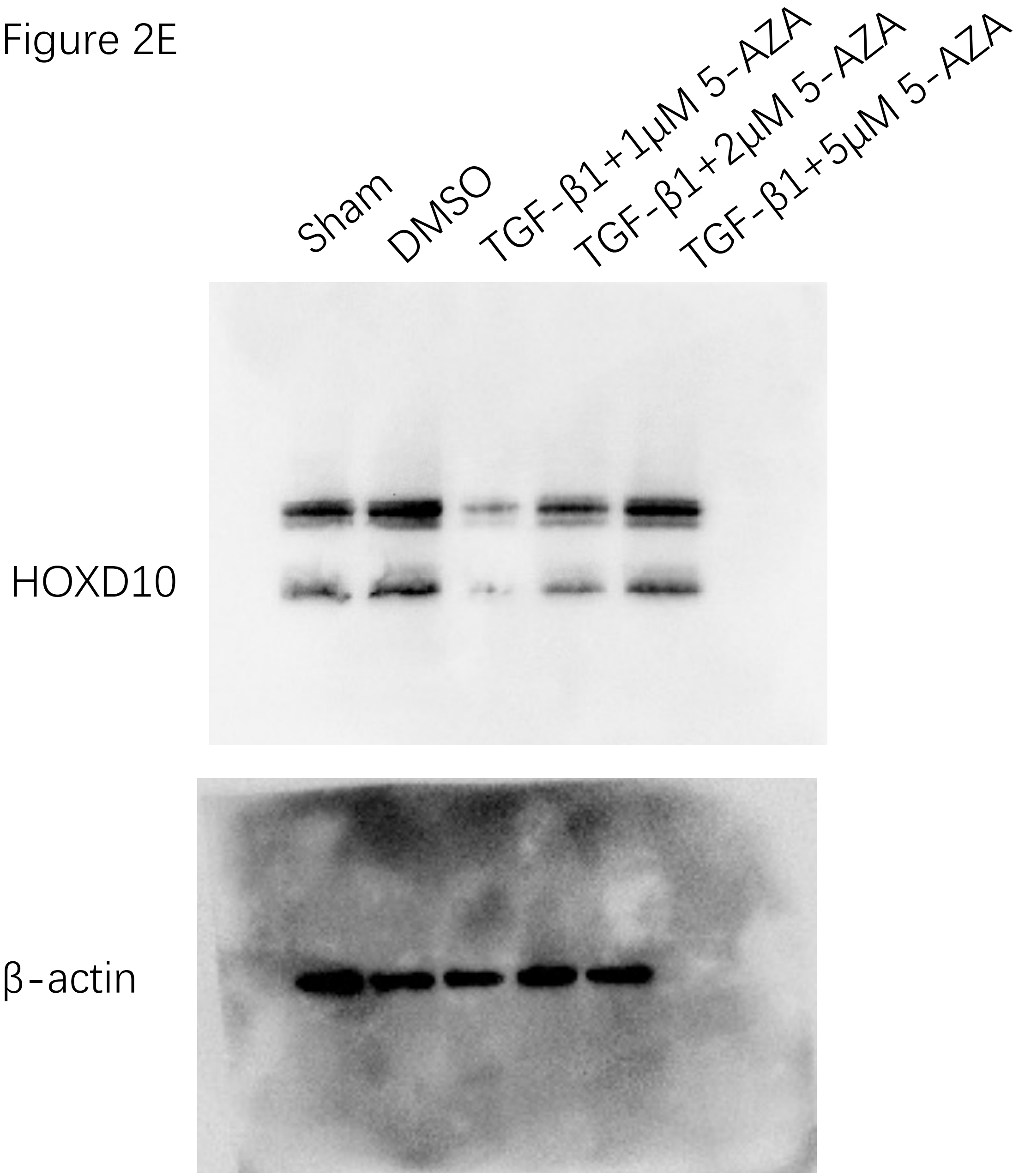

Sham
DMSO
TGF-β1+1μM 5-AZA
TGF-β1+2μM 5-AZA
TGF-β1+5μM 5-AZA
HOXD10
β-actin

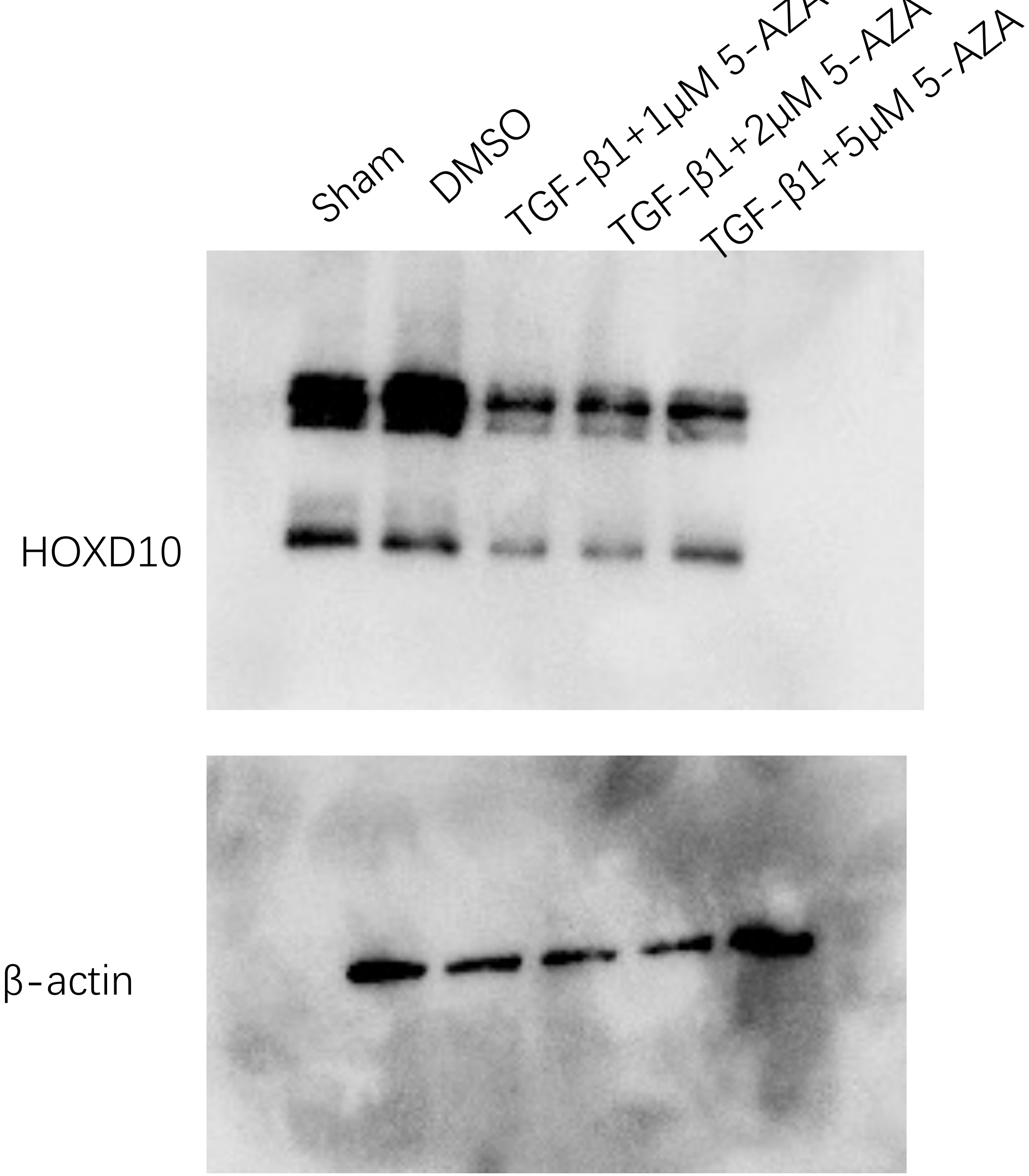

Sham
DMSO
TGF-β1+1μM 5-AZA
TGF-β1+2μM 5-AZA
TGF-β1+5μM 5-AZA
HOXD10
β-actin

Figure 3D

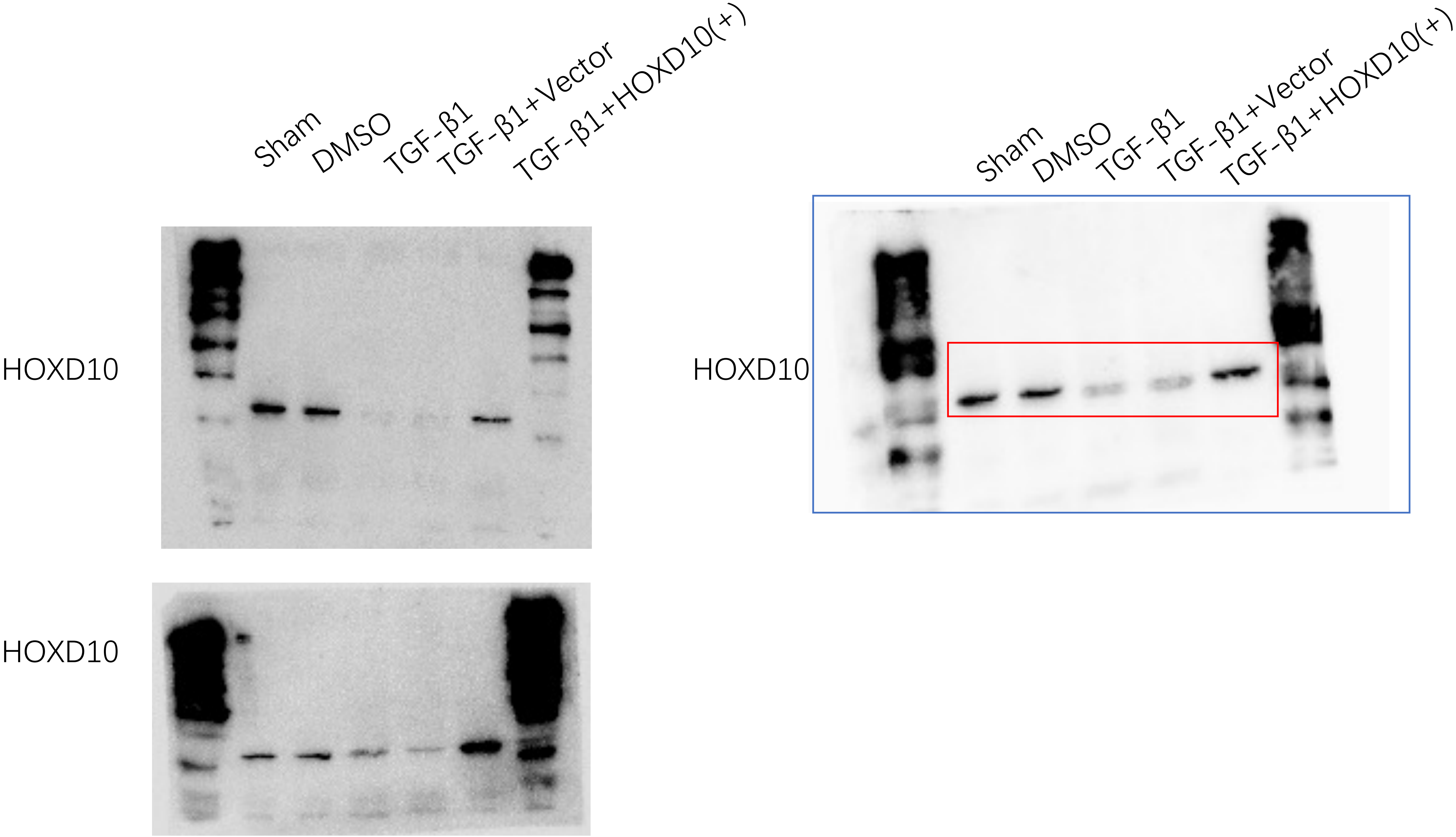
Sham
DMSO
TGF-β1
TGF-β1+Vector
TGF-β1+HOXD10(+)
HOXD10
HOXD10
Sham
DMSO
TGF-β1
TGF-β1+Vector
TGF-β1+HOXD10(+)
HOXD10

Figure 3D

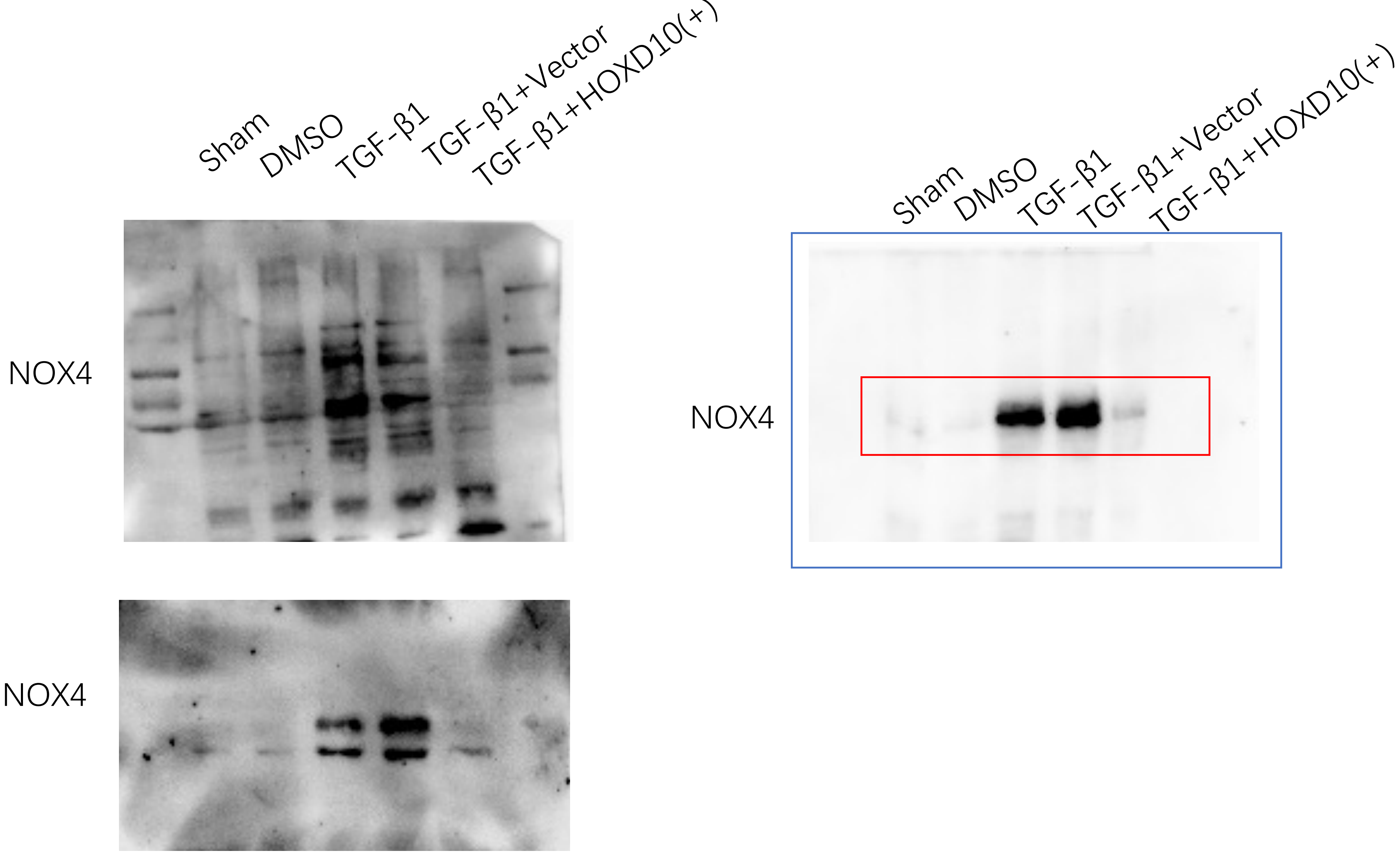
Sham
DMSO
TGF-β1
TGF-β1+Vector
TGF-β1+HOXD10(+)
NOX4
NOX4
Sham
DMSO
TGF-β1
TGF-β1+Vector
TGF-β1+HOXD10(+)
NOX4

Figure 3D

Sham DMSO TGF-β1 TGF-β1+Vector TGF-β1+HOXD10(+)

β-actin

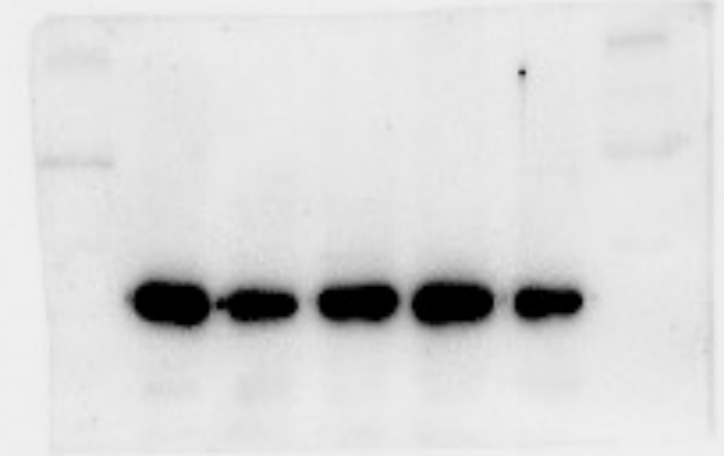

β-actin

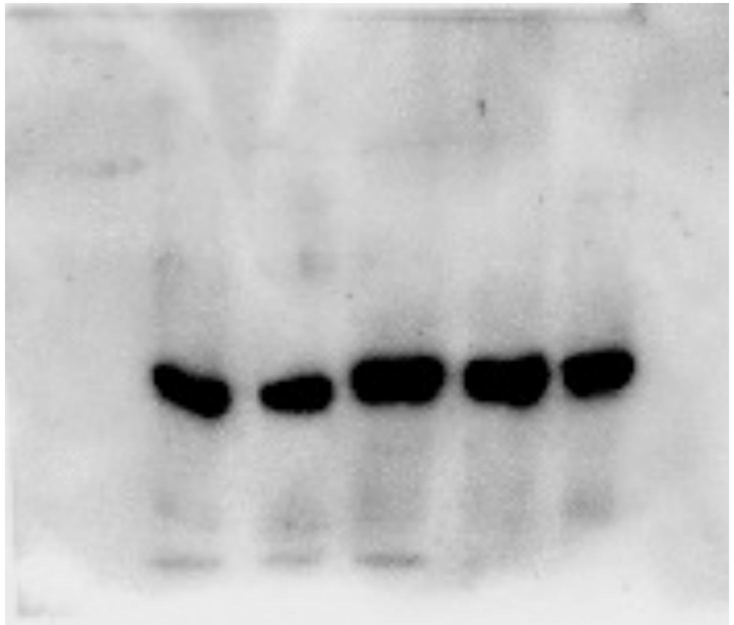

Sham DMSO TGF-β1 TGF-β1+Vector TGF-β1+HOXD10(+)

β-actin

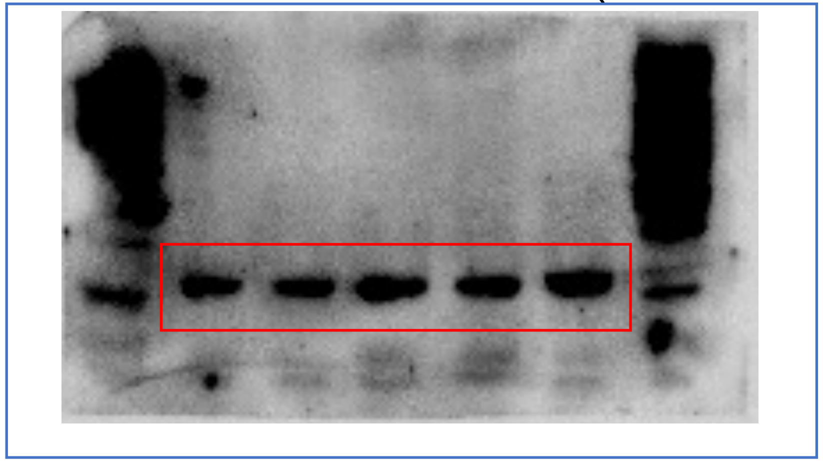

Figure 5C

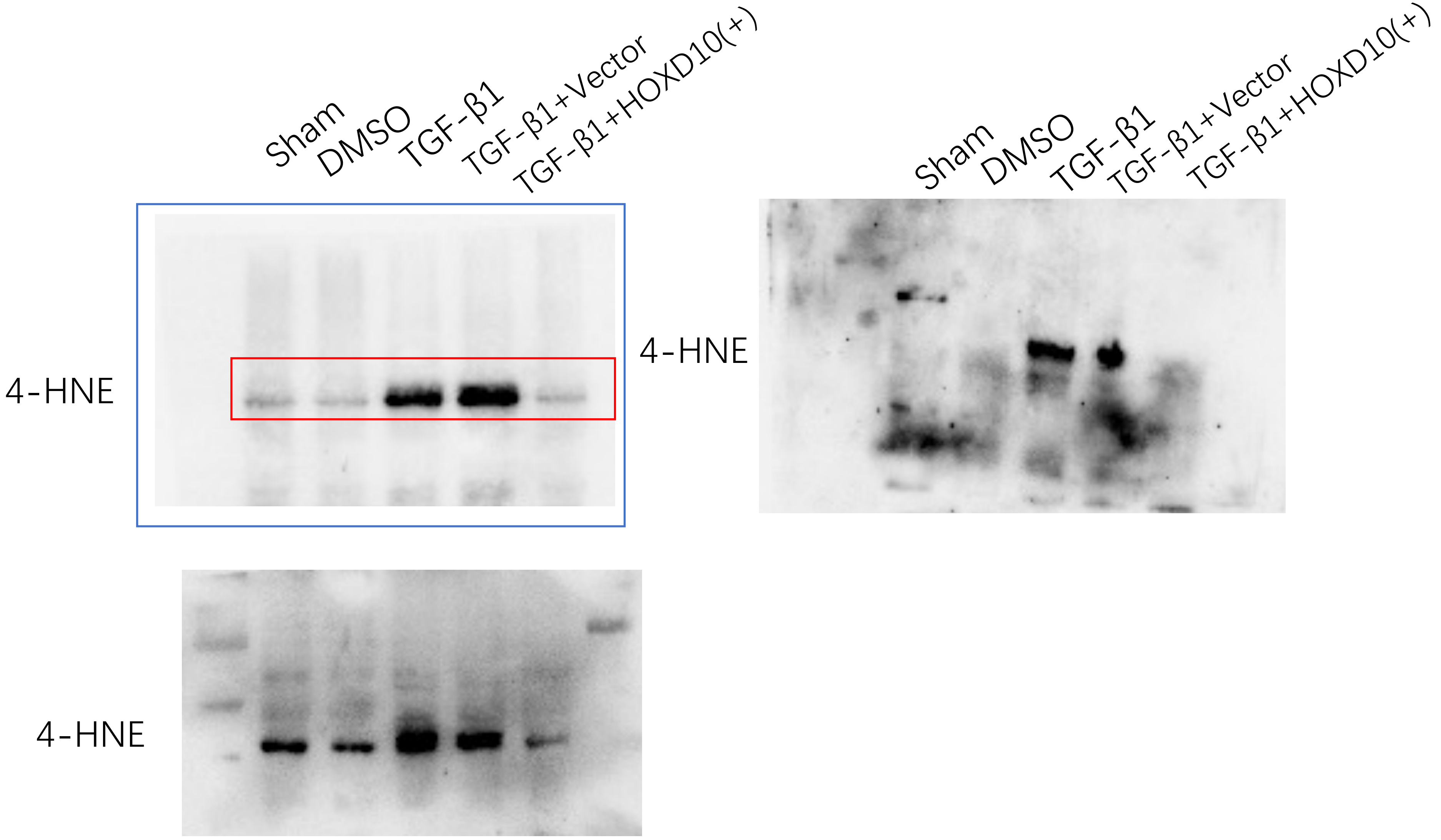

Sham
DMSO
TGF-β1
TGF-β1+Vector
TGF-β1+HOXD10(+)
4-HNE
Sham
DMSO
TGF-β1
TGF-β1+Vector
TGF-β1+HOXD10(+)
4-HNE
4-HNE

Figure 5C

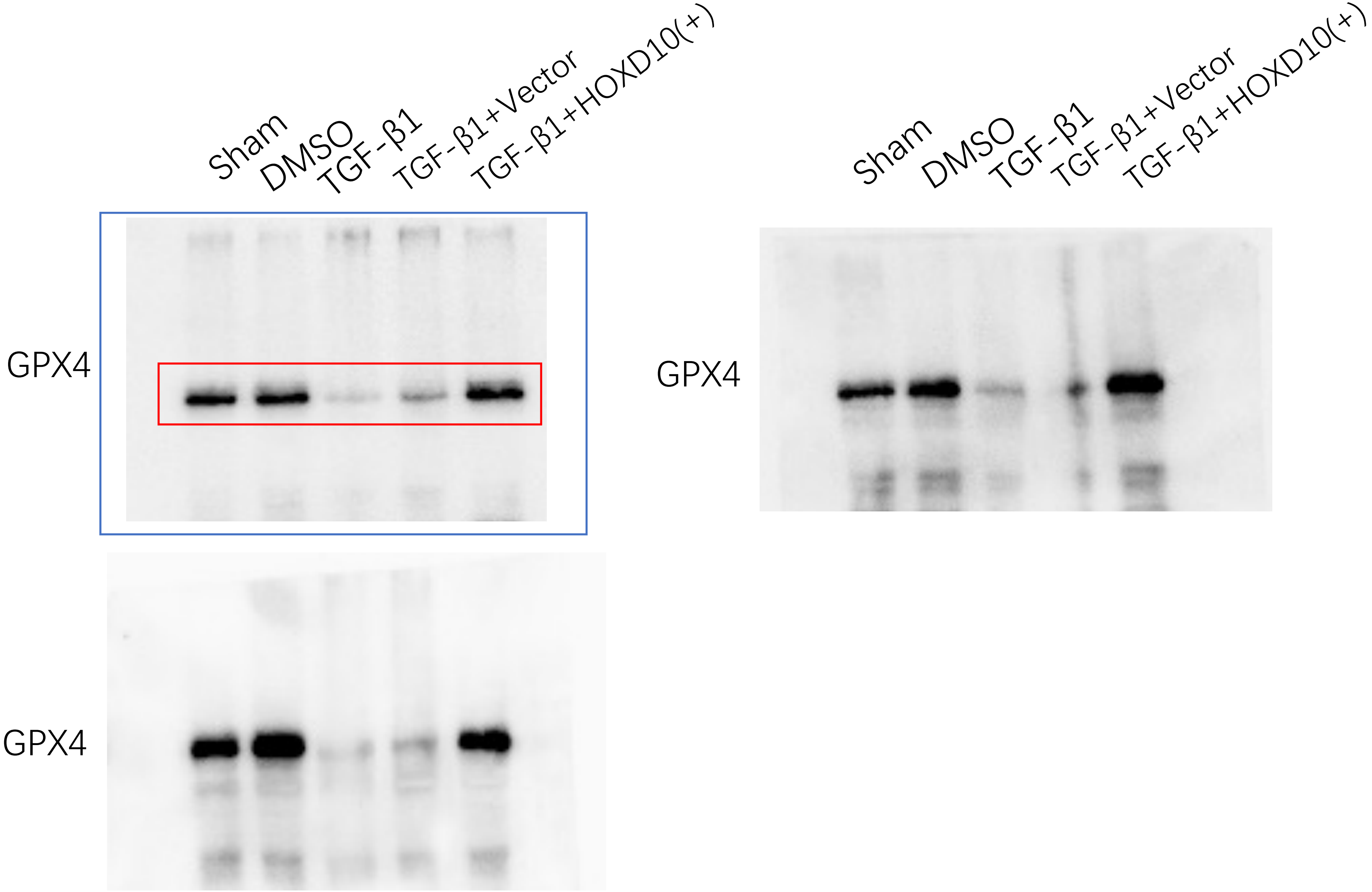
Sham
DMSO
TGF-β1
TGF-β1+Vector
TGF-β1+HOXD10(+)
GPX4
Sham
DMSO
TGF-β1
TGF-β1+Vector
TGF-β1+HOXD10(+)
GPX4
GPX4

Figure 5C

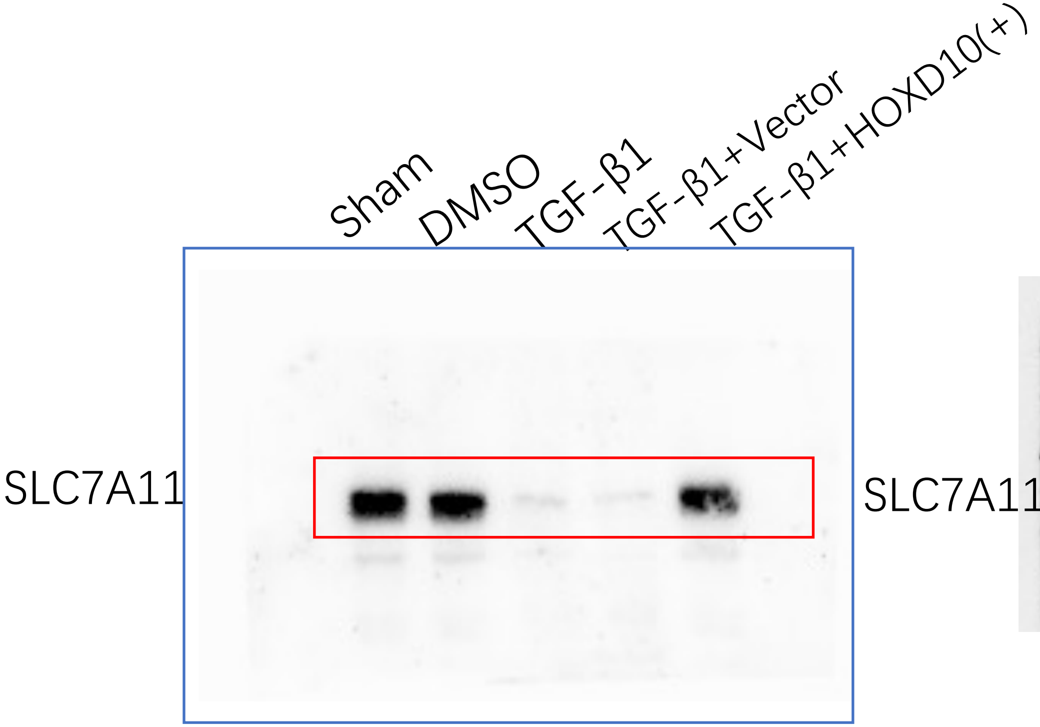

Sham
DMSO
TGF-β1
TGF-β1+Vector
TGF-β1+HOXD10(+)
SLC7A11

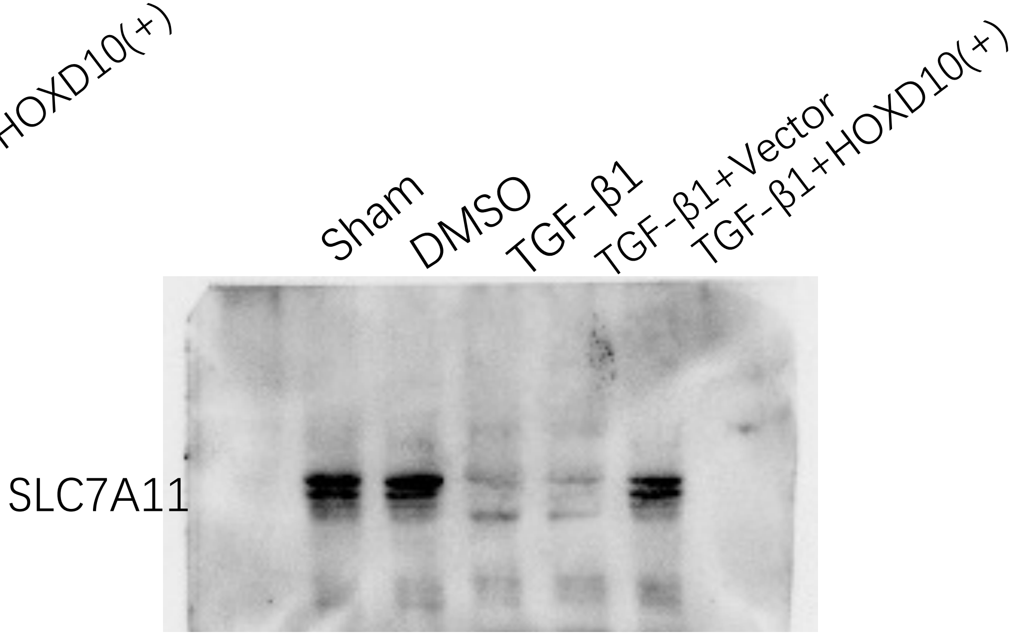

Sham
DMSO
TGF-β1
TGF-β1+Vector
TGF-β1+HOXD10(+)
SLC7A11

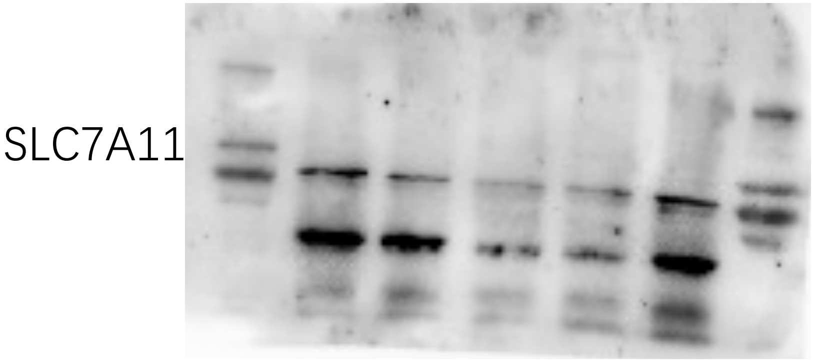

SLC7A11

Figure 5C

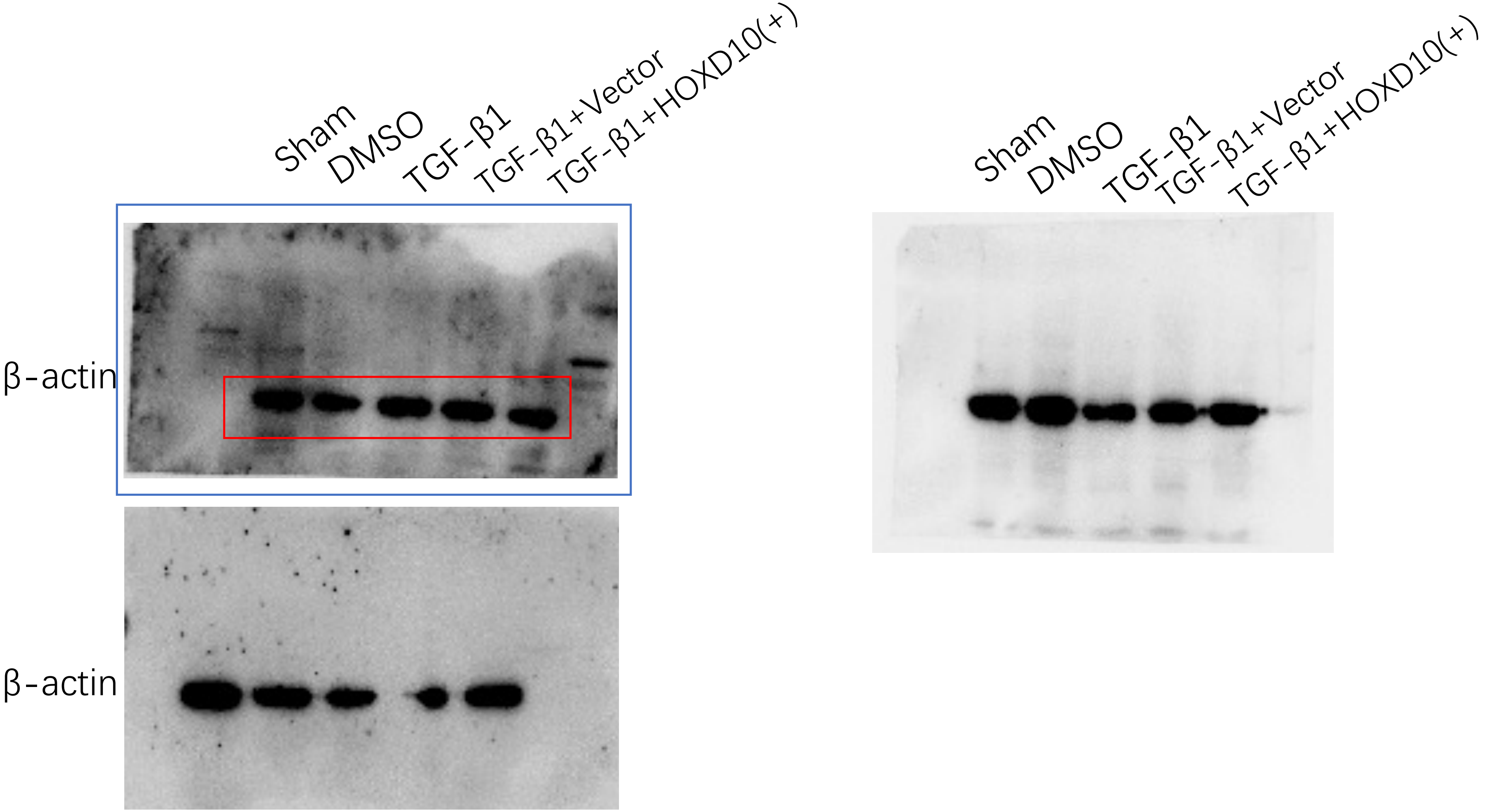

Sham
DMSO
TGF-β1
TGF-β1+Vector
TGF-β1+HOXD10(+)
β-actin
β-actin
Sham
DMSO
TGF-β1
TGF-β1+Vector
TGF-β1+HOXD10(+)

Figure 5D

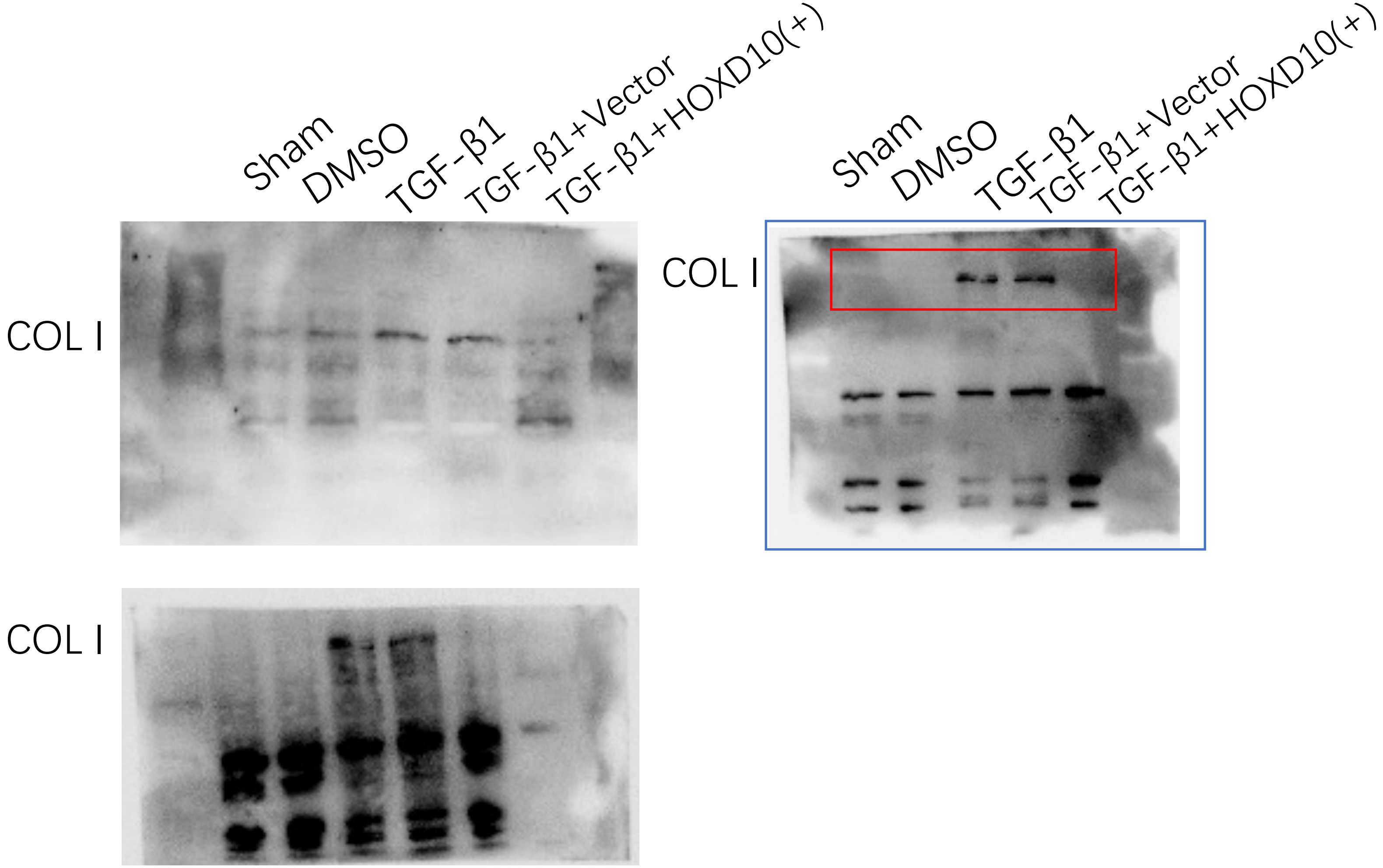

Sham
DMSO
TGF-β1
TGF-β1+Vector
TGF-β1+HOXD10(+)
COL I
COL I
Sham
DMSO
TGF-β1
TGF-β1+Vector
TGF-β1+HOXD10(+)
COL I

Figure 5D

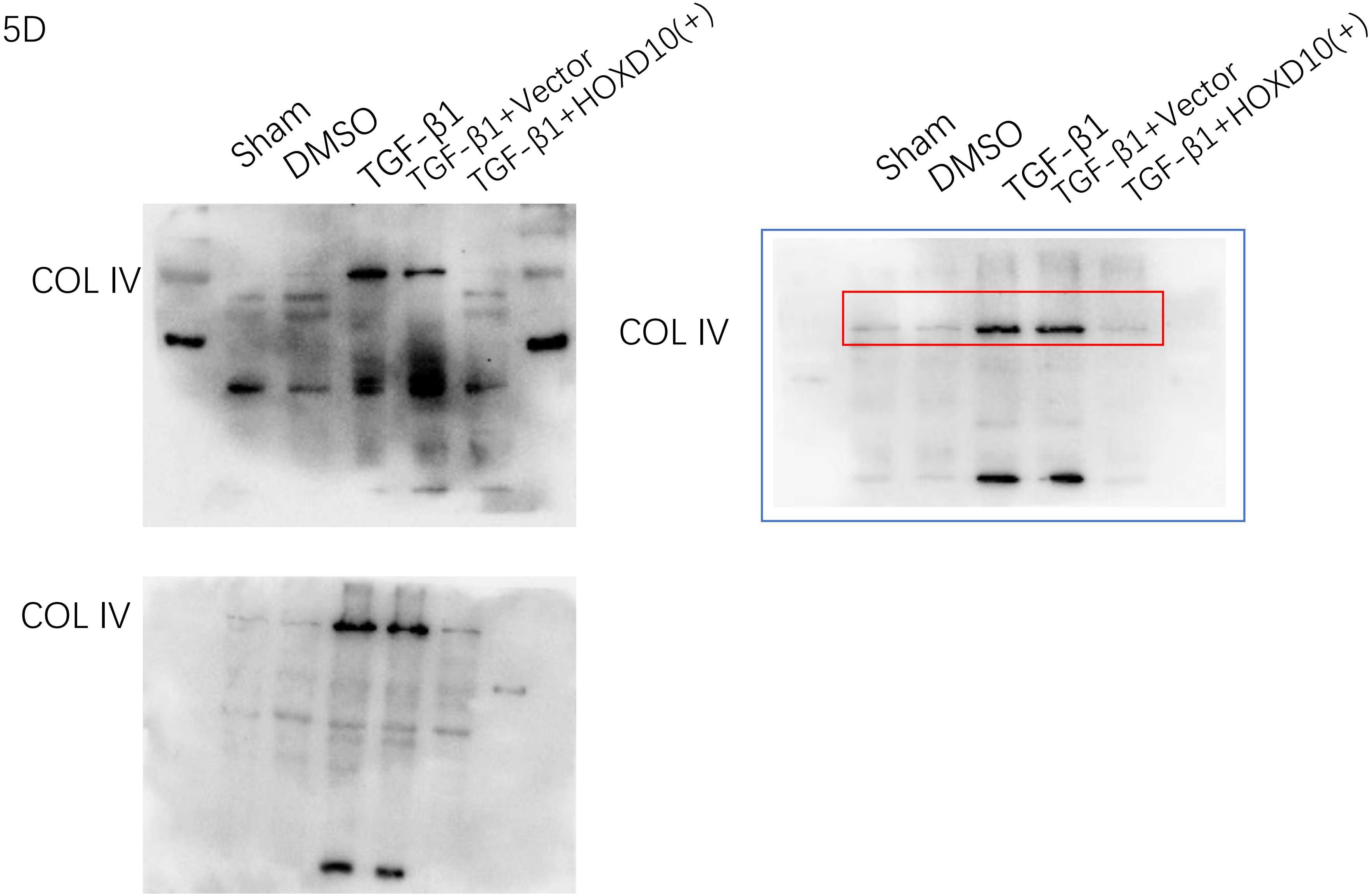
Sham
DMSO
TGF-β1
TGF-β1+Vector
TGF-β1+HOXD10(+)
COL IV
COL IV
Sham
DMSO
TGF-β1
TGF-β1+Vector
TGF-β1+HOXD10(+)
COL IV

Figure 5D

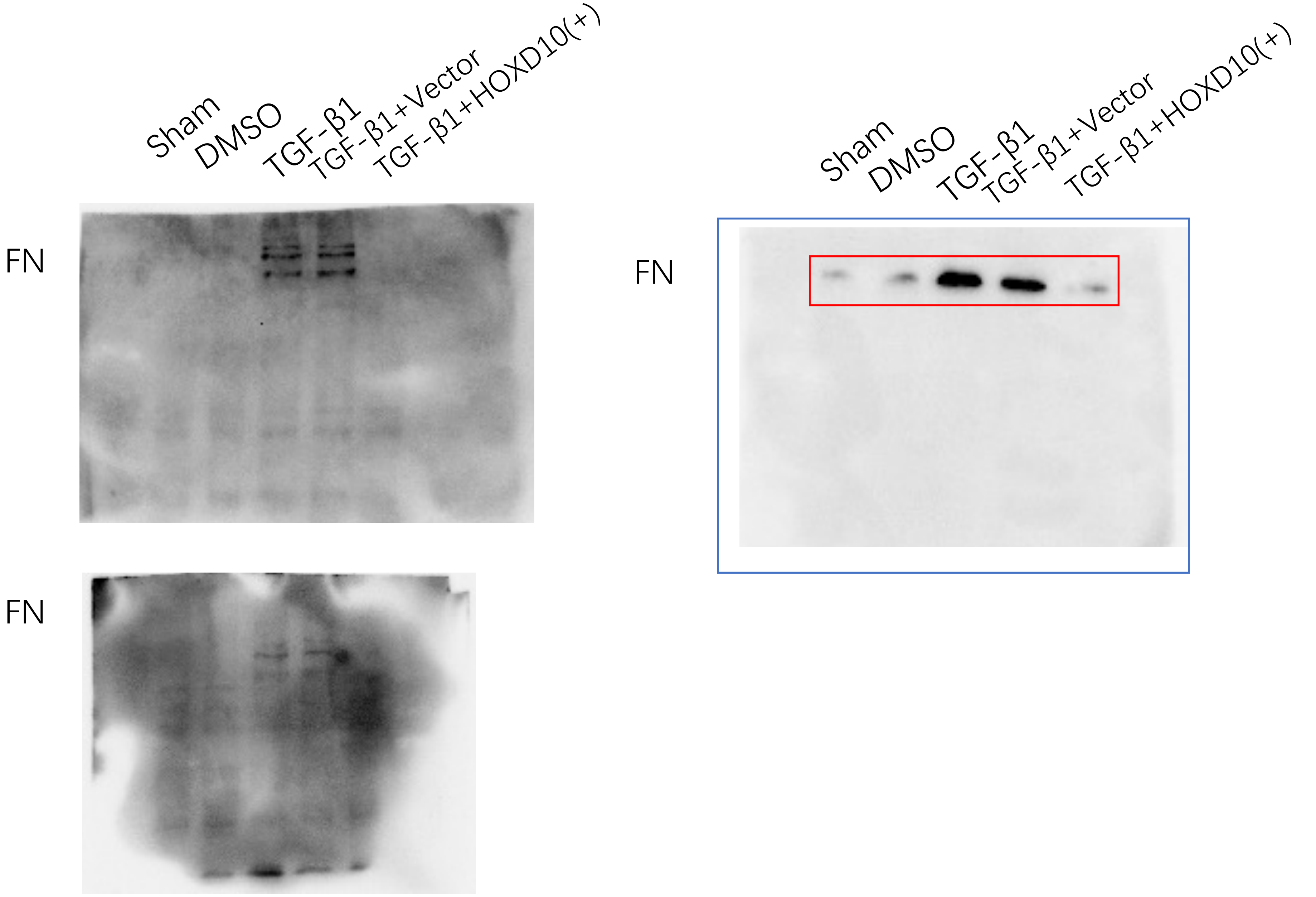
Sham
DMSO
TGF-β1
TGF-β1+Vector
TGF-β1+HOXD10(+)
FN
FN
Sham
DMSO
TGF-β1
TGF-β1+Vector
TGF-β1+HOXD10(+)
FN

Figure 5D

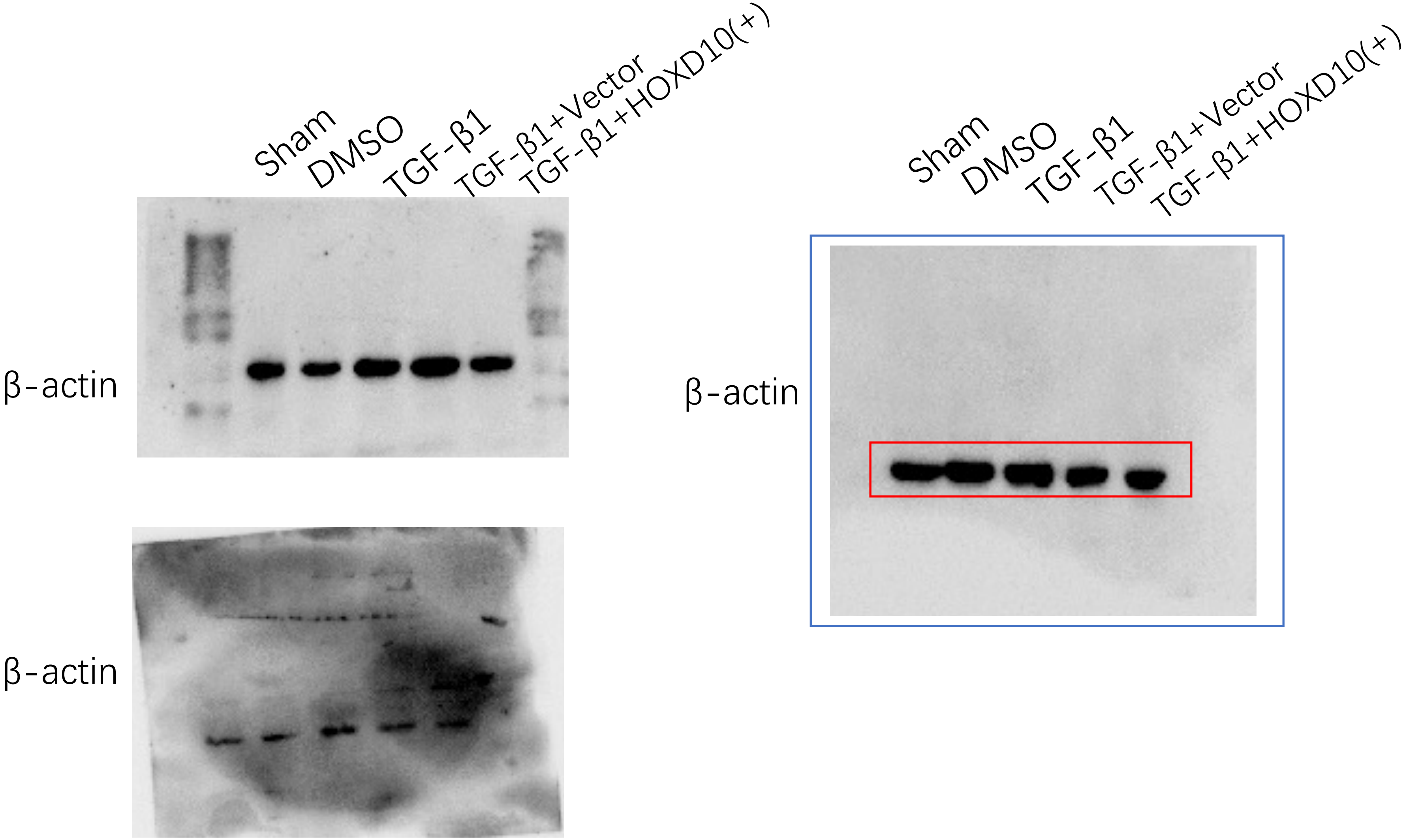
Sham
DMSO
TGF-β1
TGF-β1+Vector
TGF-β1+HOXD10(+)
β-actin
β-actin
Sham
DMSO
TGF-β1
TGF-β1+Vector
TGF-β1+HOXD10(+)
β-actin

Figure 5D

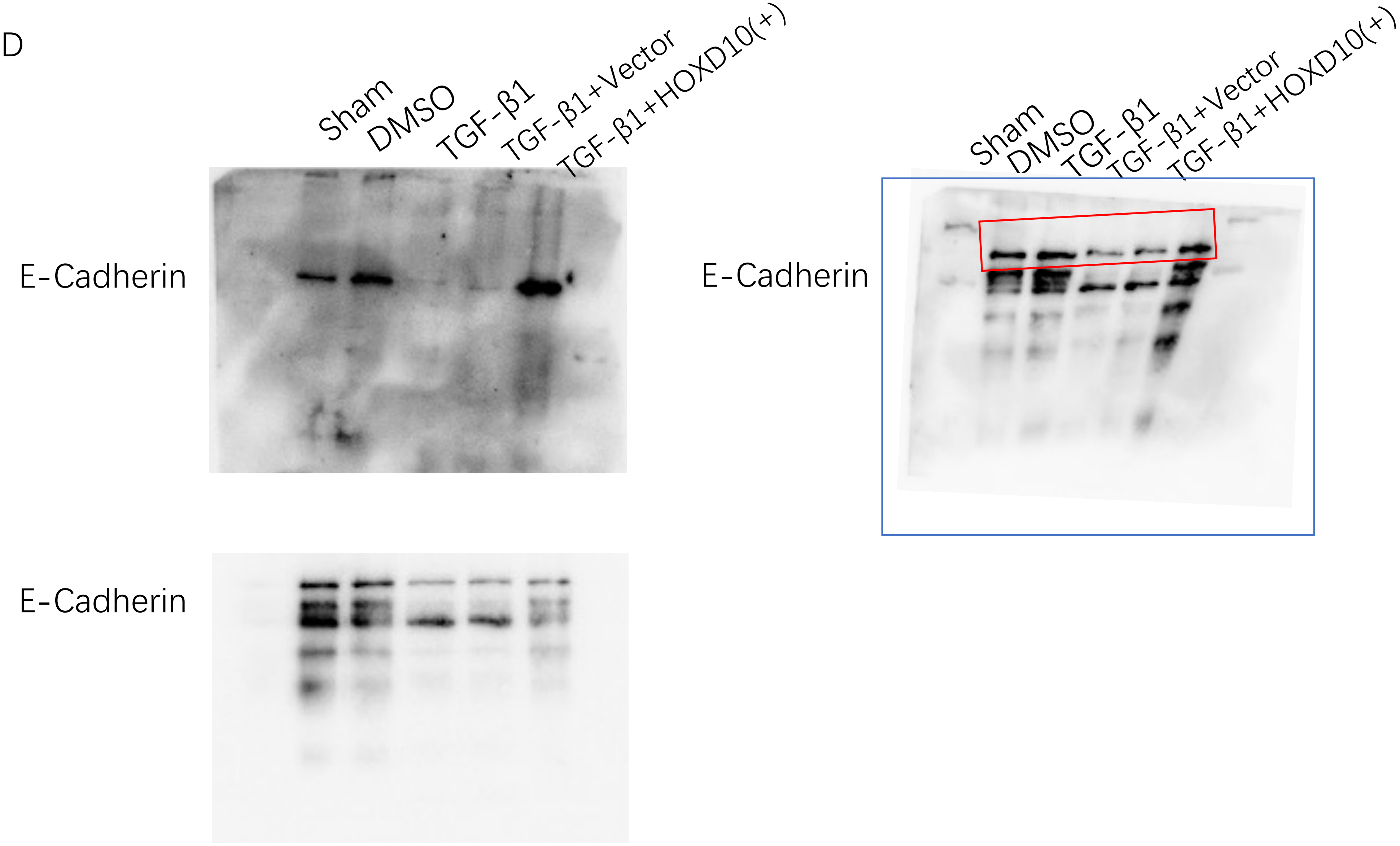
Sham
DMSO
TGF-β1
TGF-β1+Vector
TGF-β1+HOXD10(+)
E-Cadherin
E-Cadherin
Sham
DMSO
TGF-β1
TGF-β1+Vector
TGF-β1+HOXD10(+)
E-Cadherin

Figure 5D

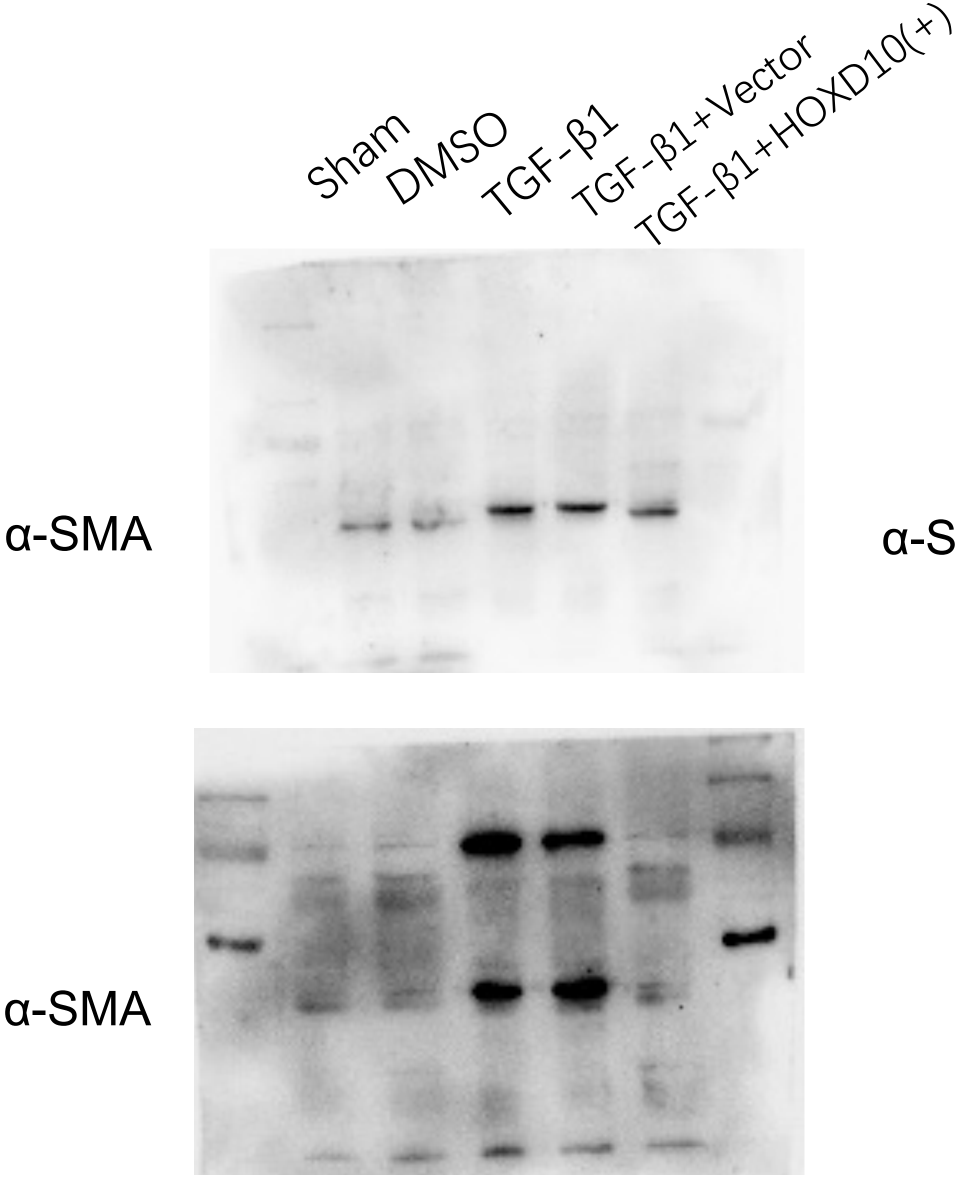
Sham
DMSO
TGF-β1
TGF-β1+Vector
TGF-β1+HOXD10(+)
α-SMA
α-SMA

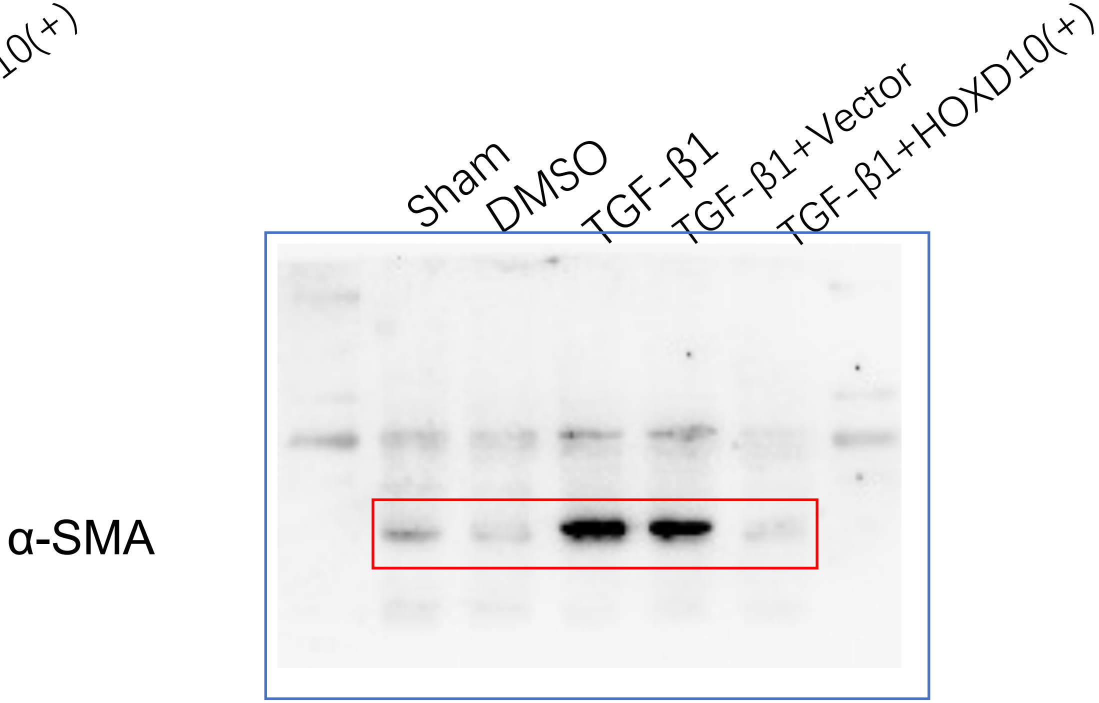
Sham
DMSO
TGF-β1
TGF-β1+Vector
TGF-β1+HOXD10(+)
α-SMA

Figure 5D

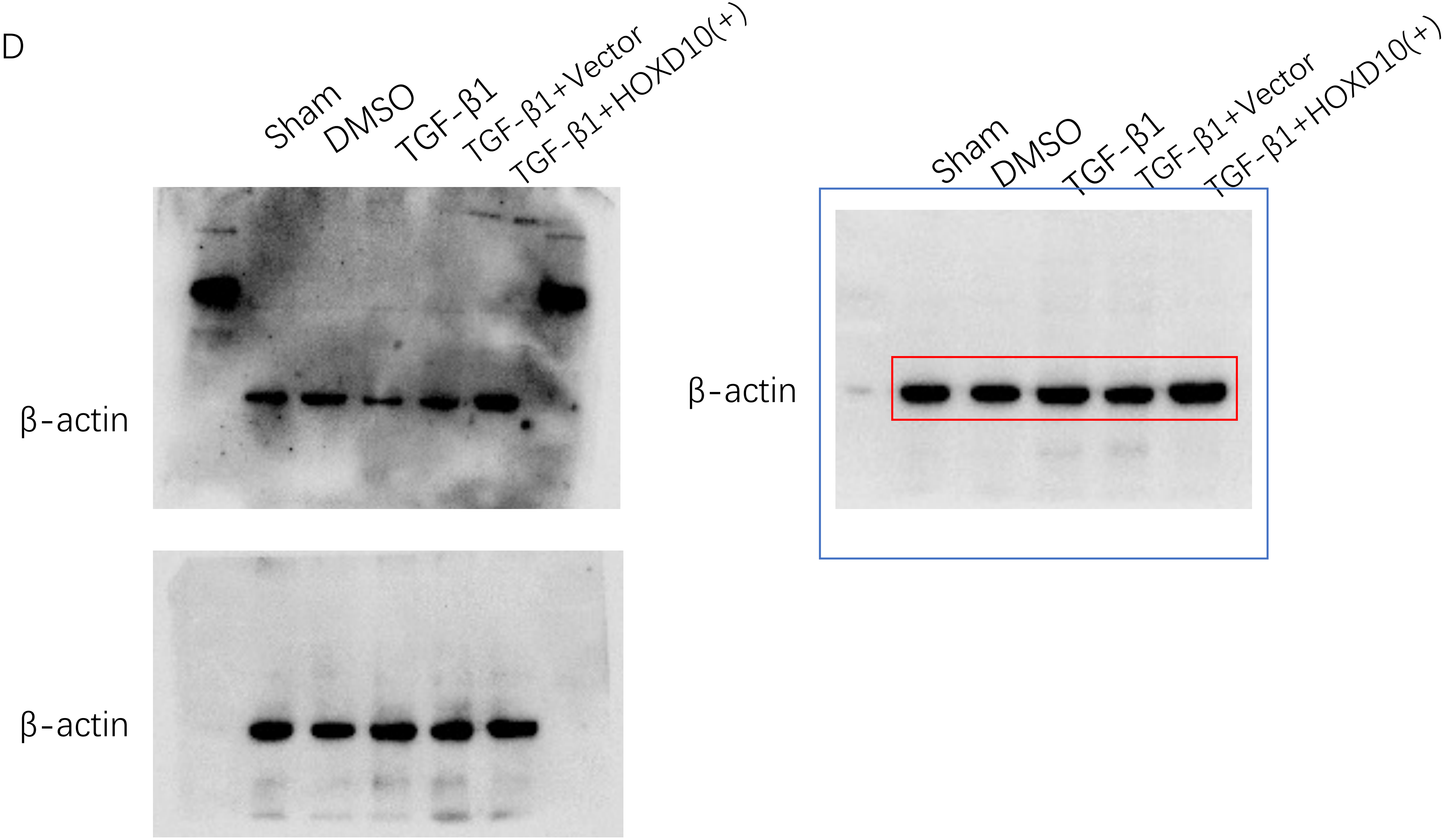
Sham
DMSO
TGF-β1
TGF-β1+Vector
TGF-β1+HOXD10(+)
β-actin
β-actin
Sham
DMSO
TGF-β1
TGF-β1+Vector
TGF-β1+HOXD10(+)
β-actin

Figure 6D

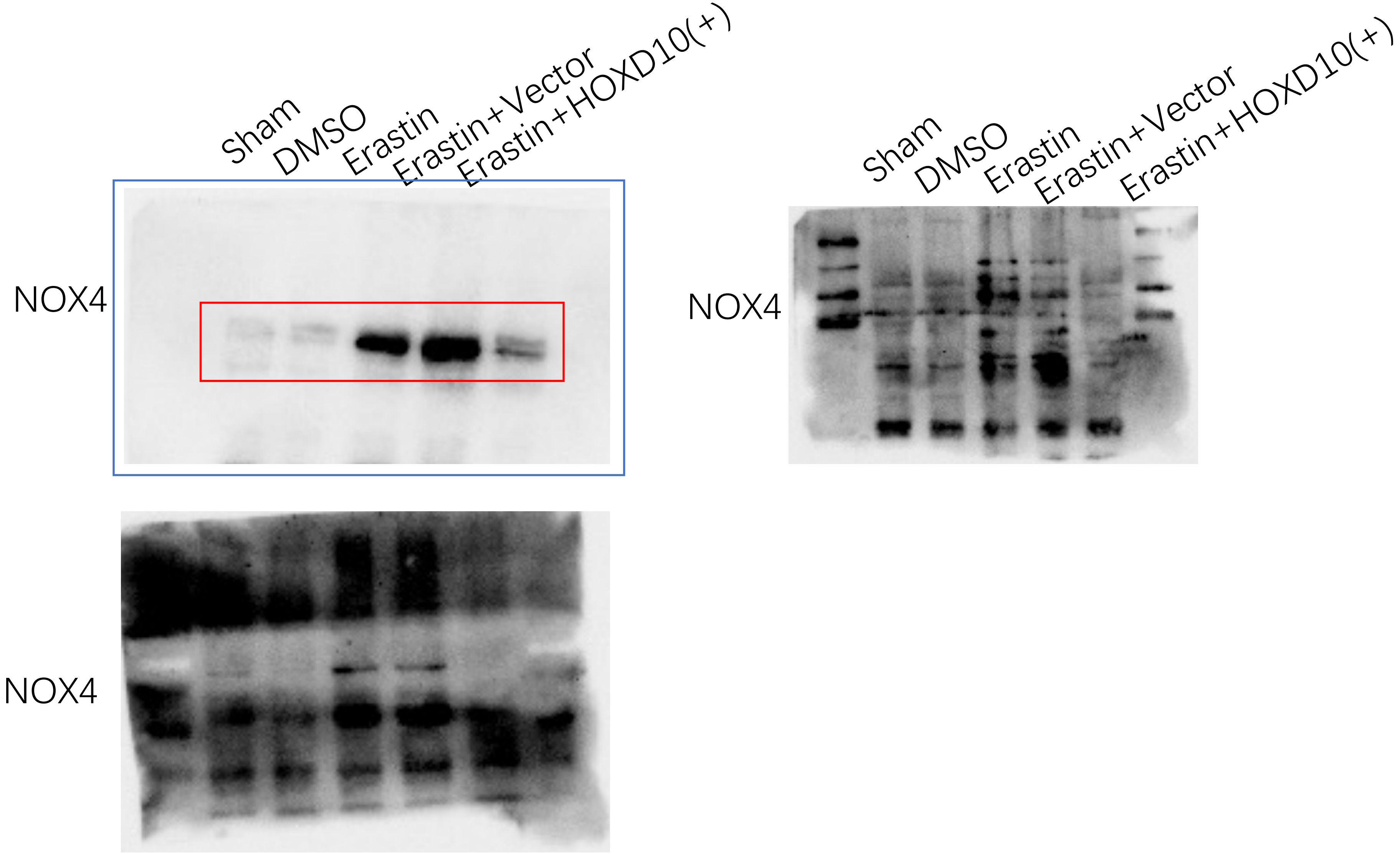
Sham
DMSO
Erastin
Erastin+Vector
Erastin+HOXD10(+)
NOX4
Sham
DMSO
Erastin
Erastin+Vector
Erastin+HOXD10(+)
NOX4
NOX4

Figure 6D

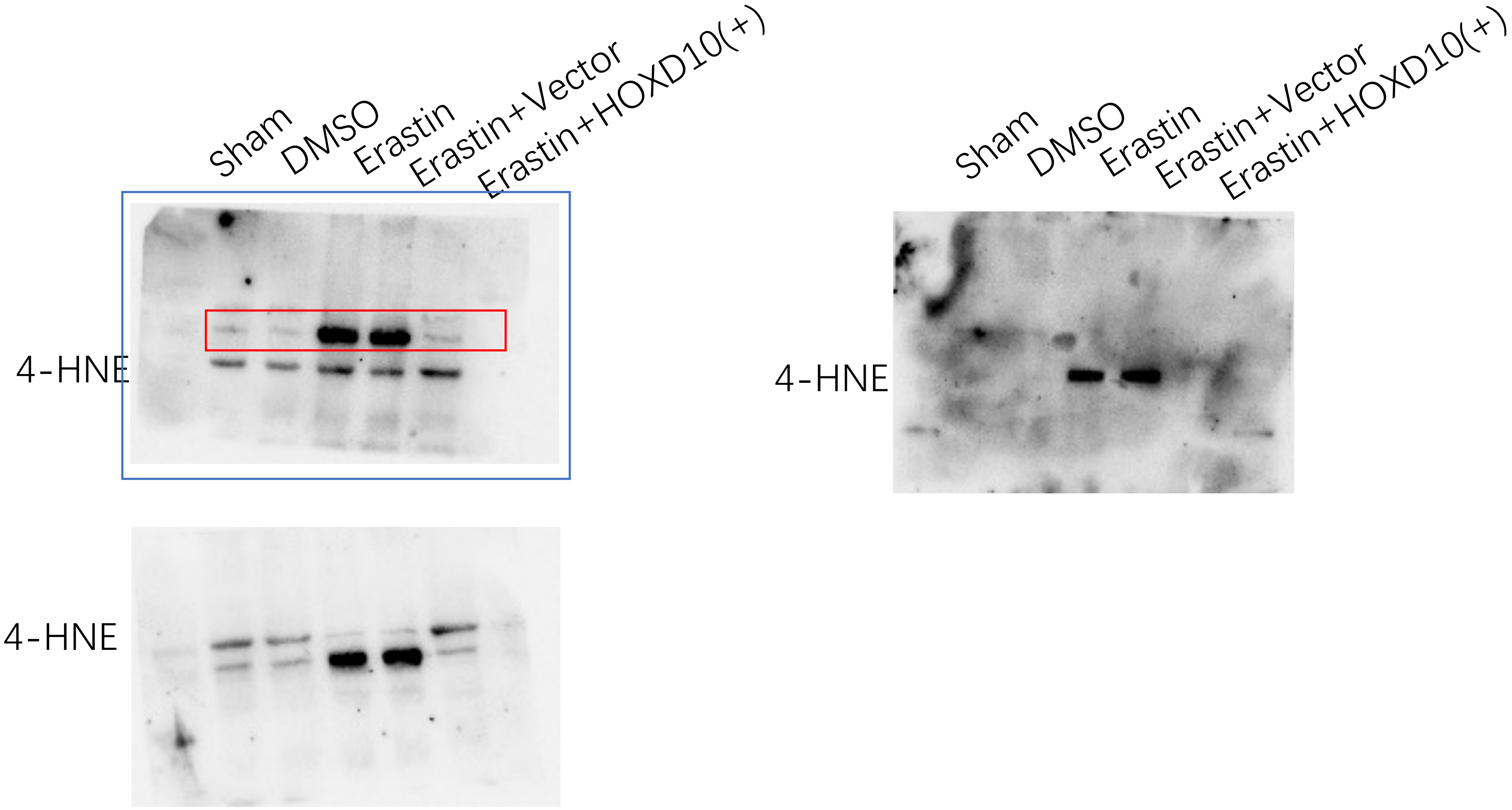
Sham
DMSO
Erastin
Erastin+Vector
Erastin+HOXD10(+)
4-HNE
4-HNE
Sham
DMSO
Erastin
Erastin+Vector
Erastin+HOXD10(+)
4-HNE

Figure 6D

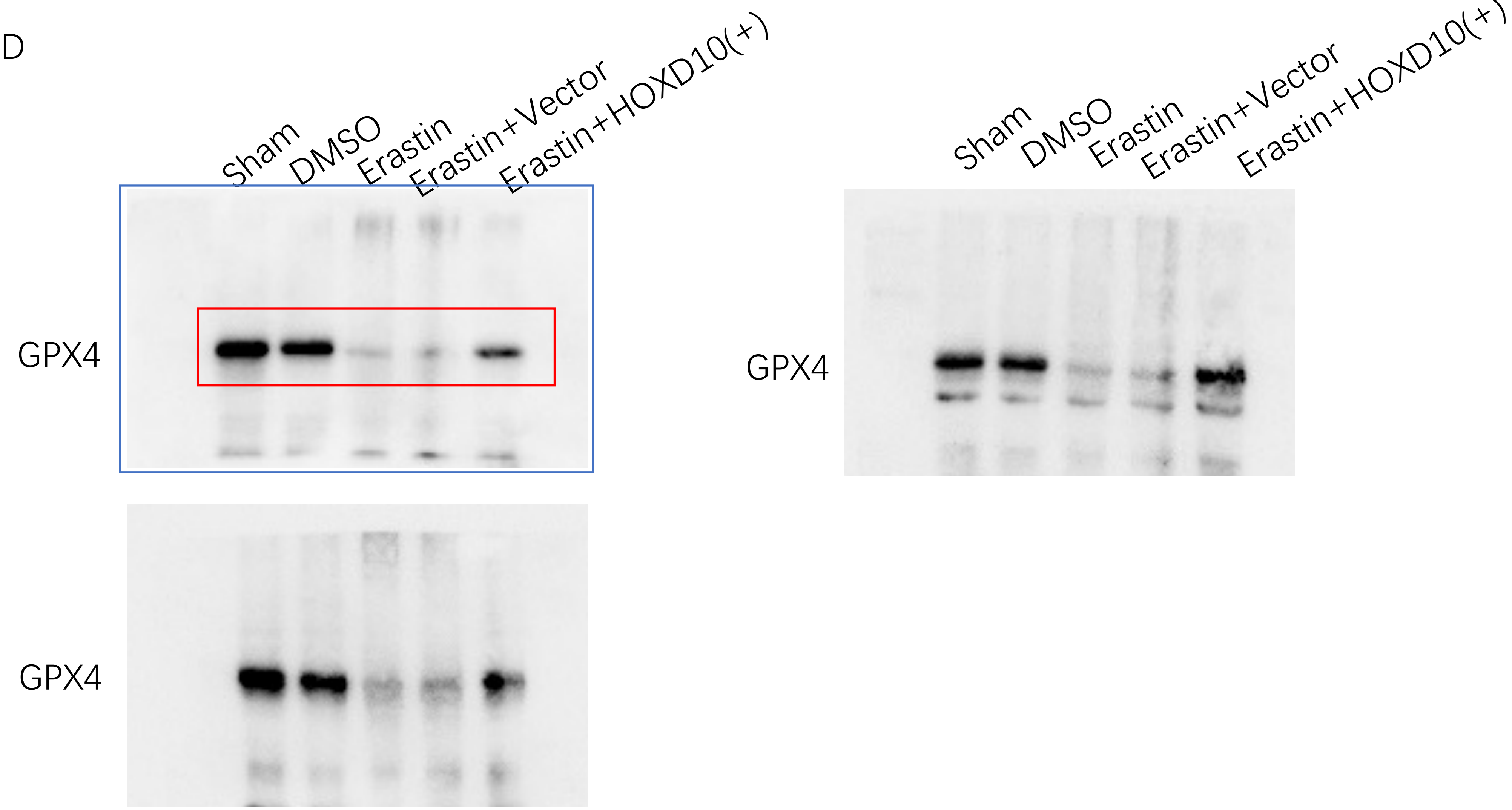
Sham
DMSO
Erastin
Erastin+Vector
Erastin+HOXD10(+)
GPX4
GPX4
Sham
DMSO
Erastin
Erastin+Vector
Erastin+HOXD10(+)
GPX4

Figure 6D

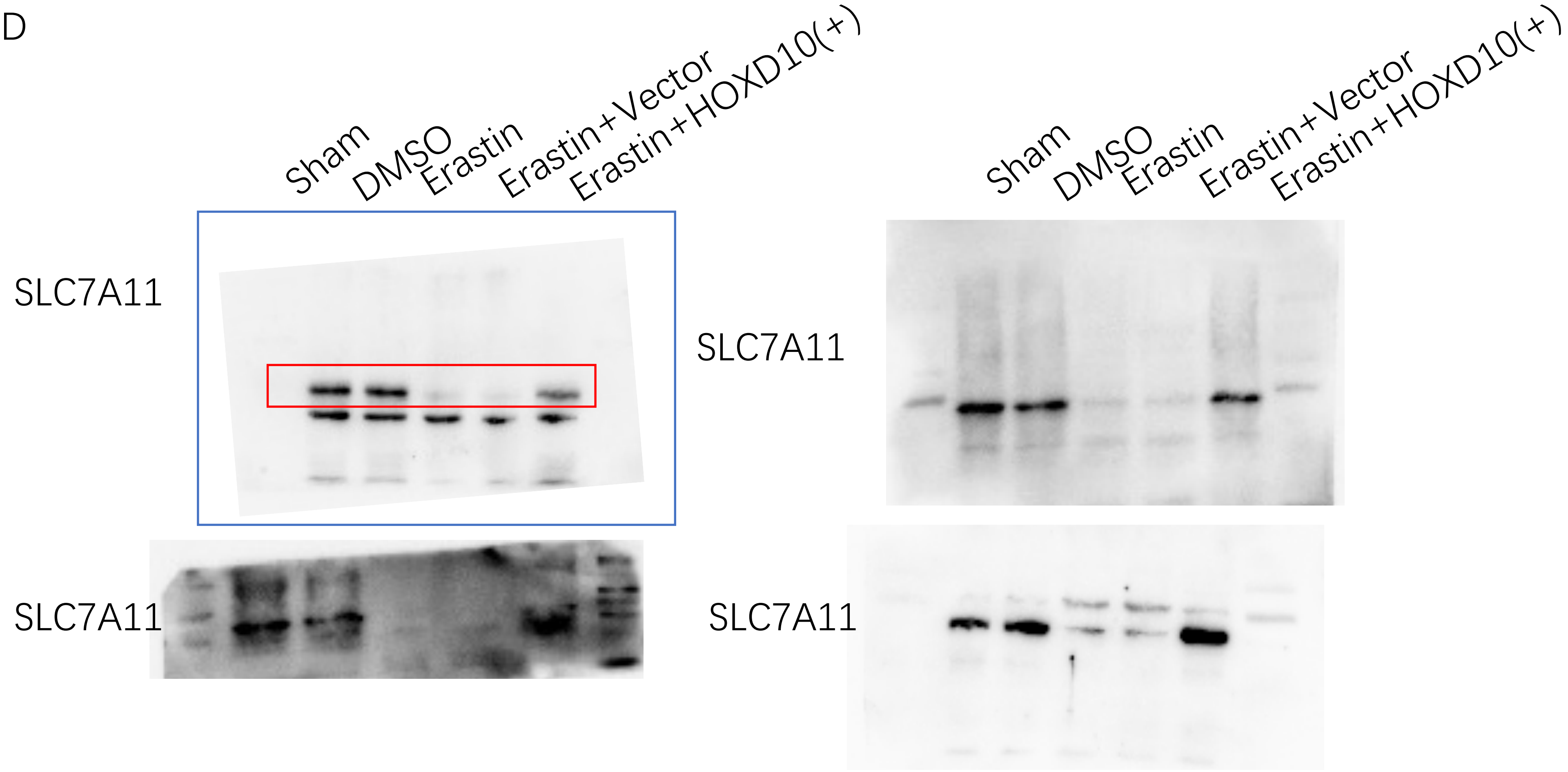
Sham
DMSO
Erastin
Erastin+Vector
Erastin+HOXD10(+)
SLC7A11
SLC7A11
Sham
DMSO
Erastin
Erastin+Vector
Erastin+HOXD10(+)
SLC7A11
SLC7A11

Figure 6D

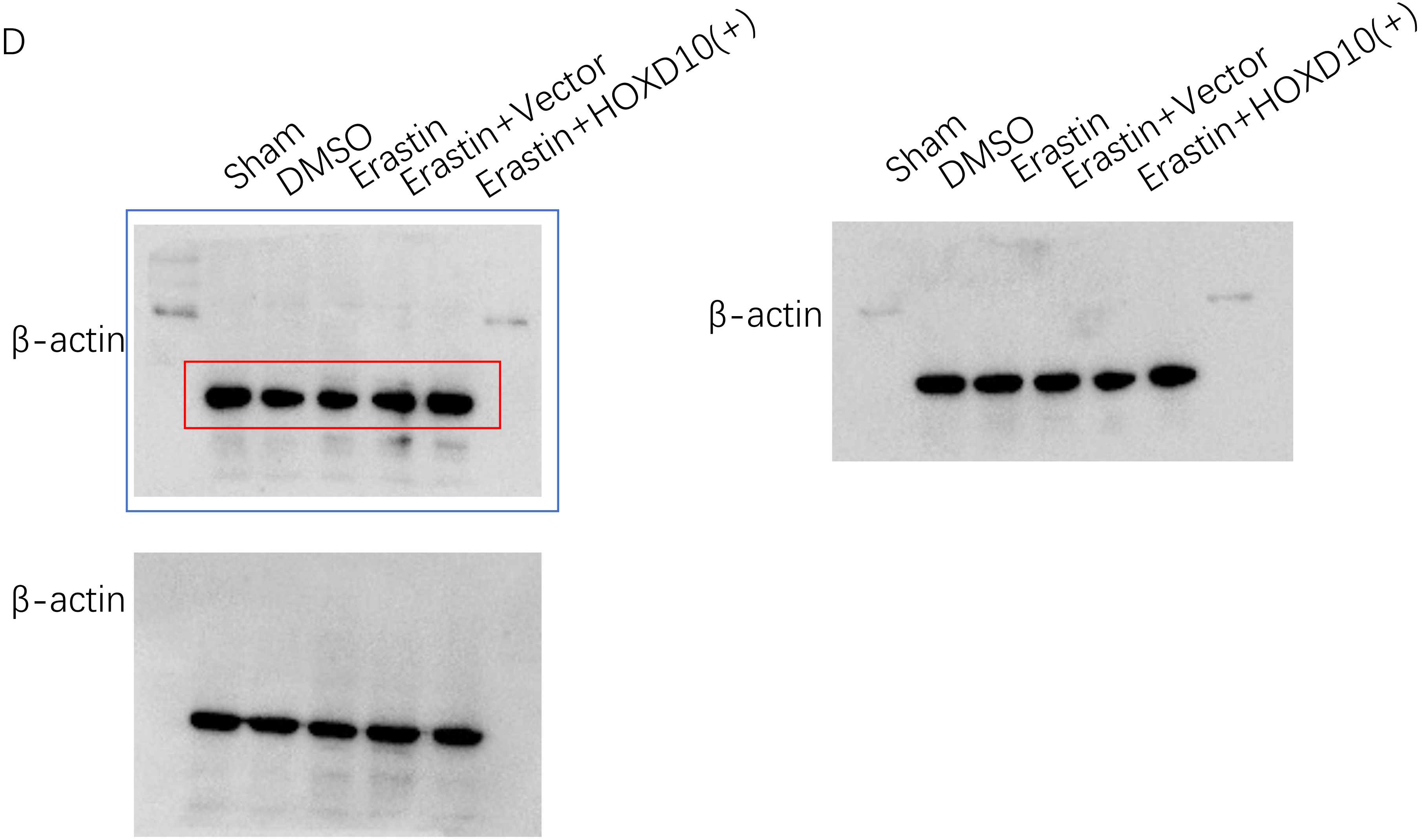

Sham
DMSO
Erastin
Erastin+Vector
Erastin+HOXD10(+)
β-actin
β-actin
Sham
DMSO
Erastin
Erastin+Vector
Erastin+HOXD10(+)
β-actin

Figure 6E

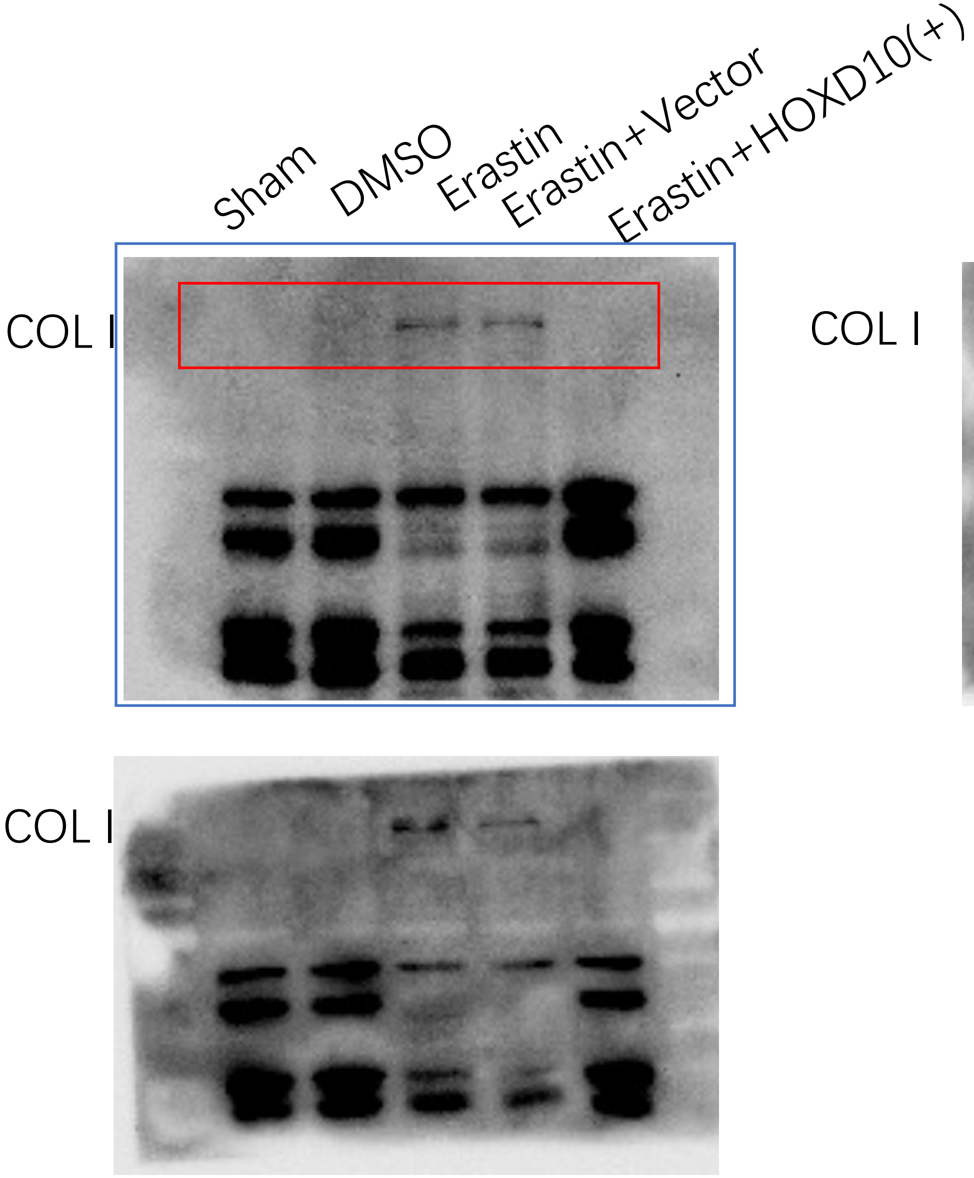

Sham
DMSO
Erastin
Erastin+Vector
Erastin+HOXD10(+)
COL I
COL I

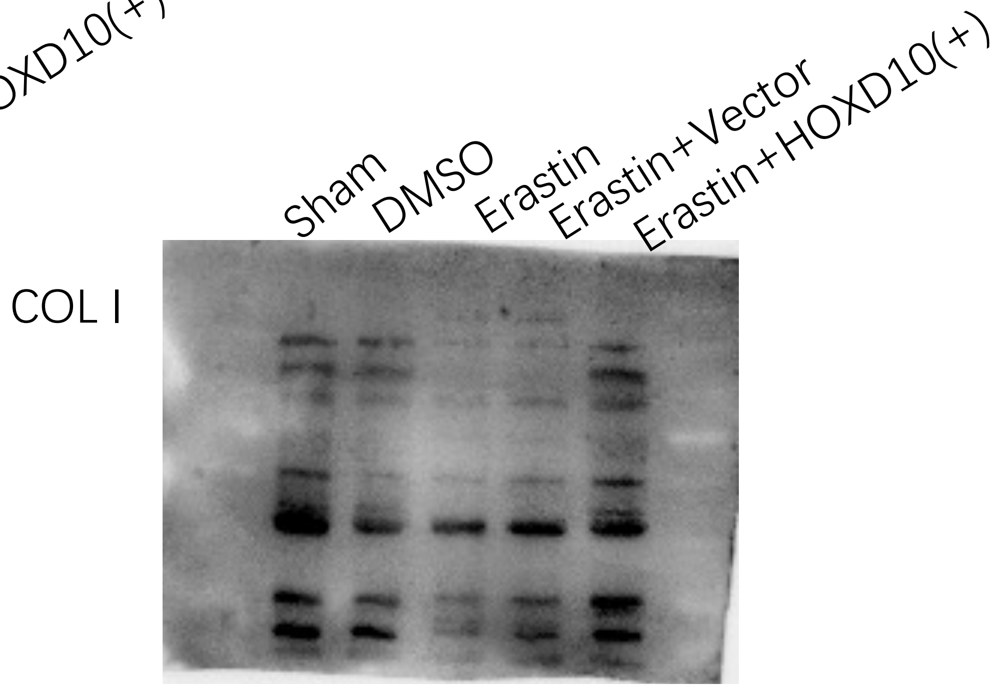

Sham
DMSO
Erastin
Erastin+Vector
Erastin+HOXD10(+)
COL I

Figure 6E

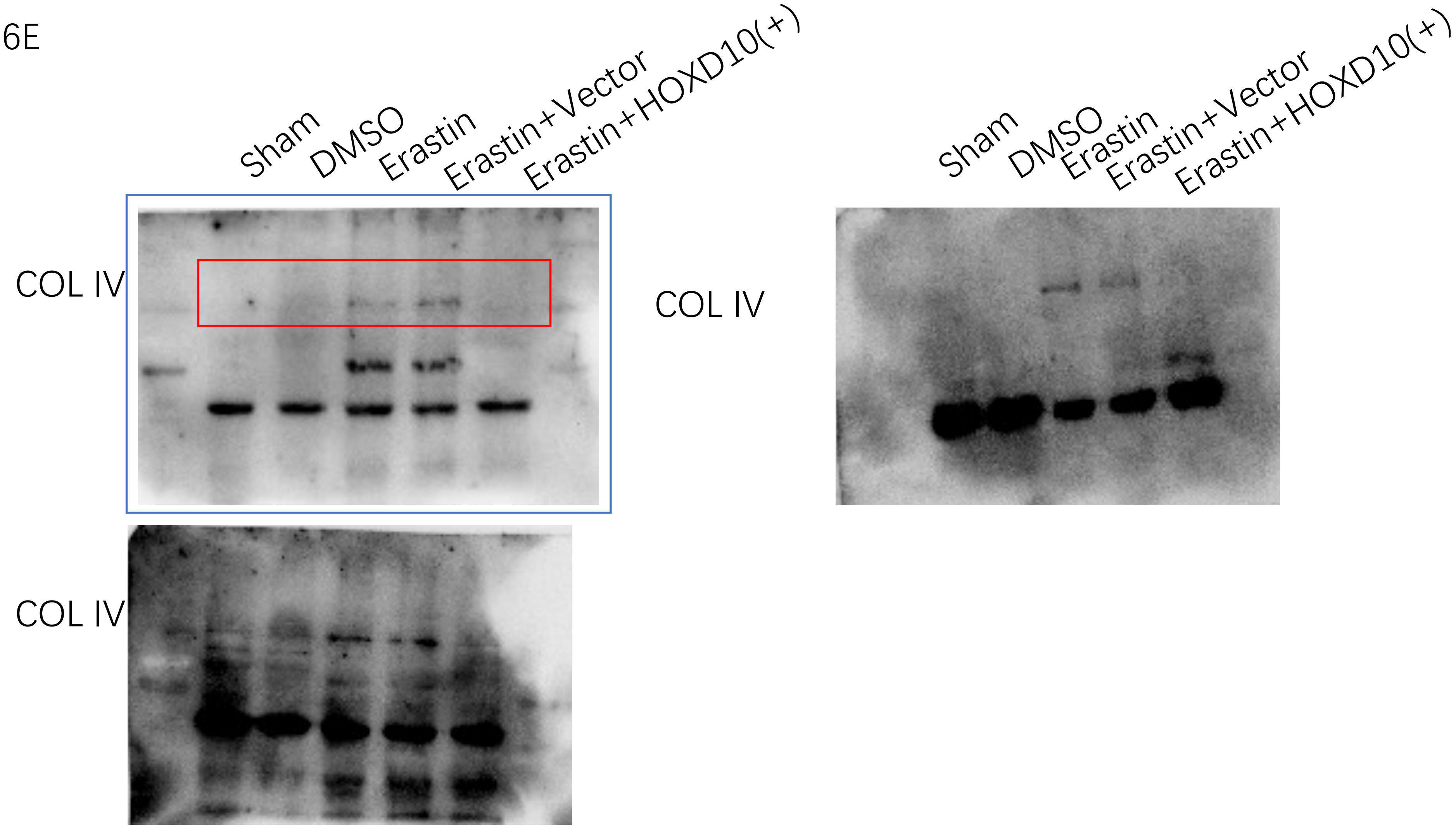
Sham
DMSO
Erastin
Erastin+Vector
Erastin+HOXD10(+)
COL IV
Sham
DMSO
Erastin
Erastin+Vector
Erastin+HOXD10(+)
COL IV
COL IV

Figure 6E

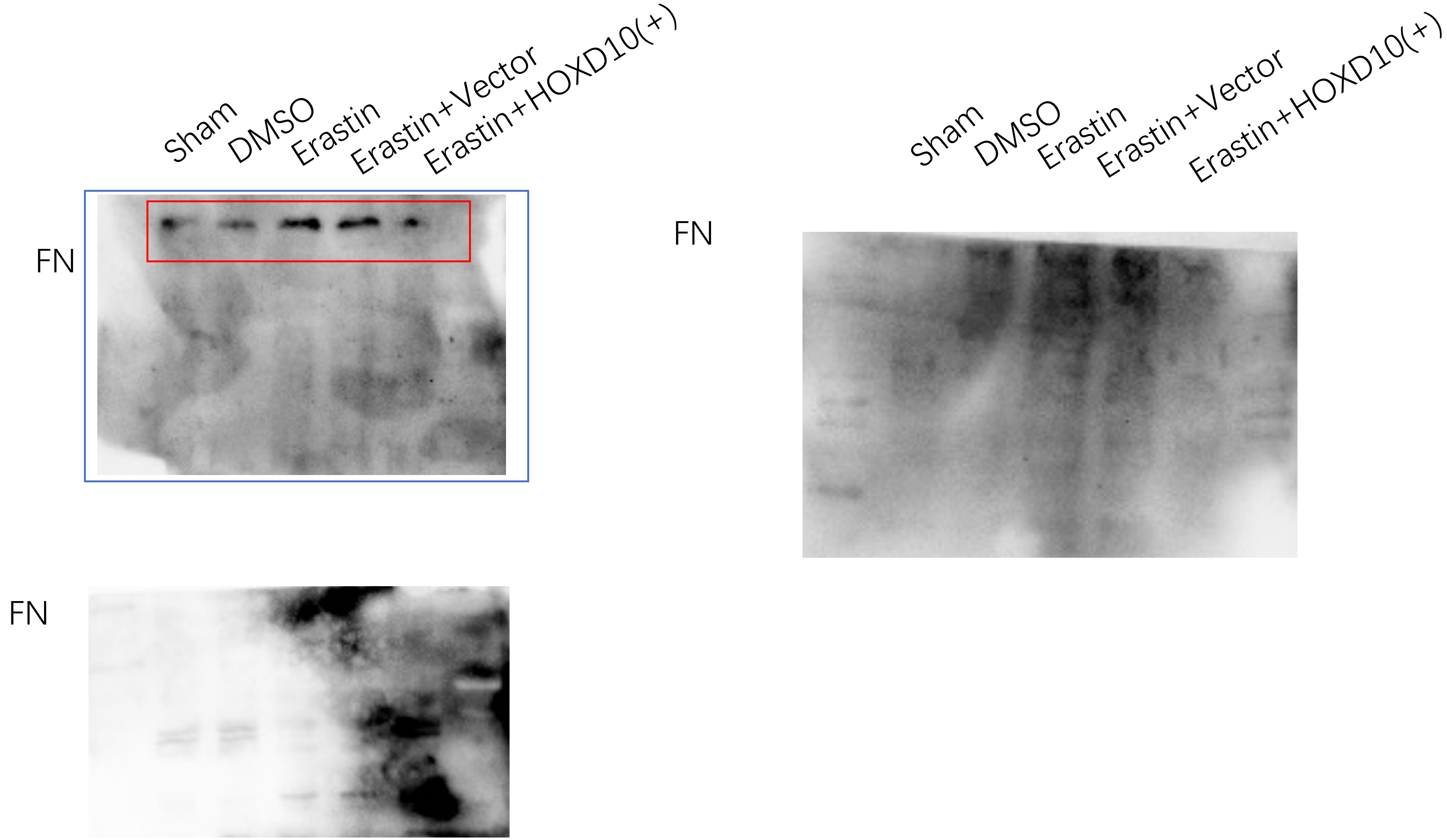
Sham
DMSO
Erastin
Erastin+Vector
Erastin+HOXD10(+)
FN
FN
Sham
DMSO
Erastin
Erastin+Vector
Erastin+HOXD10(+)
FN

Figure 6E

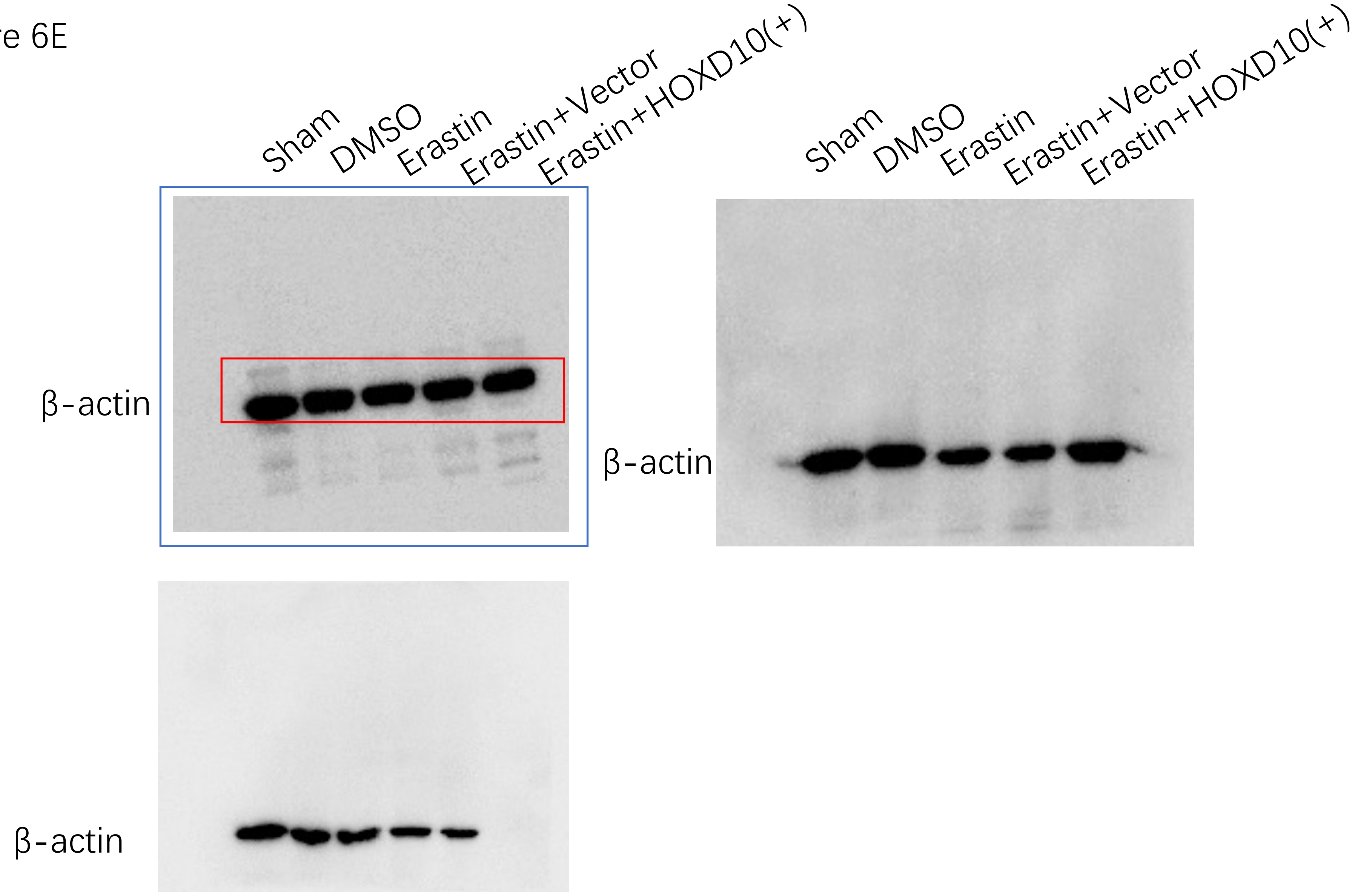
Sham
DMSO
Erastin
Erastin+Vector
Erastin+HOXD10(+)
β-actin
Sham
DMSO
Erastin
Erastin+Vector
Erastin+HOXD10(+)
β-actin
β-actin

Figure 6E

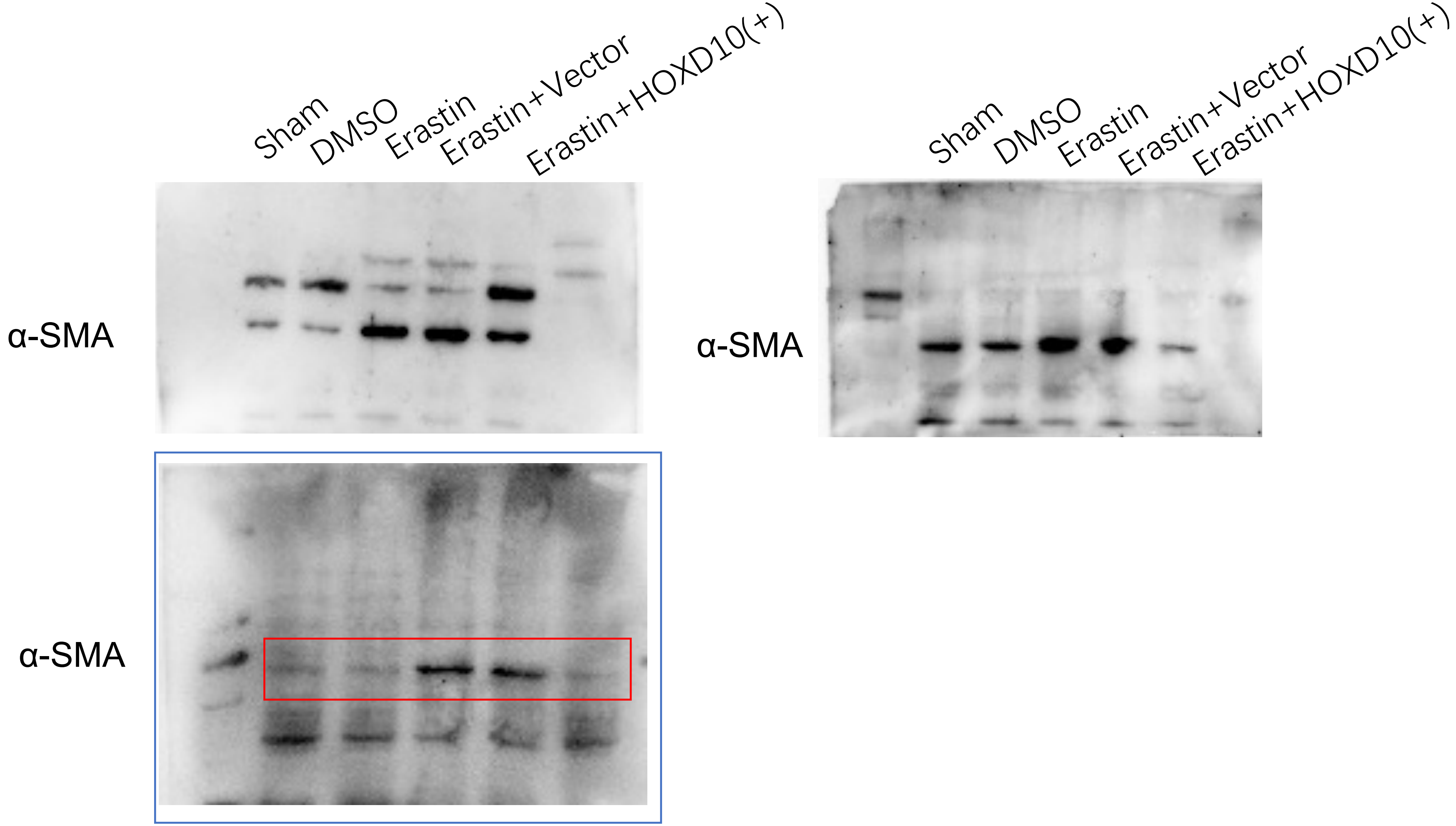

Sham
DMSO
Erastin
Erastin+Vector
Erastin+HOXD10(+)
α-SMA
α-SMA
Sham
DMSO
Erastin
Erastin+Vector
Erastin+HOXD10(+)
α-SMA

Figure 6E

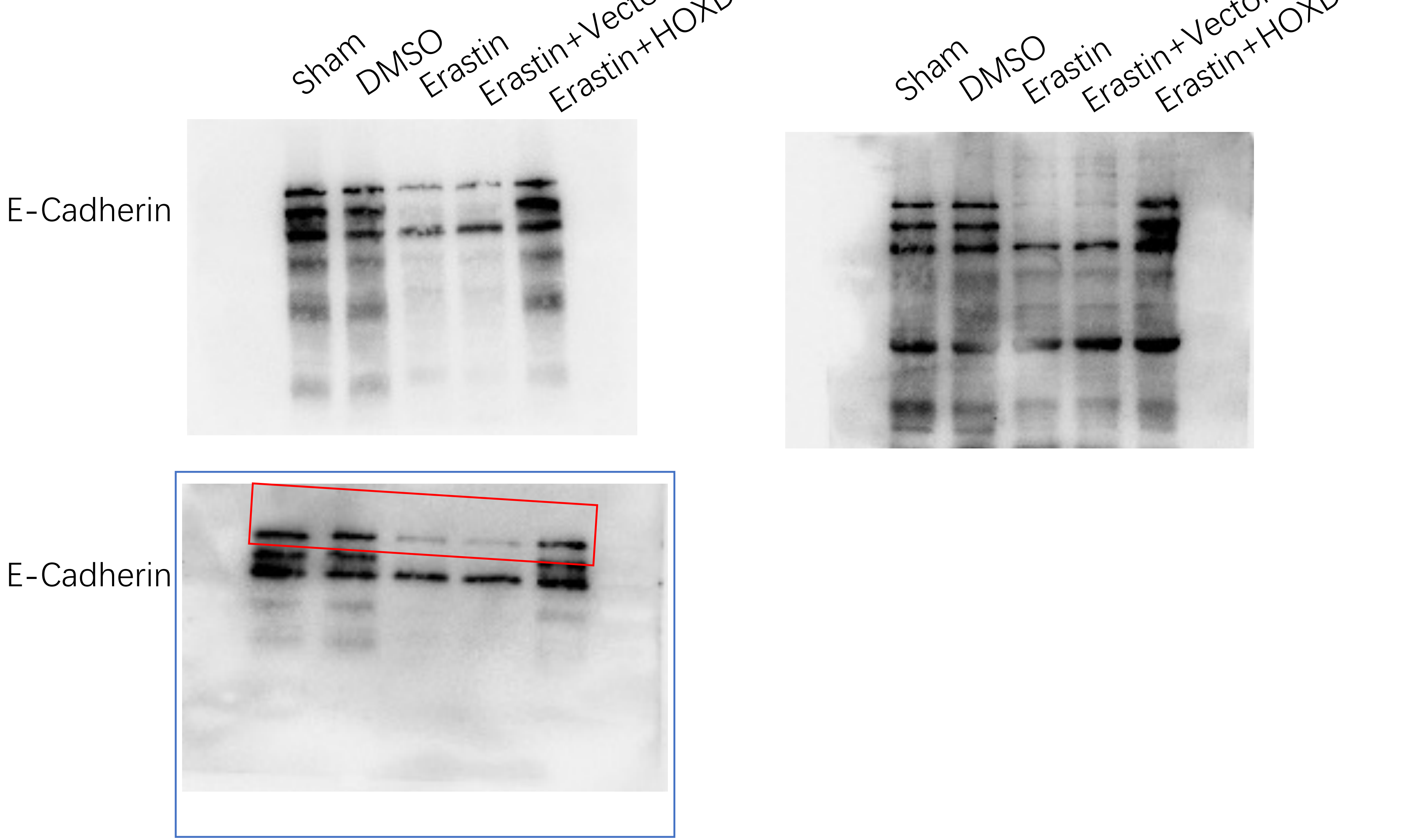

Sham
DMSO
Erastin
Erastin+Vector
Erastin+HOXD10(+)
E-Cadherin
Sham
DMSO
Erastin
Erastin+Vector
Erastin+HOXD10(+)
E-Cadherin

Figure 6E

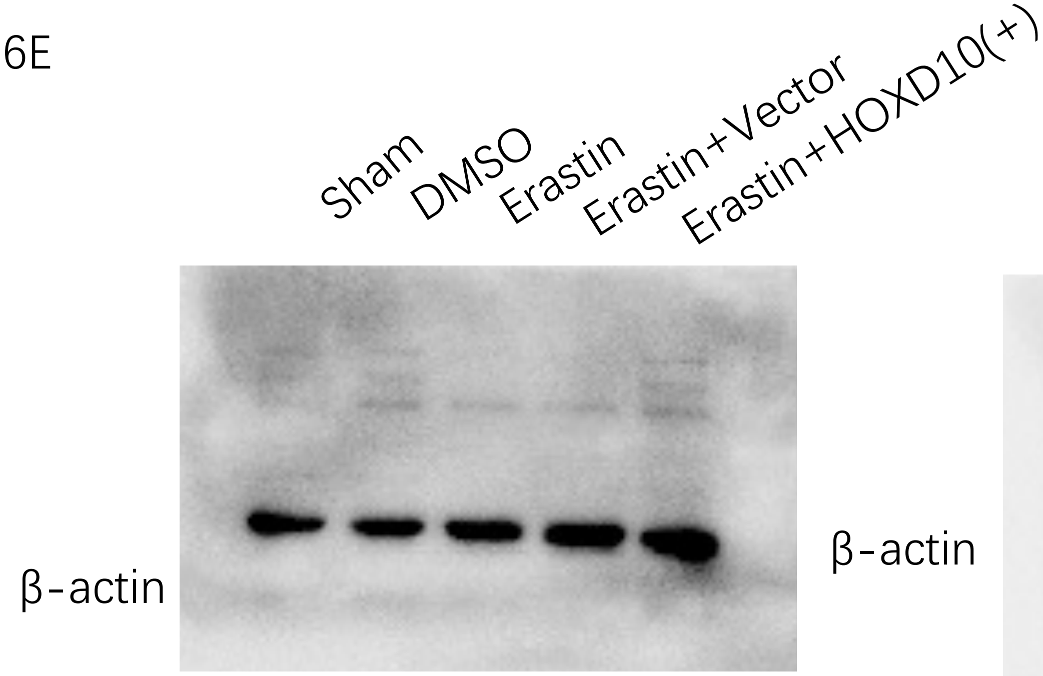

Sham
DMSO
Erastin
Erastin+Vector
Erastin+HOXD10(+)
β-actin

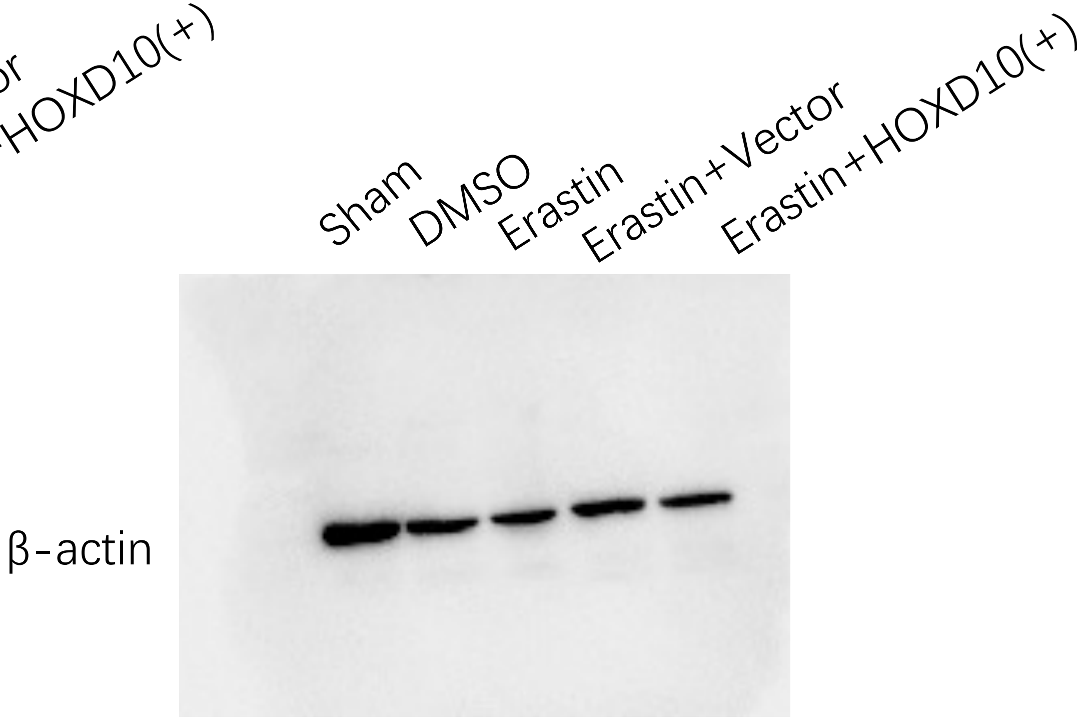

Sham
DMSO
Erastin
Erastin+Vector
Erastin+HOXD10(+)
β-actin

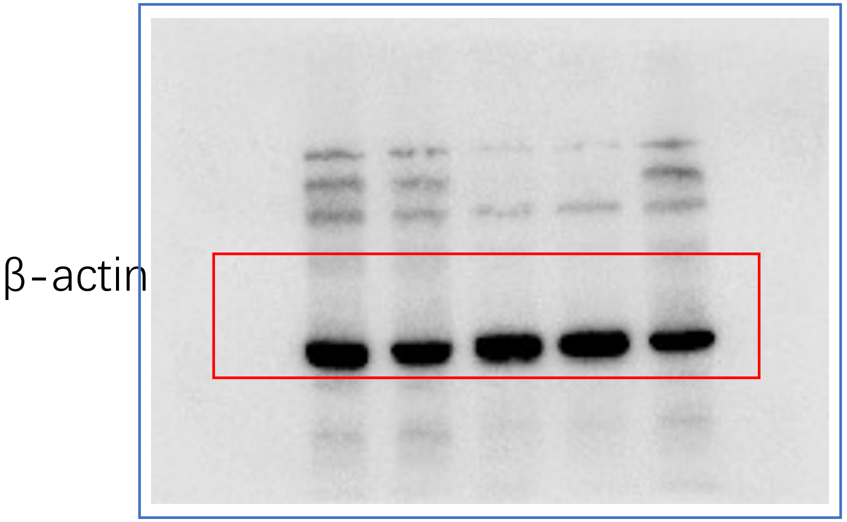

β-actin

Figure S4D

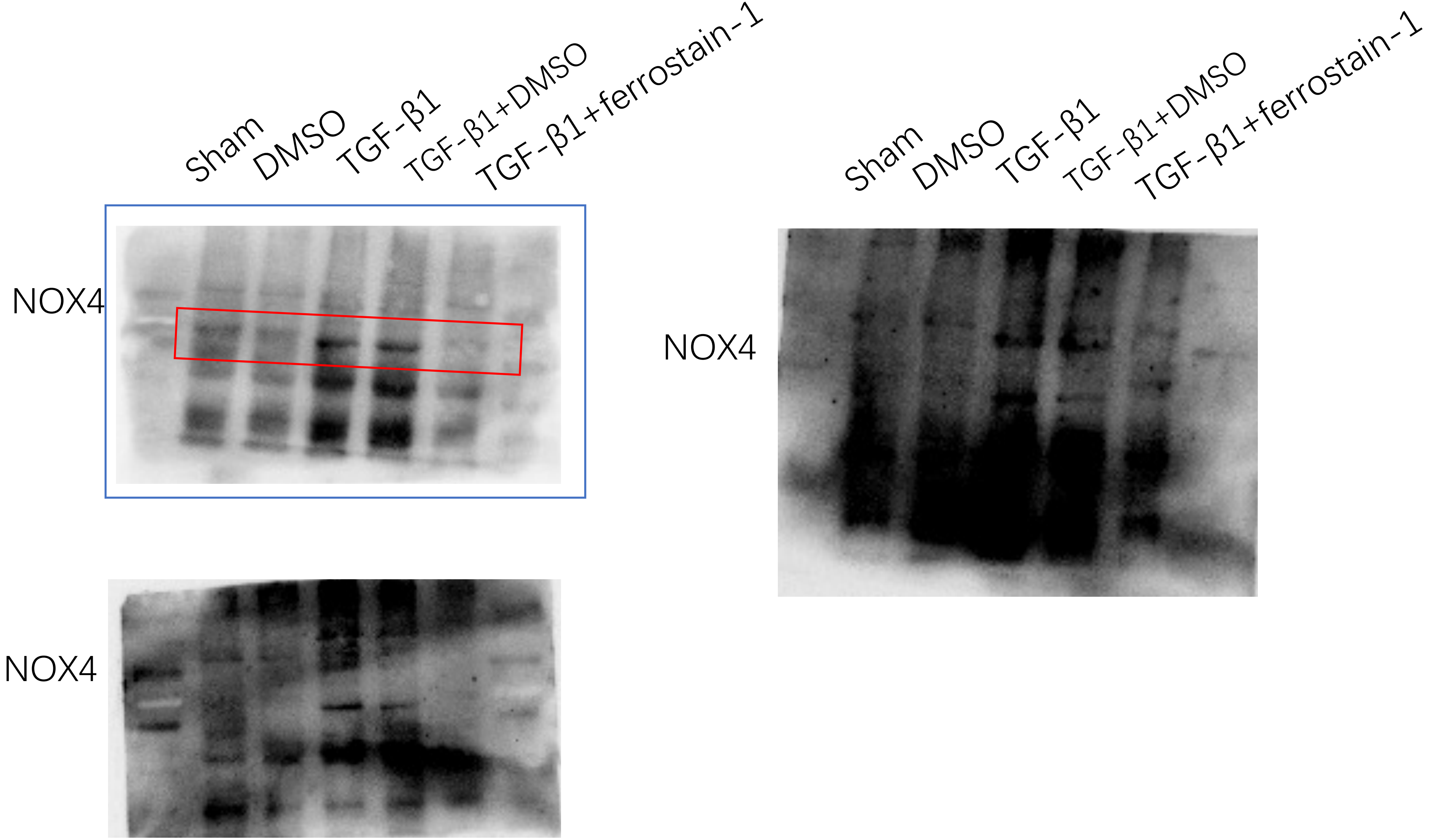
Sham
DMSO
TGF-β1
TGF-β1+DMSO
TGF-β1+ferrostain-1
NOX4
NOX4
Sham
DMSO
TGF-β1
TGF-β1+DMSO
TGF-β1+ferrostain-1
NOX4

Figure S4D

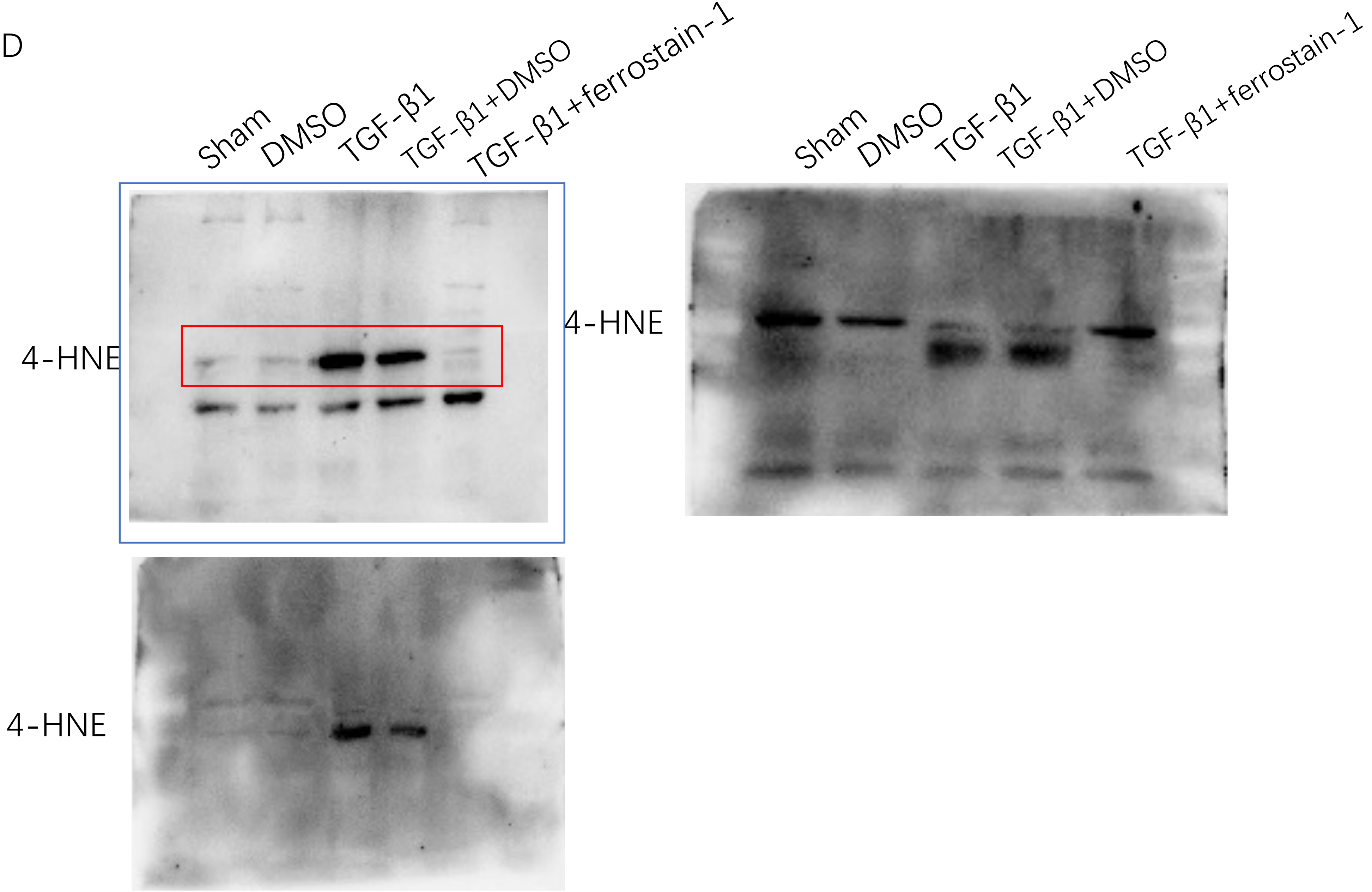

Sham
DMSO
TGF-β1
TGF-β1+DMSO
TGF-β1+ferrostain-1
4-HNE
4-HNE
Sham
DMSO
TGF-β1
TGF-β1+DMSO
TGF-β1+ferrostain-1
4-HNE

Figure S4D

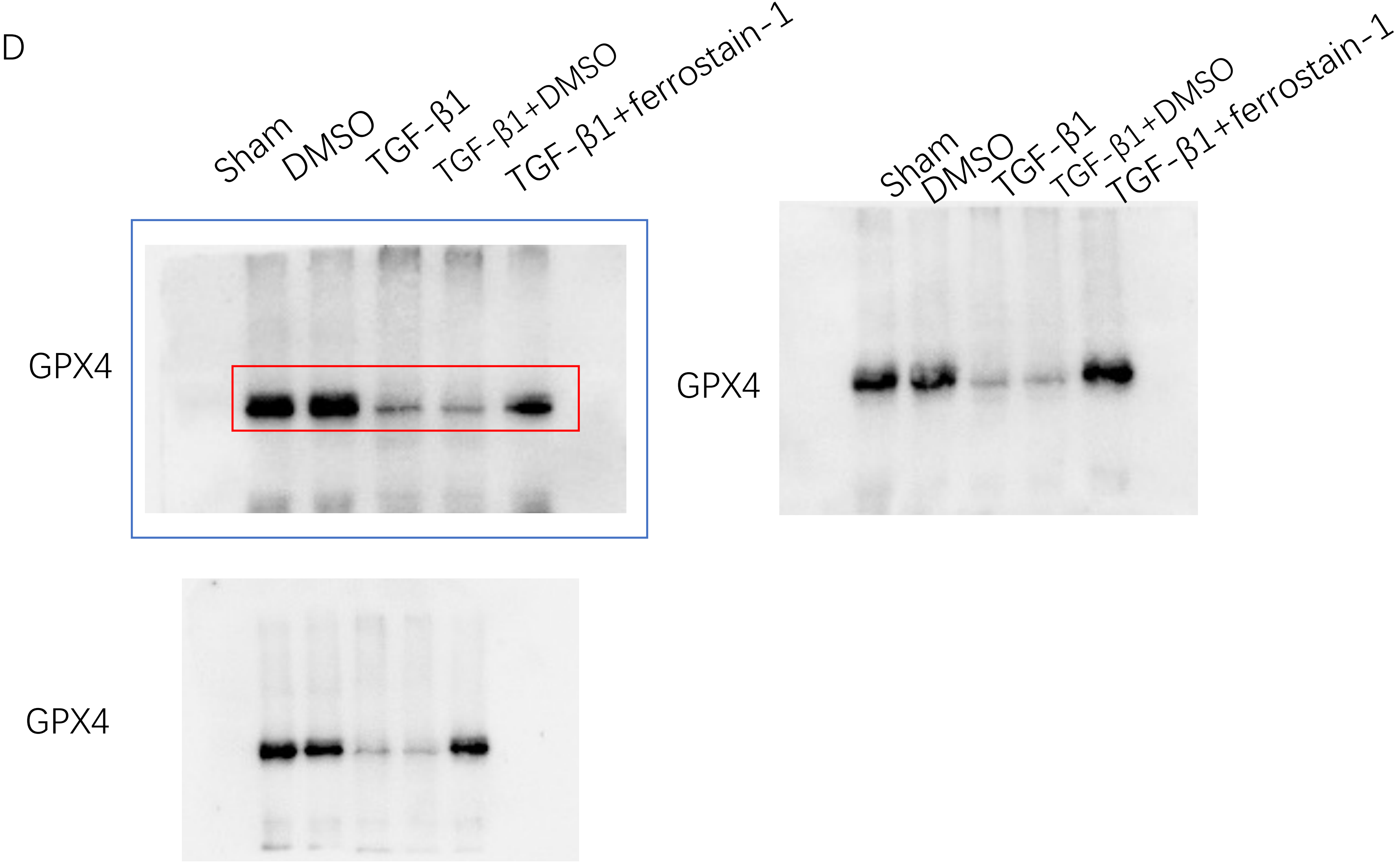
Sham
DMSO
TGF-β1
TGF-β1+DMSO
TGF-β1+ferrostain-1
GPX4
Sham
DMSO
TGF-β1
TGF-β1+DMSO
TGF-β1+ferrostain-1
GPX4
GPX4

Figure S4D

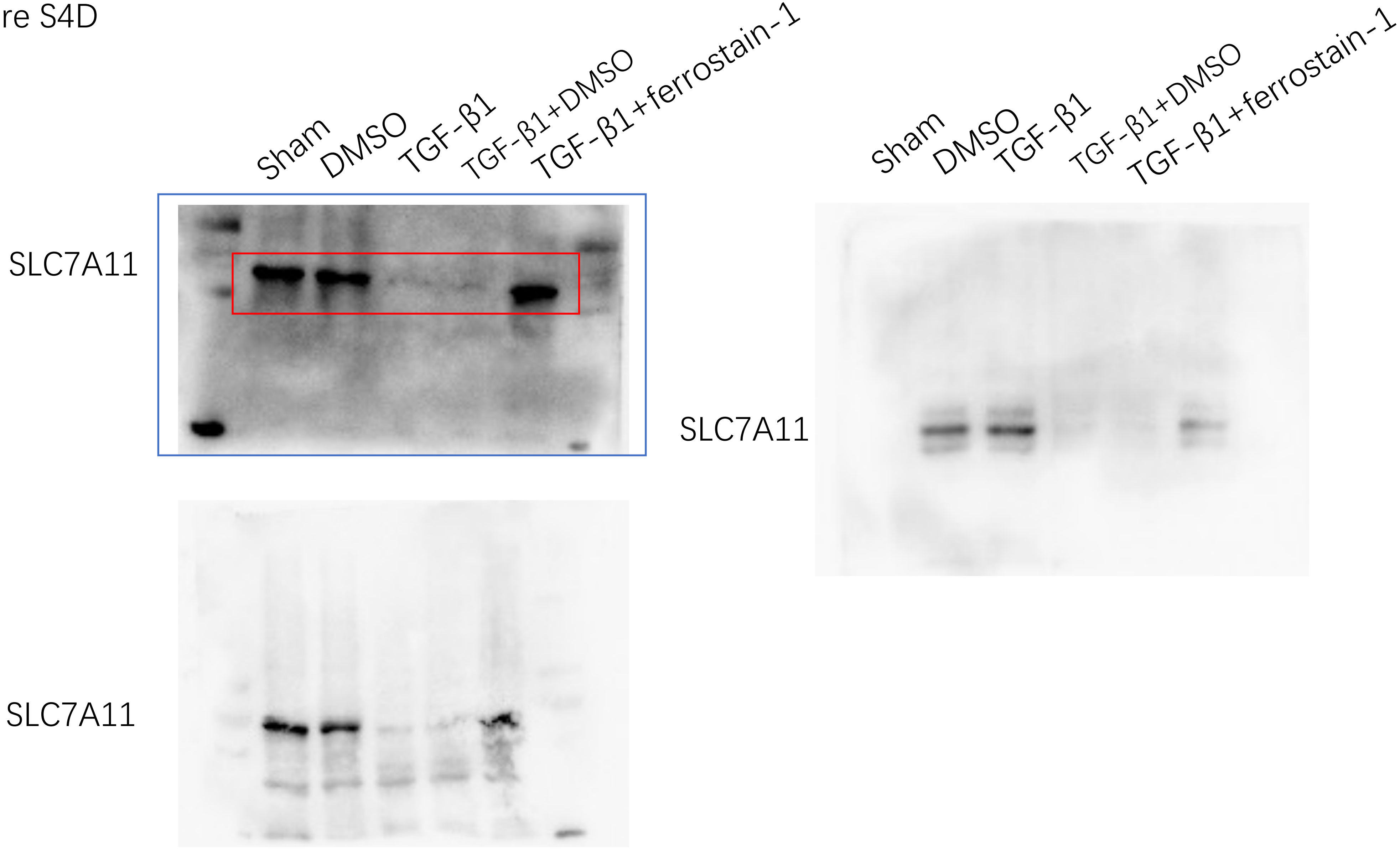
Sham
DMSO
TGF-β1
TGF-β1+DMSO
TGF-β1+ferrostain-1
SLC7A11
SLC7A11
Sham
DMSO
TGF-β1
TGF-β1+DMSO
TGF-β1+ferrostain-1
SLC7A11

Figure S4D

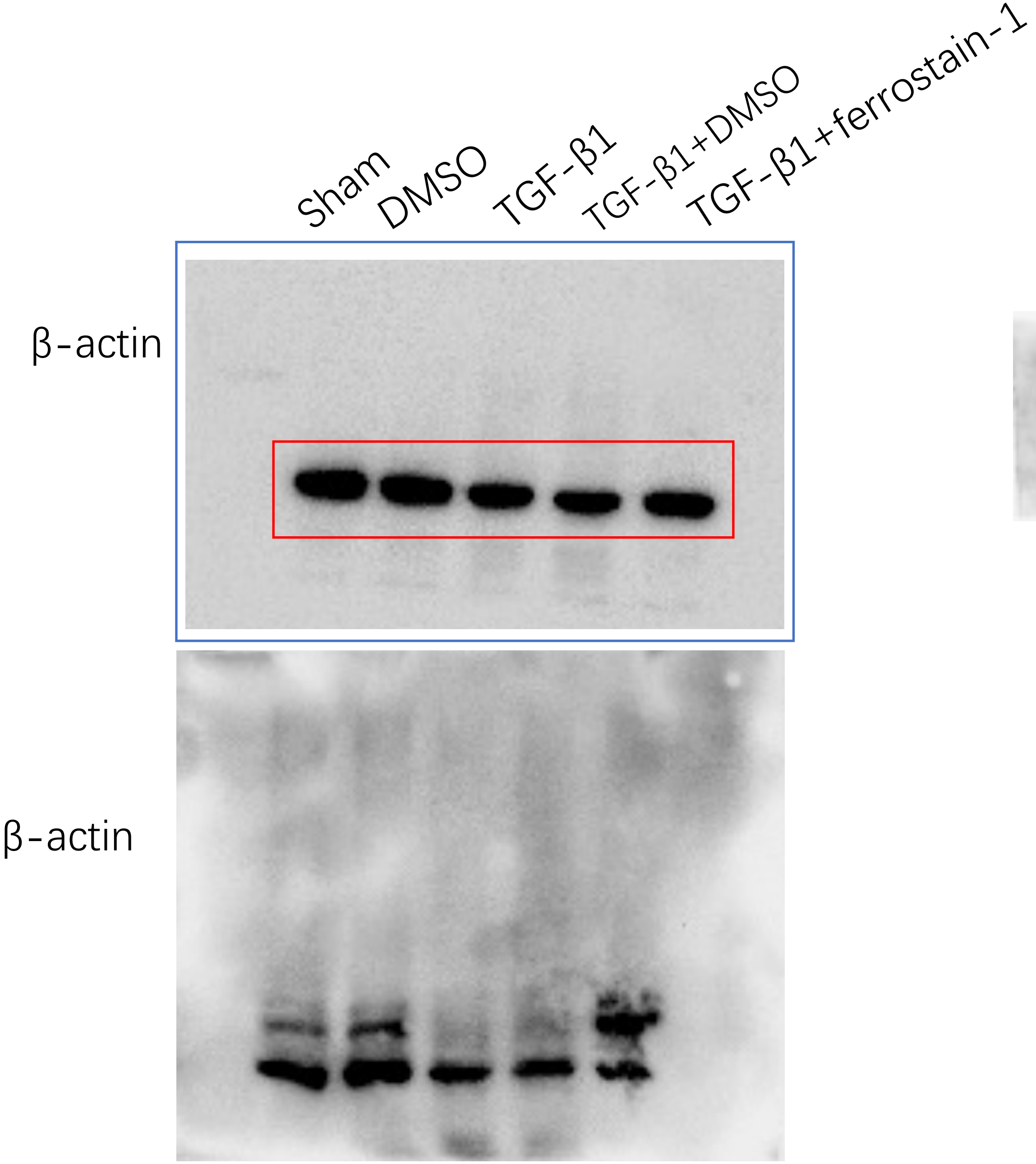
Sham
DMSO
TGF-β1
TGF-β1+DMSO
TGF-β1+ferrostain-1
β-actin
β-actin

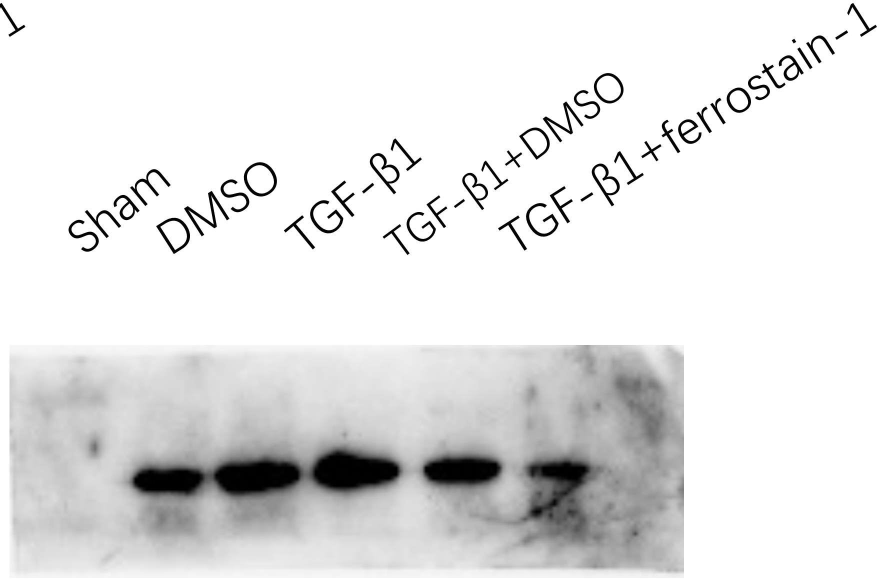
Sham
DMSO
TGF-β1
TGF-β1+DMSO
TGF-β1+ferrostain-1

Figure S4E

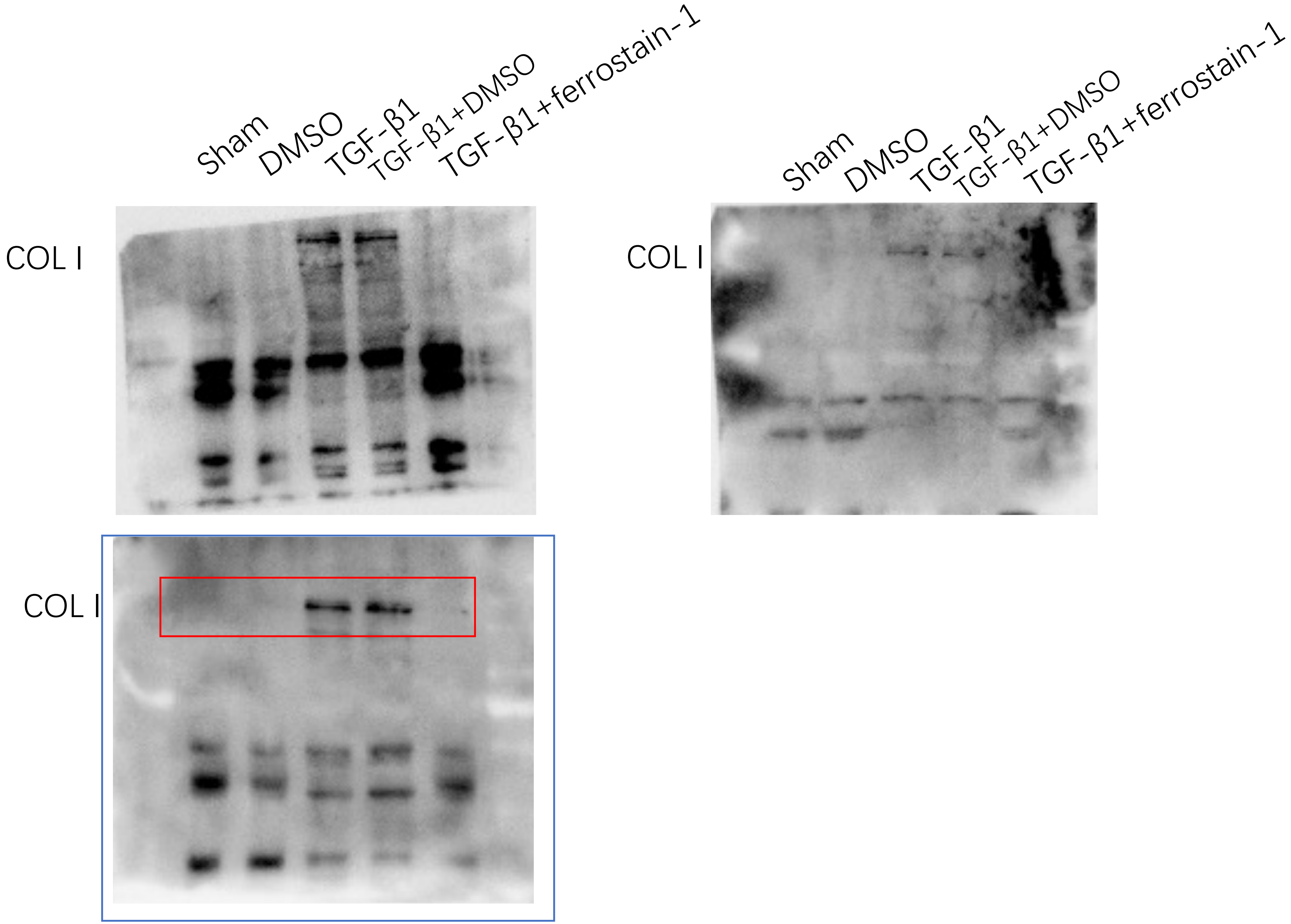
Sham
DMSO
TGF-β1
TGF-β1+DMSO
TGF-β1+ferrostain-1
COL I
Sham
DMSO
TGF-β1
TGF-β1+DMSO
TGF-β1+ferrostain-1
COL I
COL I

Figure S4E

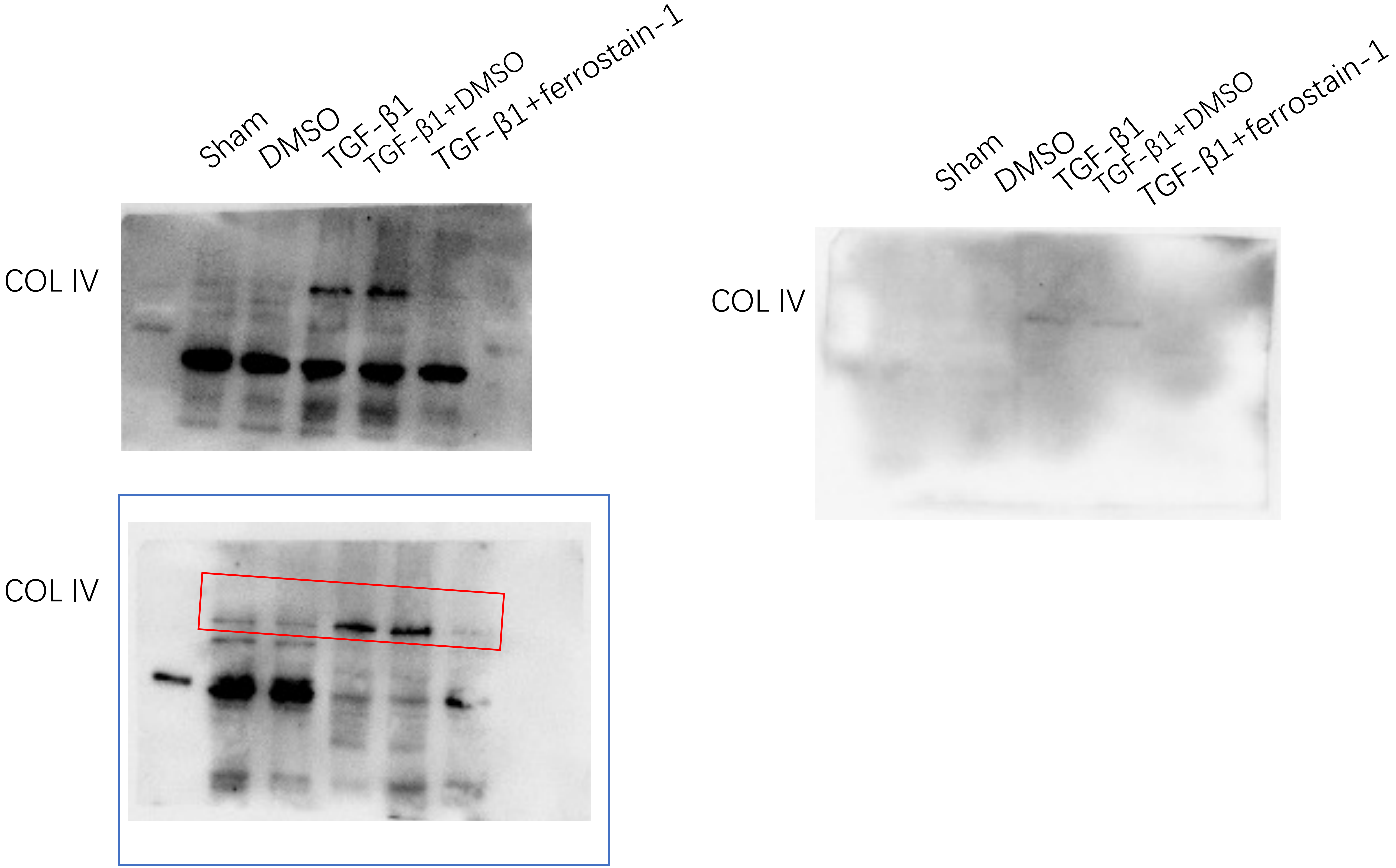
Sham
DMSO
TGF-β1
TGF-β1+DMSO
TGF-β1+ferrostain-1
COL IV
COL IV
Sham
DMSO
TGF-β1
TGF-β1+DMSO
TGF-β1+ferrostain-1
COL IV

Figure S4E

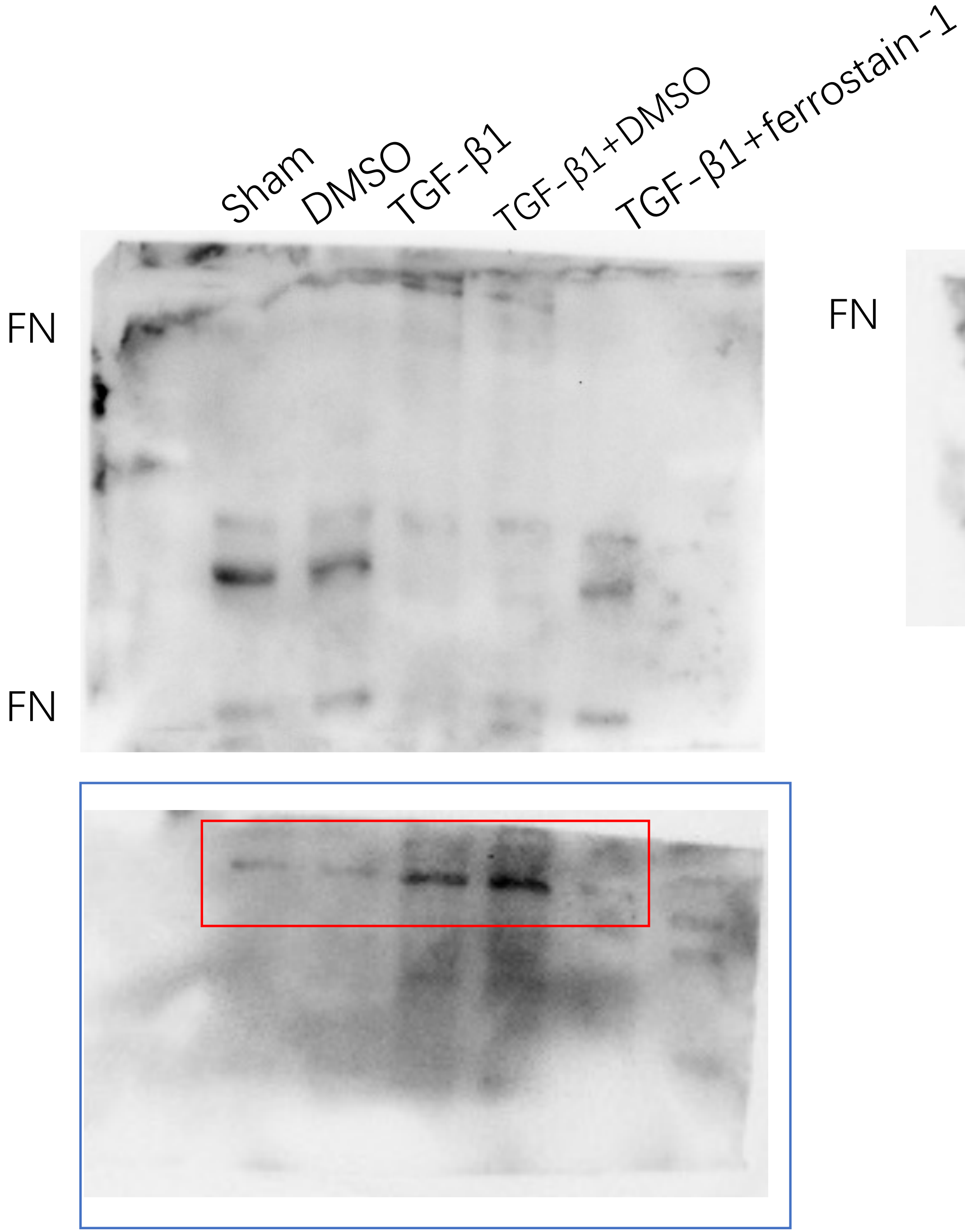

Sham
DMSO
TGF-β1
TGF-β1+DMSO
TGF-β1+ferrostain-1
FN
FN

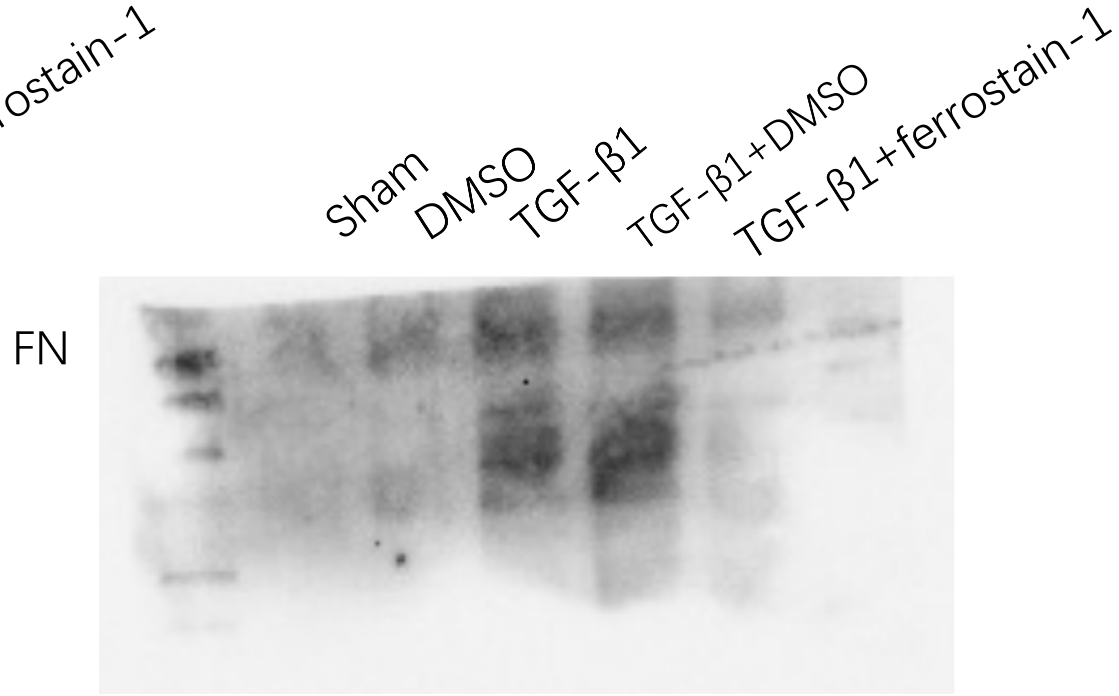

Sham
DMSO
TGF-β1
TGF-β1+DMSO
TGF-β1+ferrostain-1
FN

Figure S4E

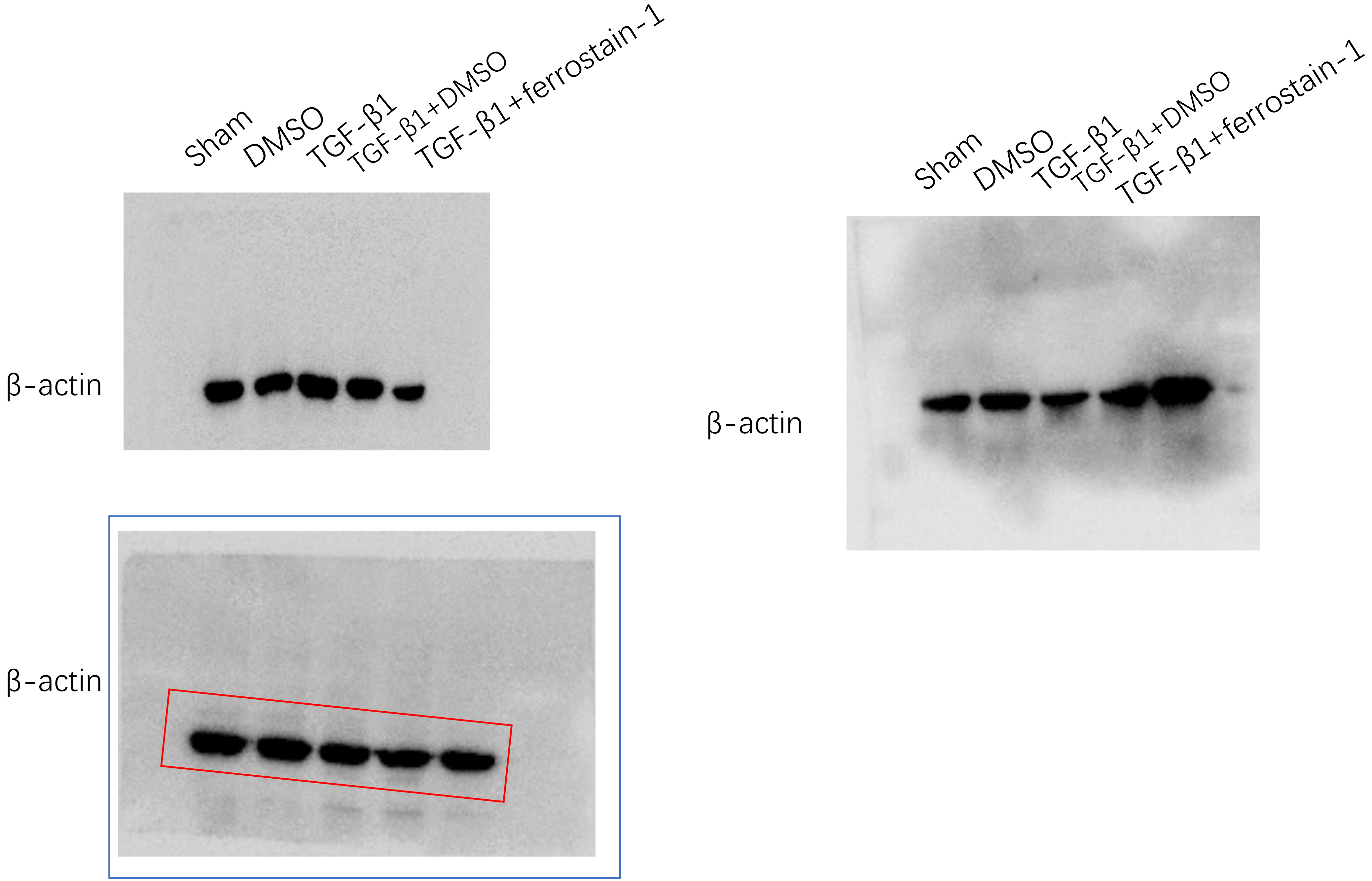
Sham
DMSO
TGF-β1
TGF-β1+DMSO
TGF-β1+ferrostain-1
β-actin
β-actin
Sham
DMSO
TGF-β1
TGF-β1+DMSO
TGF-β1+ferrostain-1
β-actin

Figure S4E

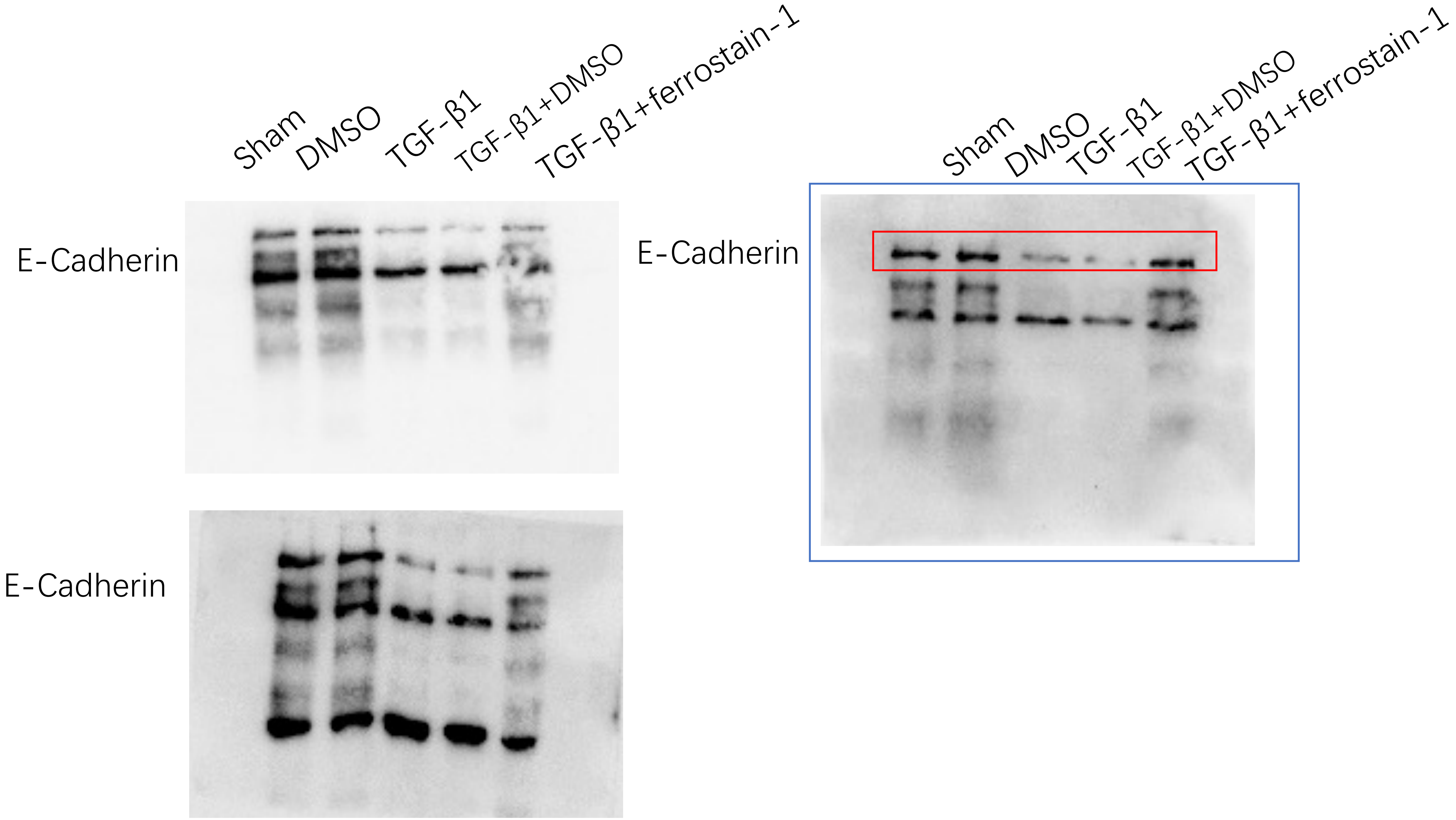
Sham
DMSO
TGF-β1
TGF-β1+DMSO
TGF-β1+ferrostain-1
E-Cadherin
E-Cadherin
Sham
DMSO
TGF-β1
TGF-β1+DMSO
TGF-β1+ferrostain-1
E-Cadherin

Figure S4E

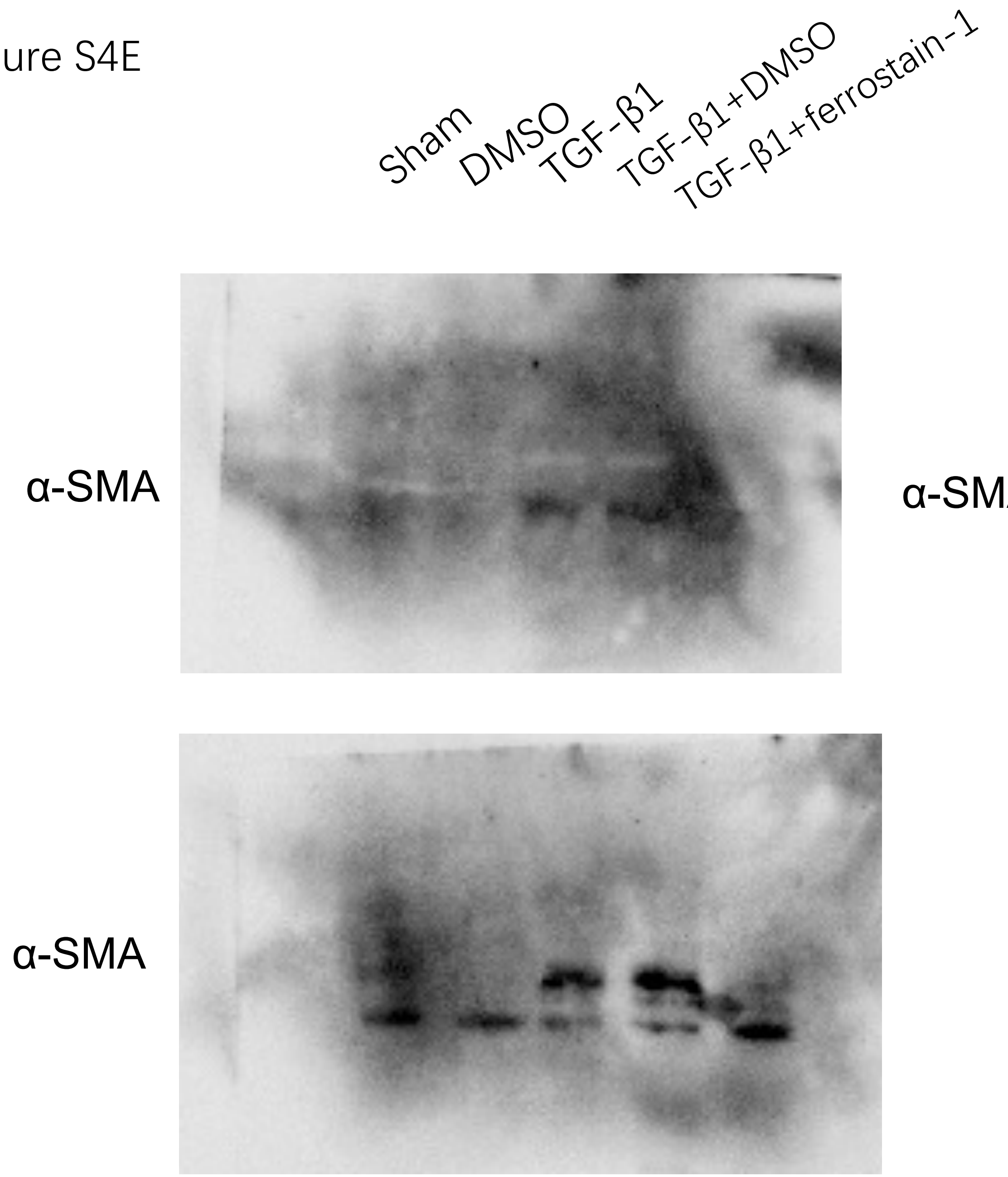
Sham
DMSO
TGF-β1
TGF-β1+DMSO
TGF-β1+ferrostain-1
α-SMA
α-SMA

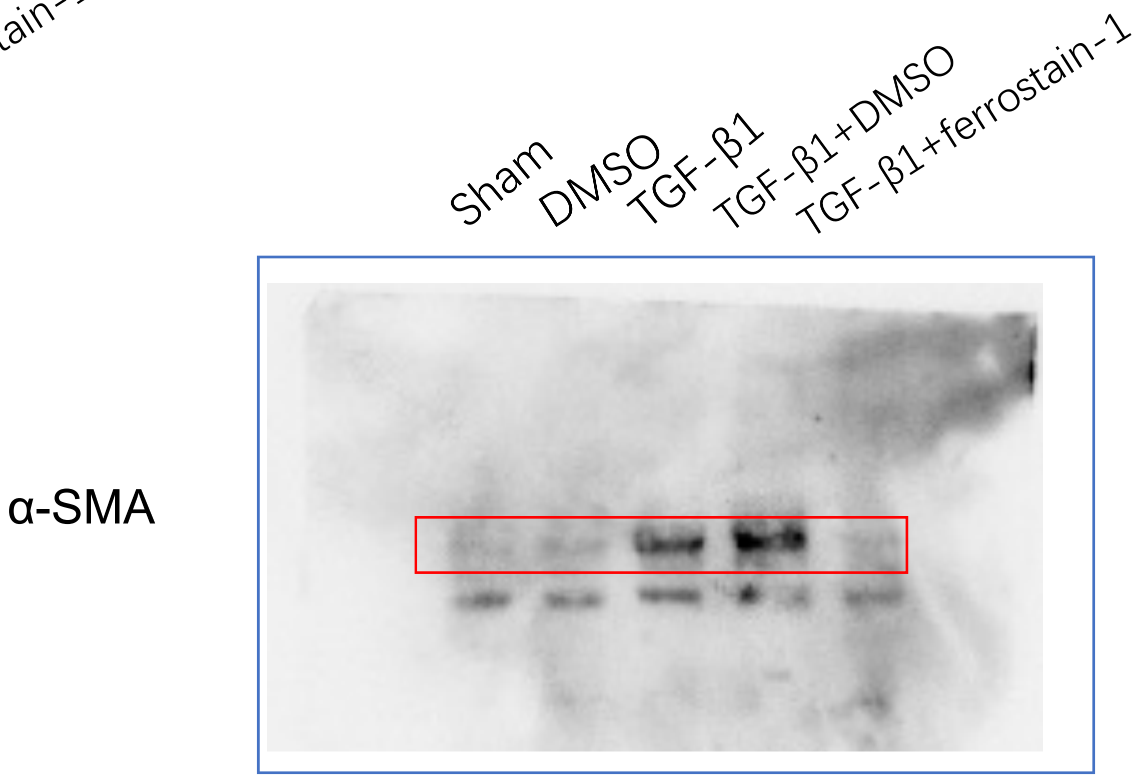
Sham
DMSO
TGF-β1
TGF-β1+DMSO
TGF-β1+ferrostain-1
α-SMA

Figure S4E

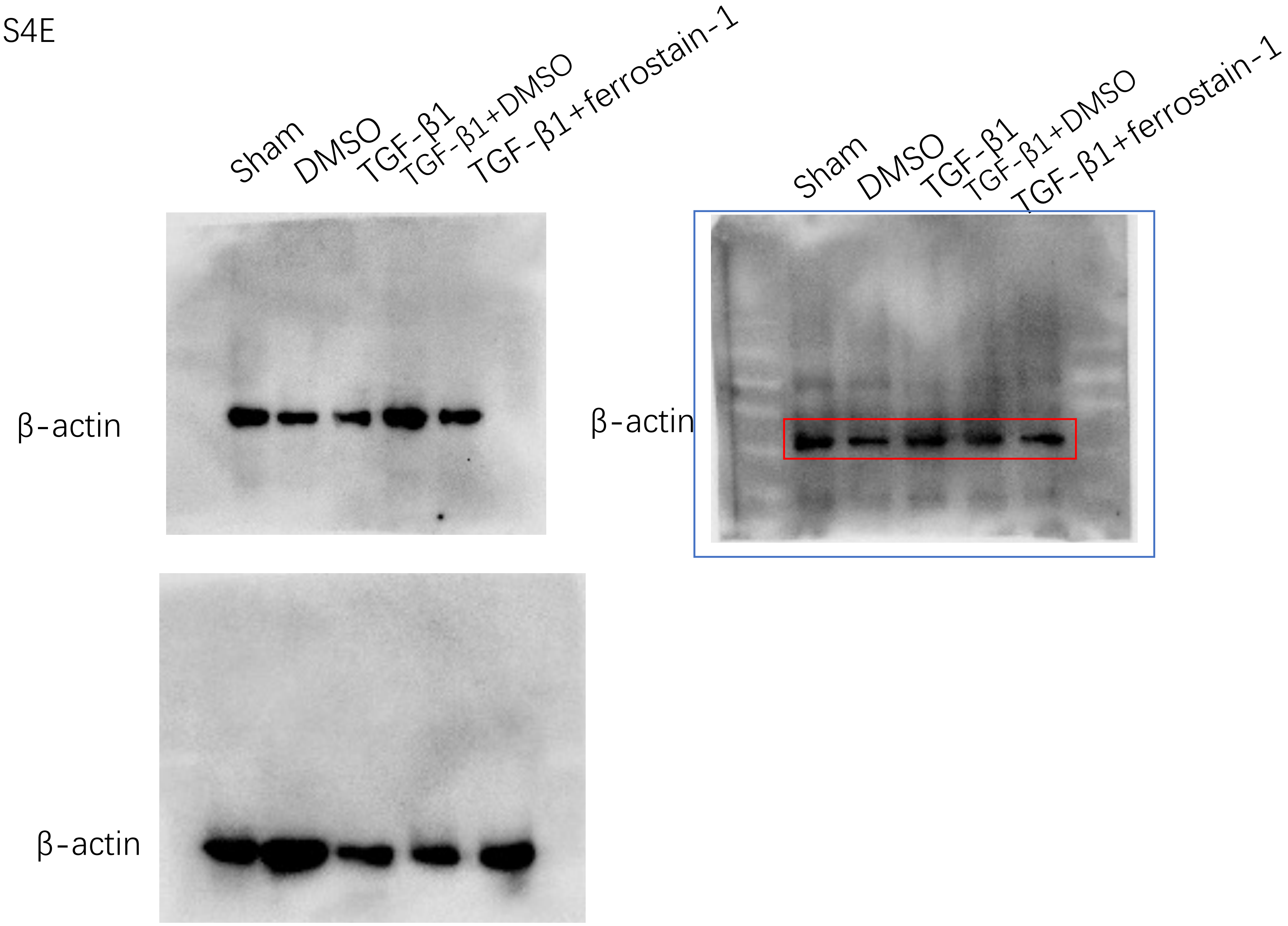
Sham
DMSO
TGF-β1
TGF-β1+DMSO
TGF-β1+ferrostain-1
β-actin
β-actin
Sham
DMSO
TGF-β1
TGF-β1+DMSO
TGF-β1+ferrostain-1
β-actin

Figure 8C

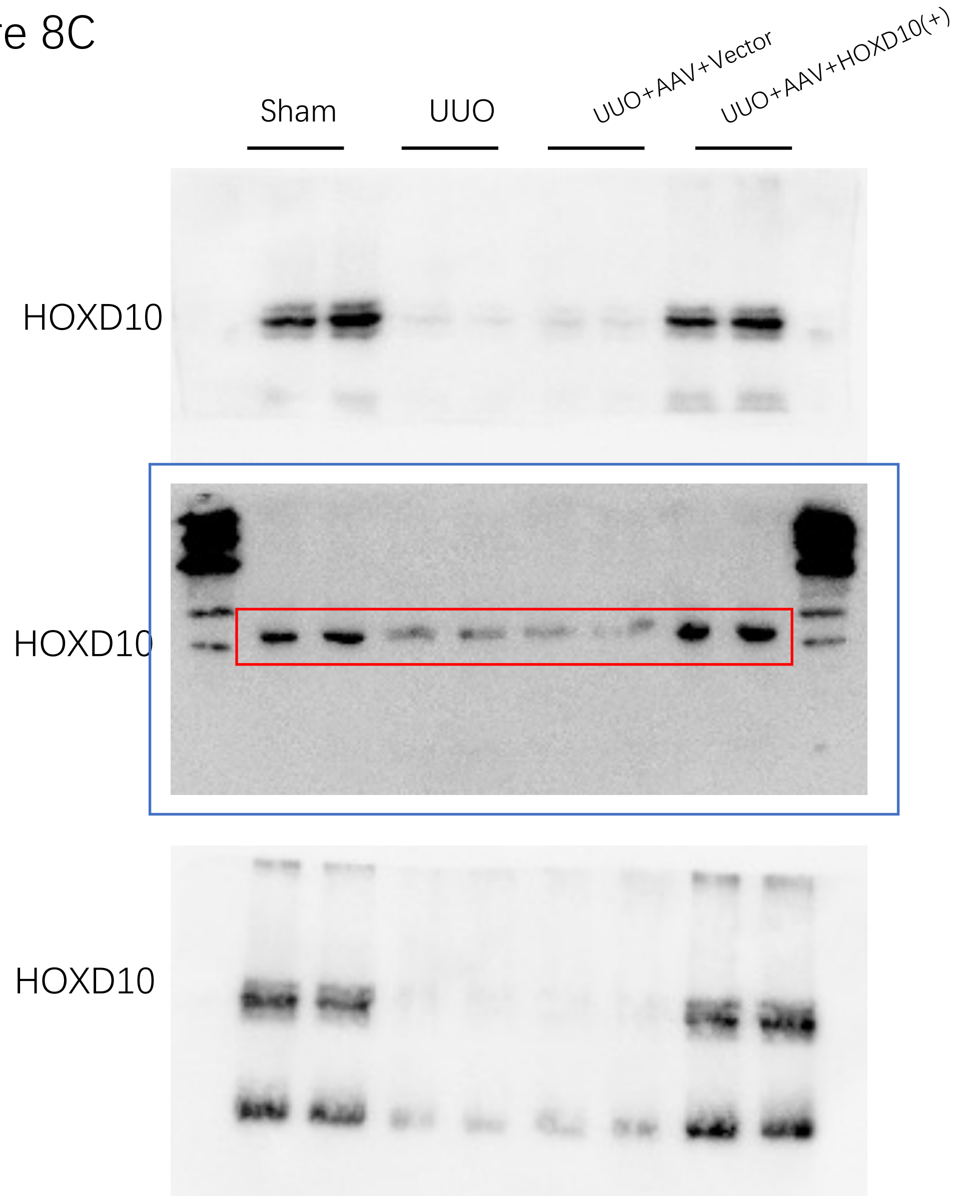

Figure 8C

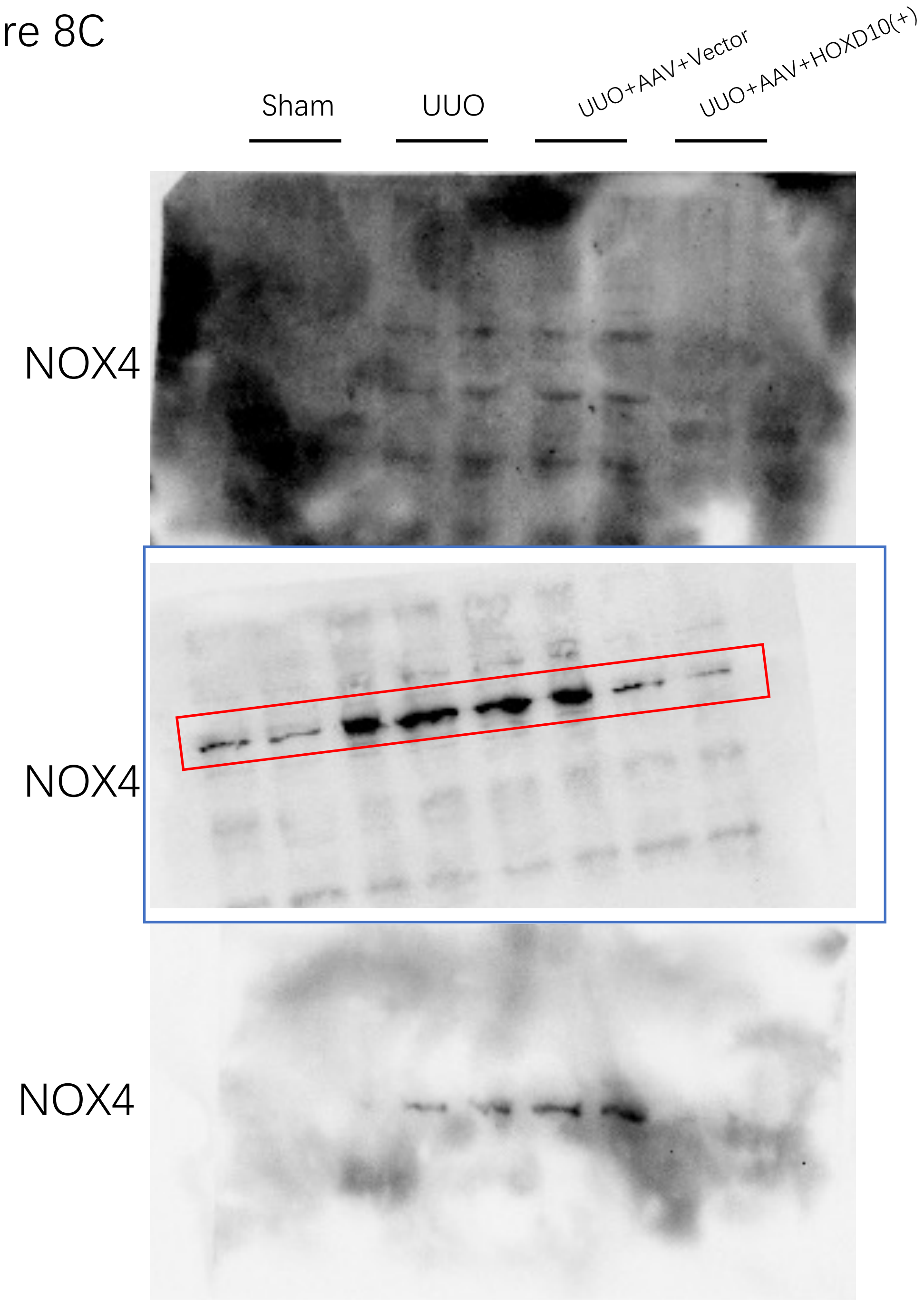

Figure 8C

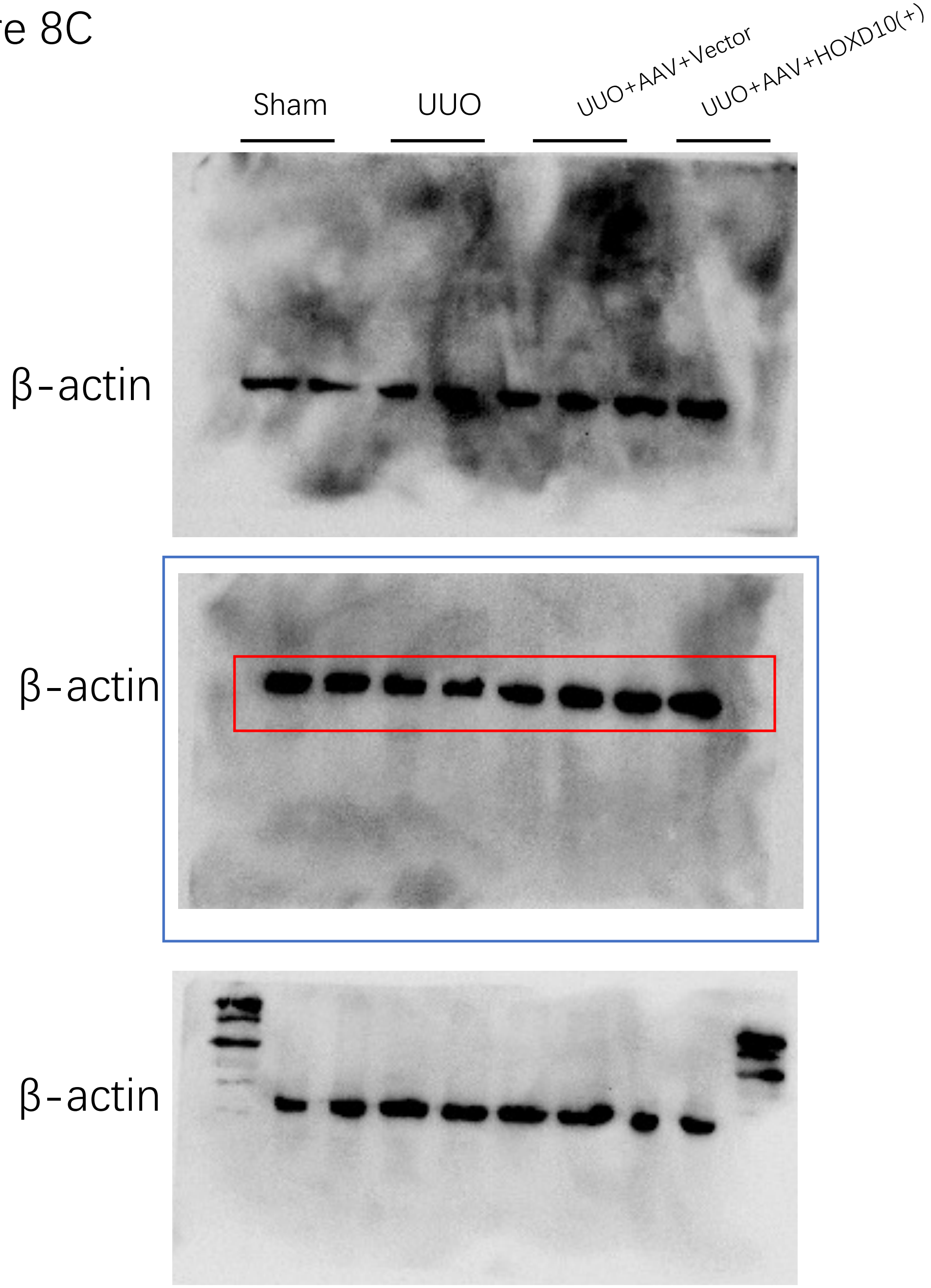

Figure 10C

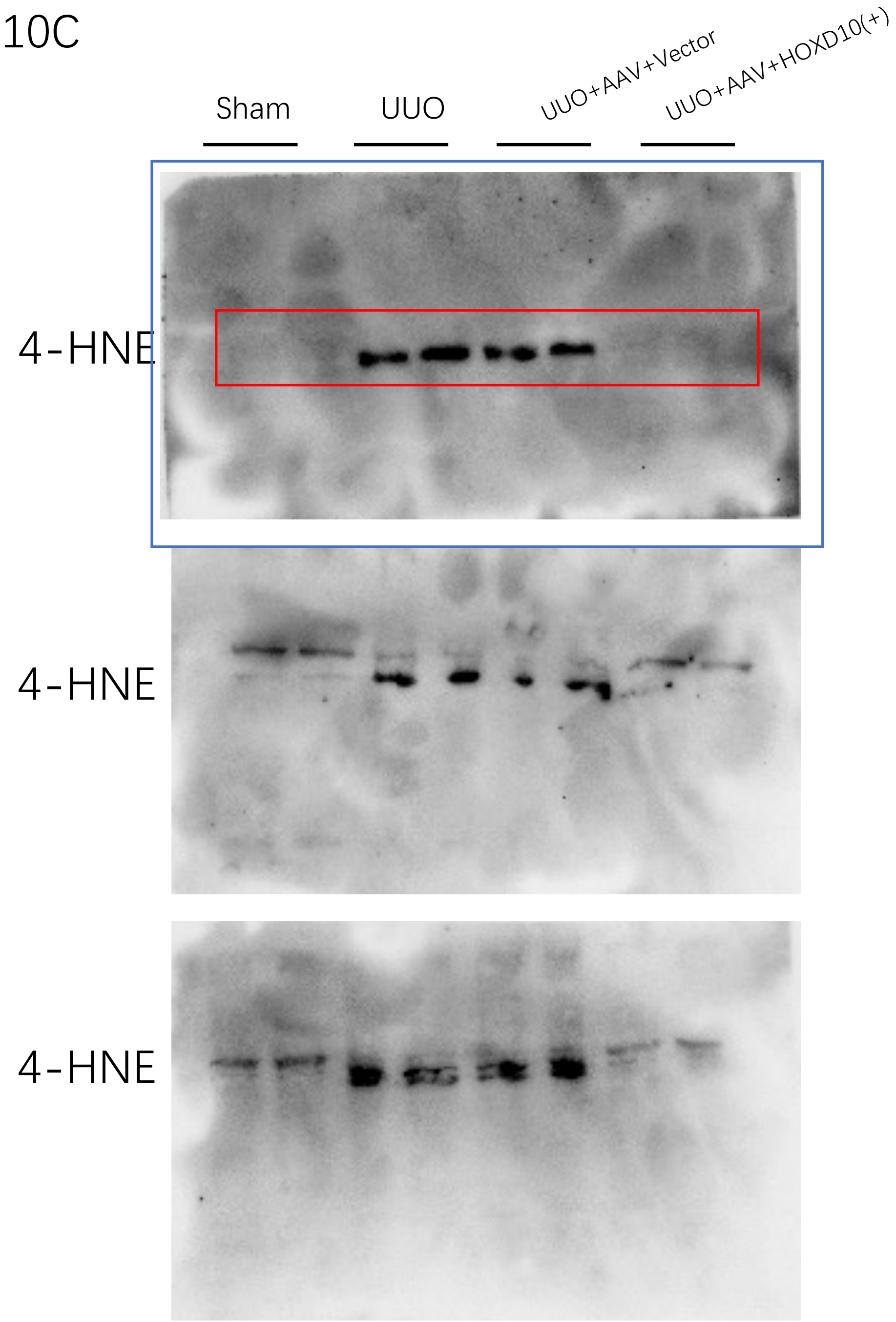

Figure 10C

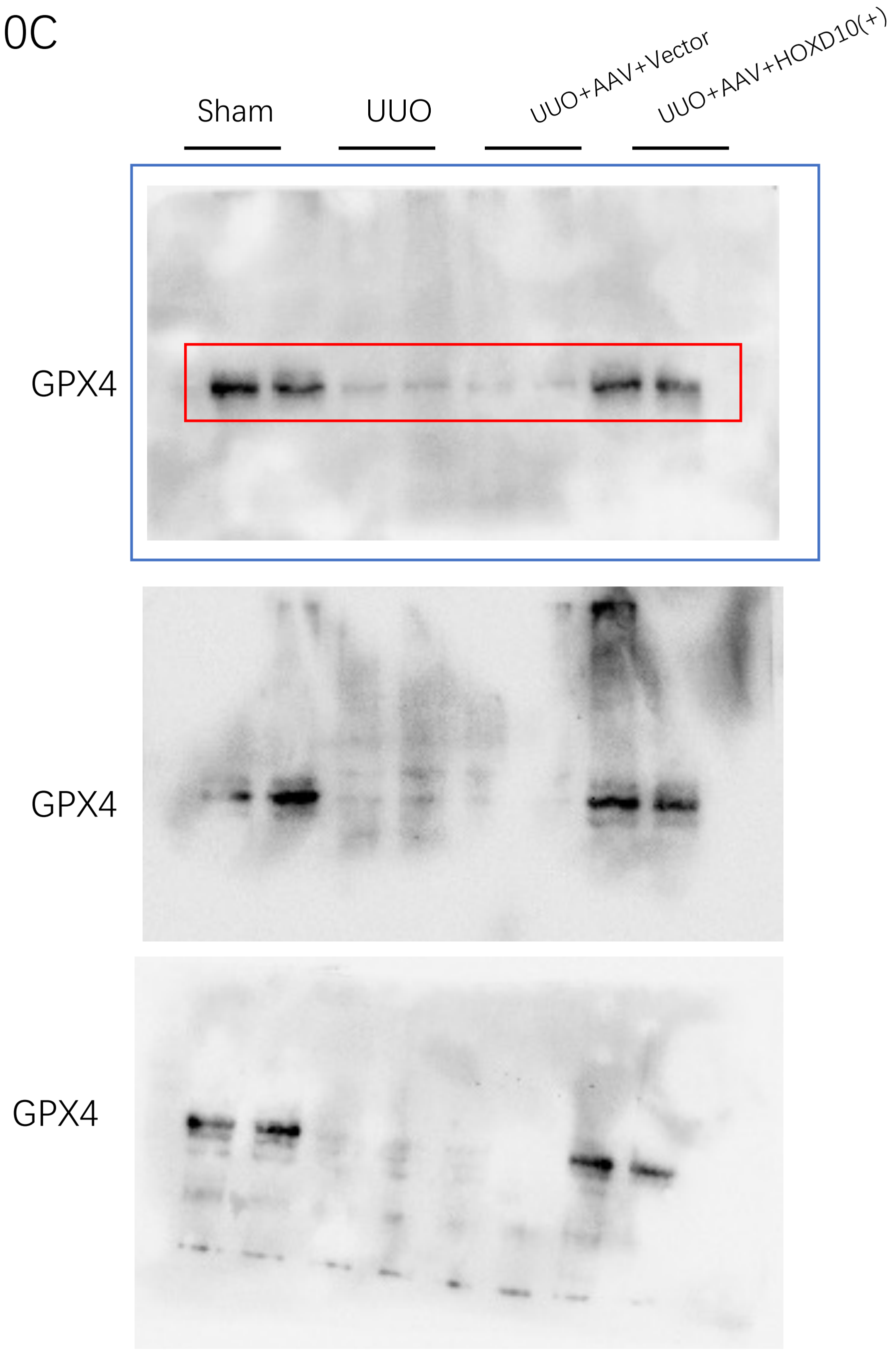

Figure 10C

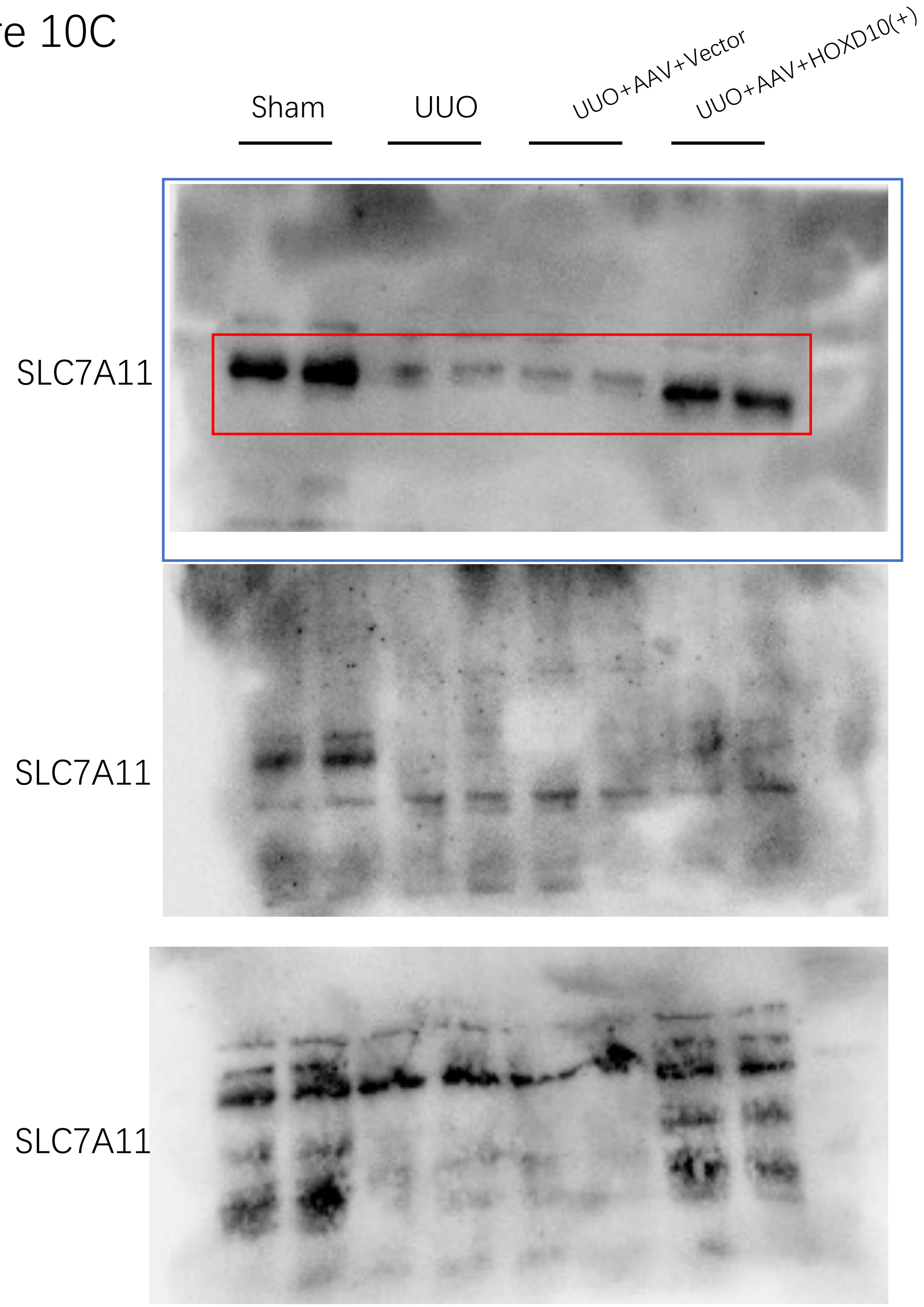
Sham
UUO
UUO+AAV+Vector
UUO+AAV+HOXD10(+)
SLC7A11
SLC7A11
SLC7A11

Figure 10C

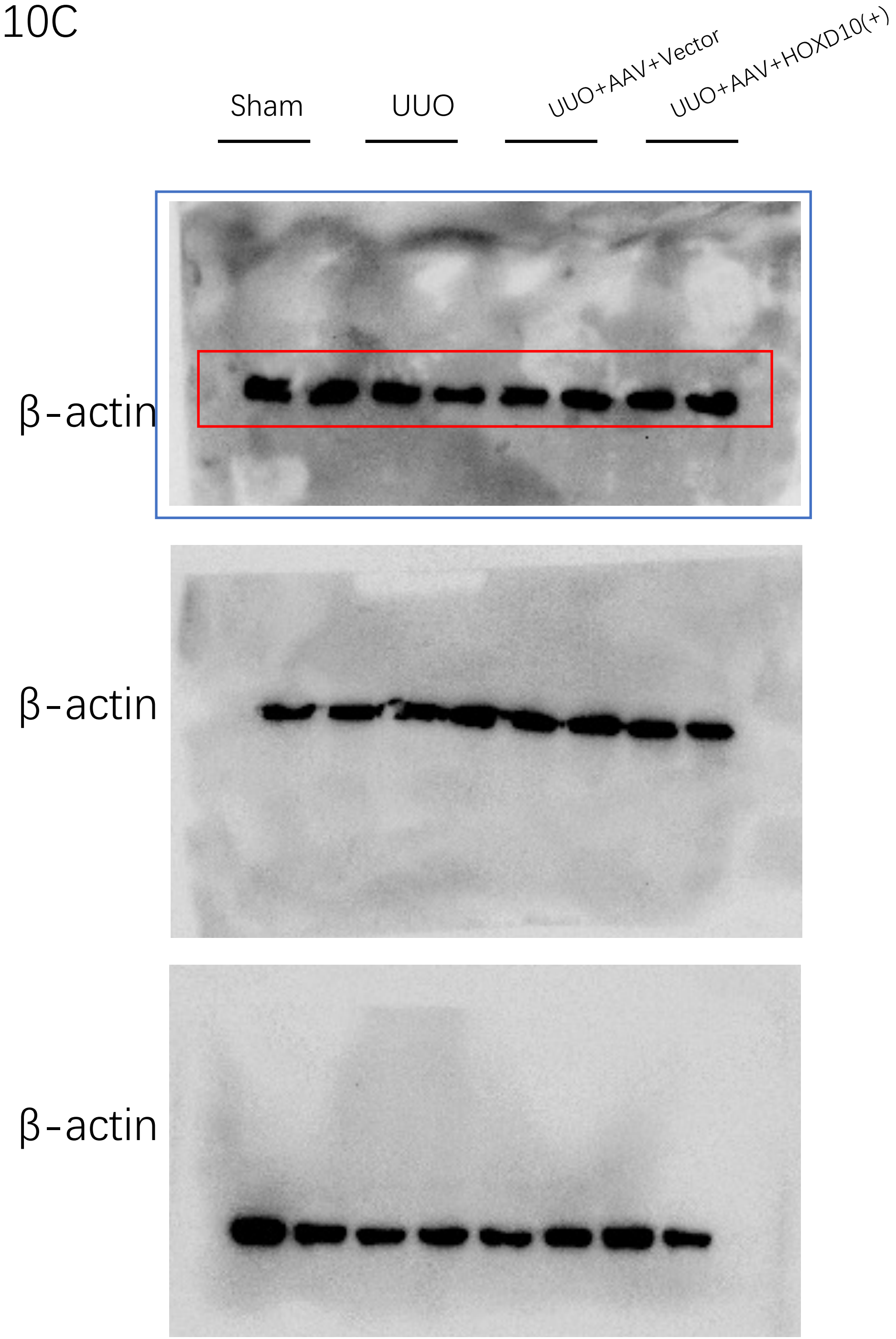

Figure 10C

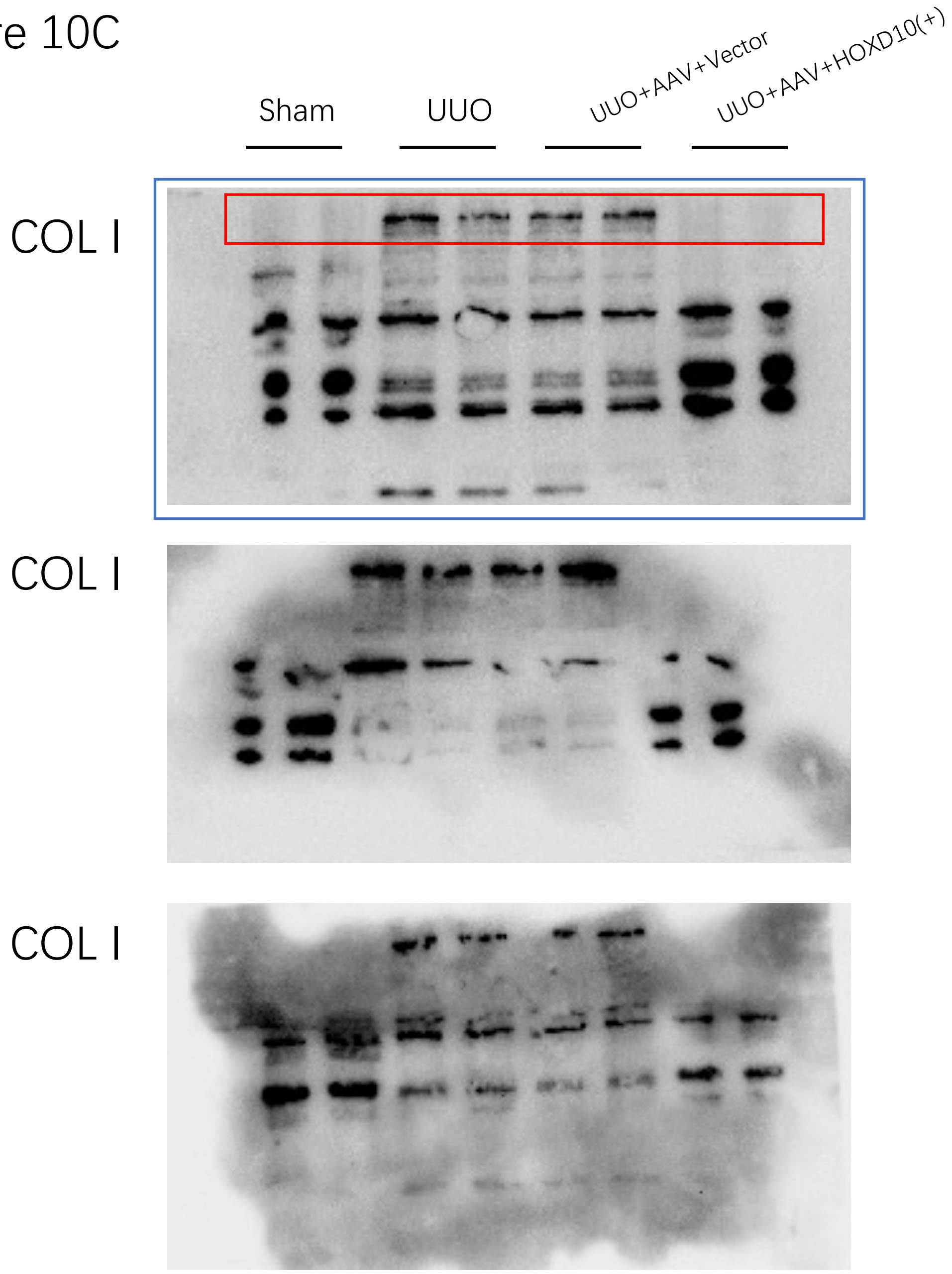

Figure 10C

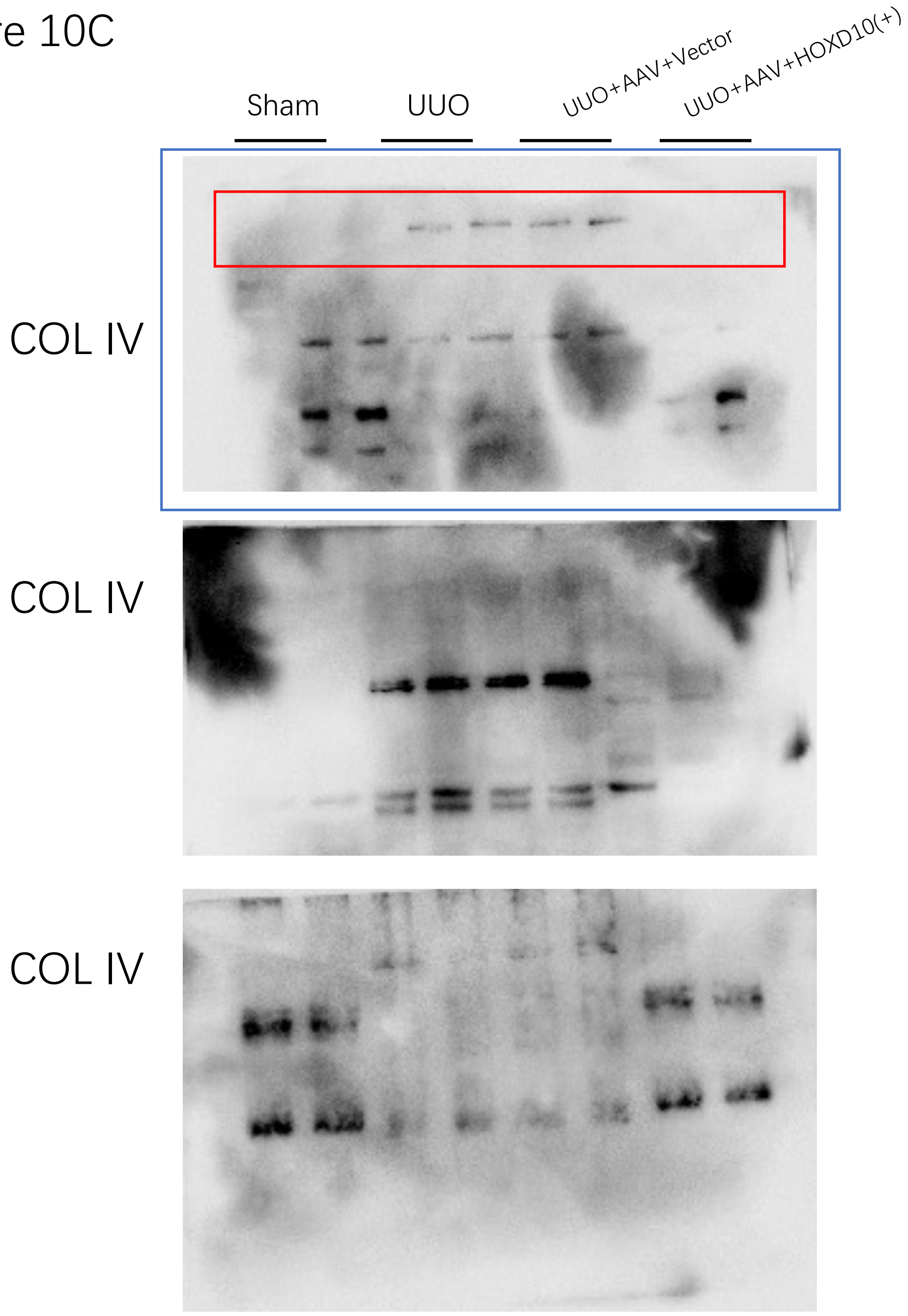

Figure 10C

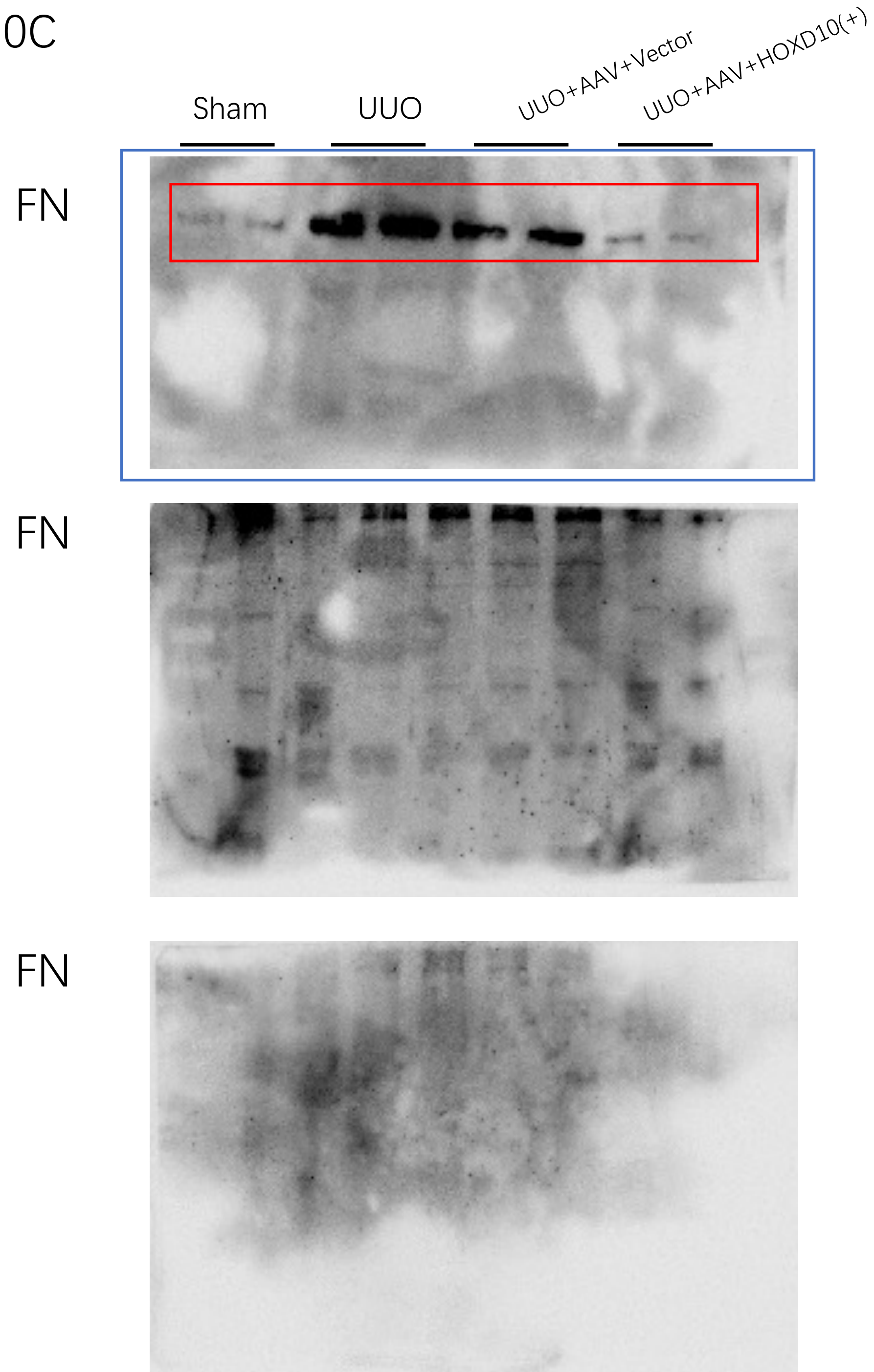

Figure 10C

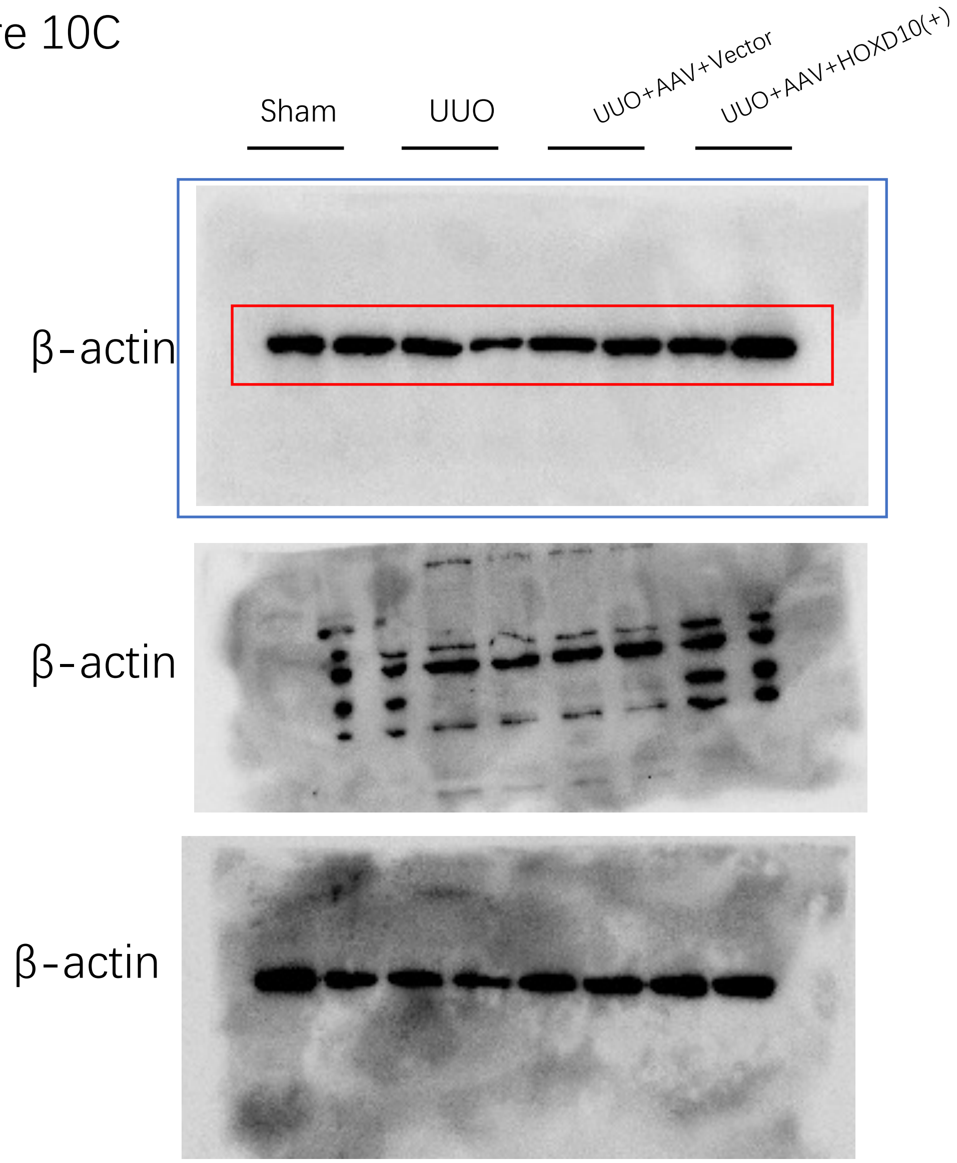

Figure 10C

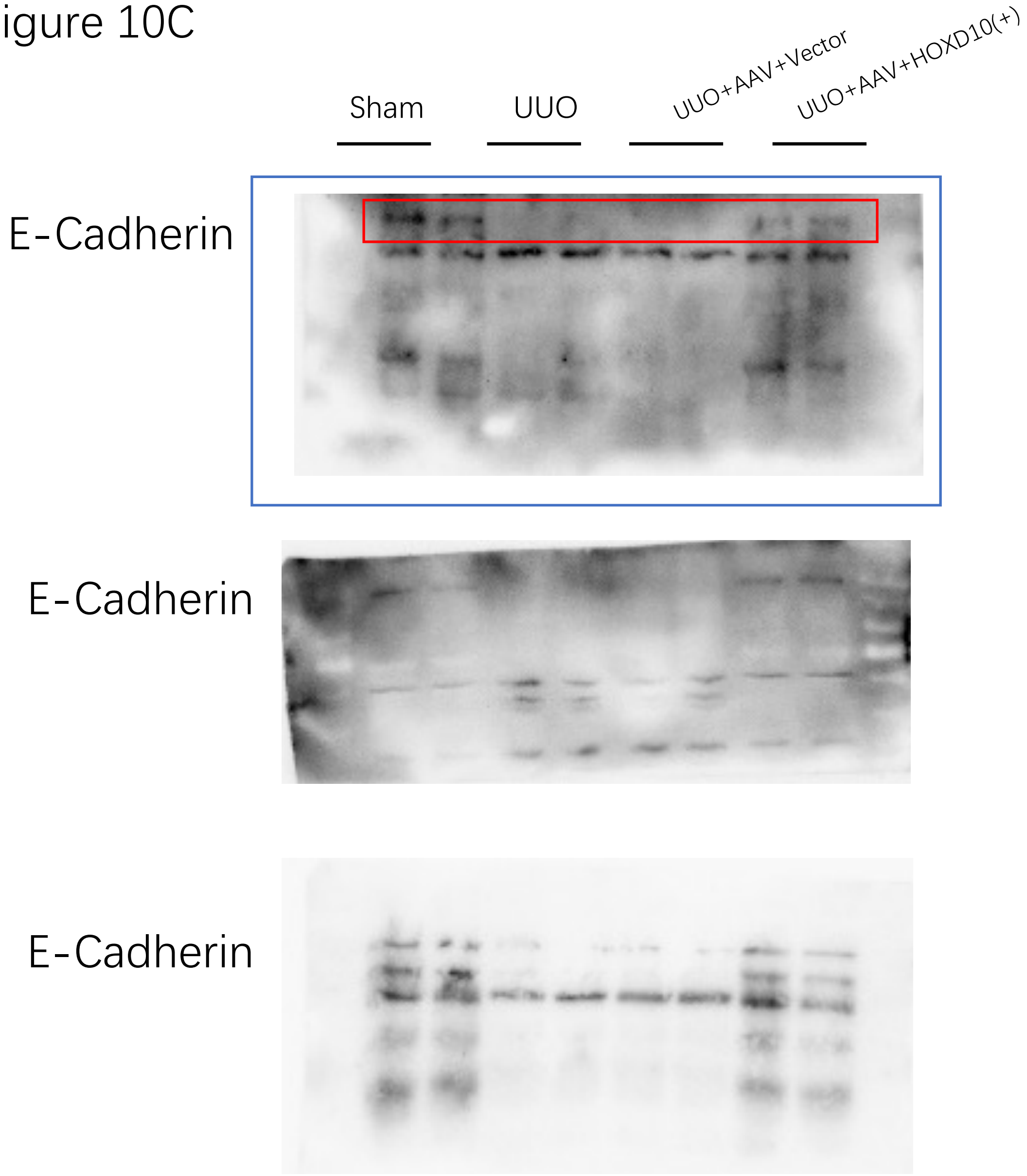
Sham
UUO
UUO+AAV+Vector
UUO+AAV+HOXD10(+)
E-Cadherin
E-Cadherin
E-Cadherin

Figure 10C

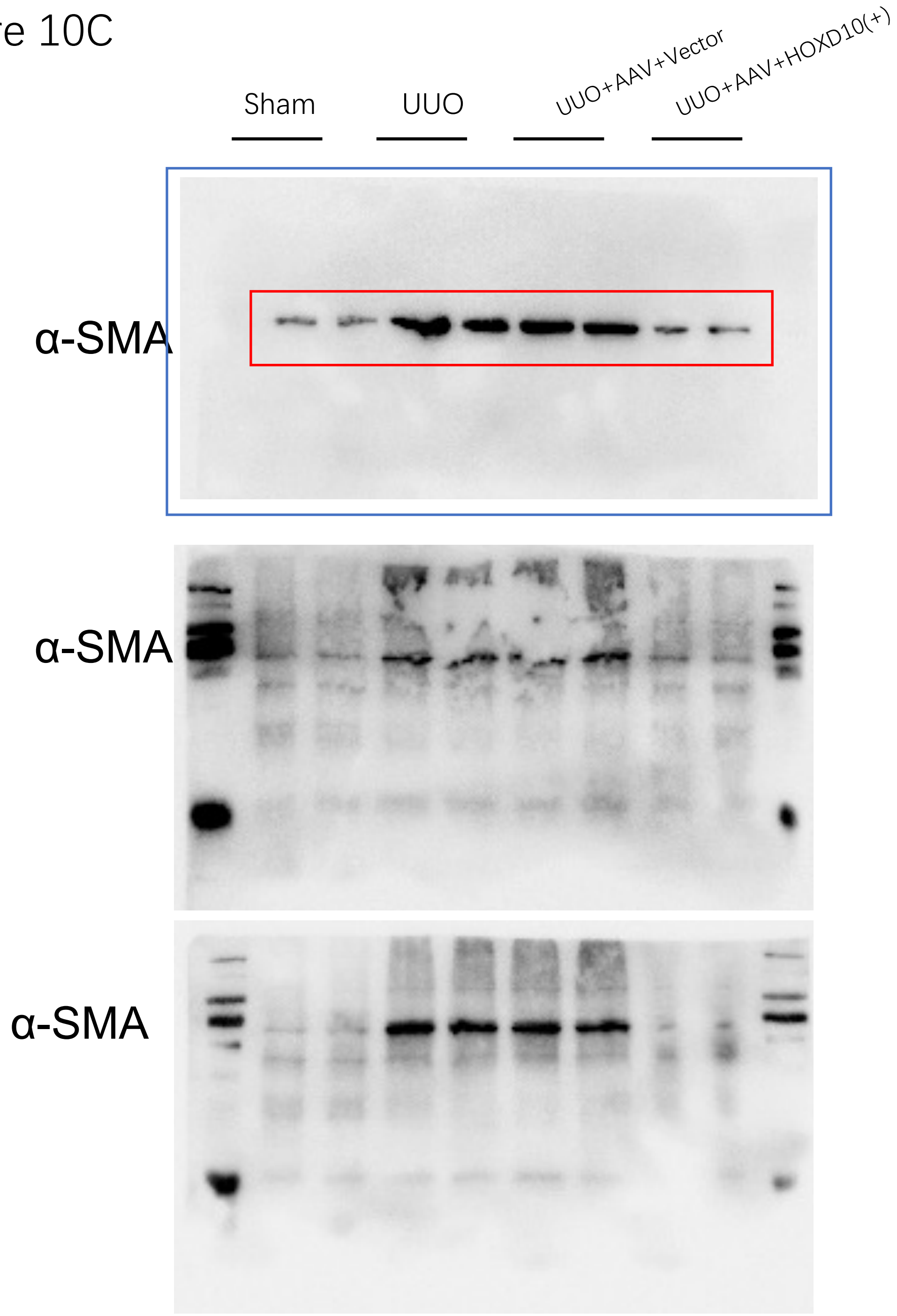

Figure 10C

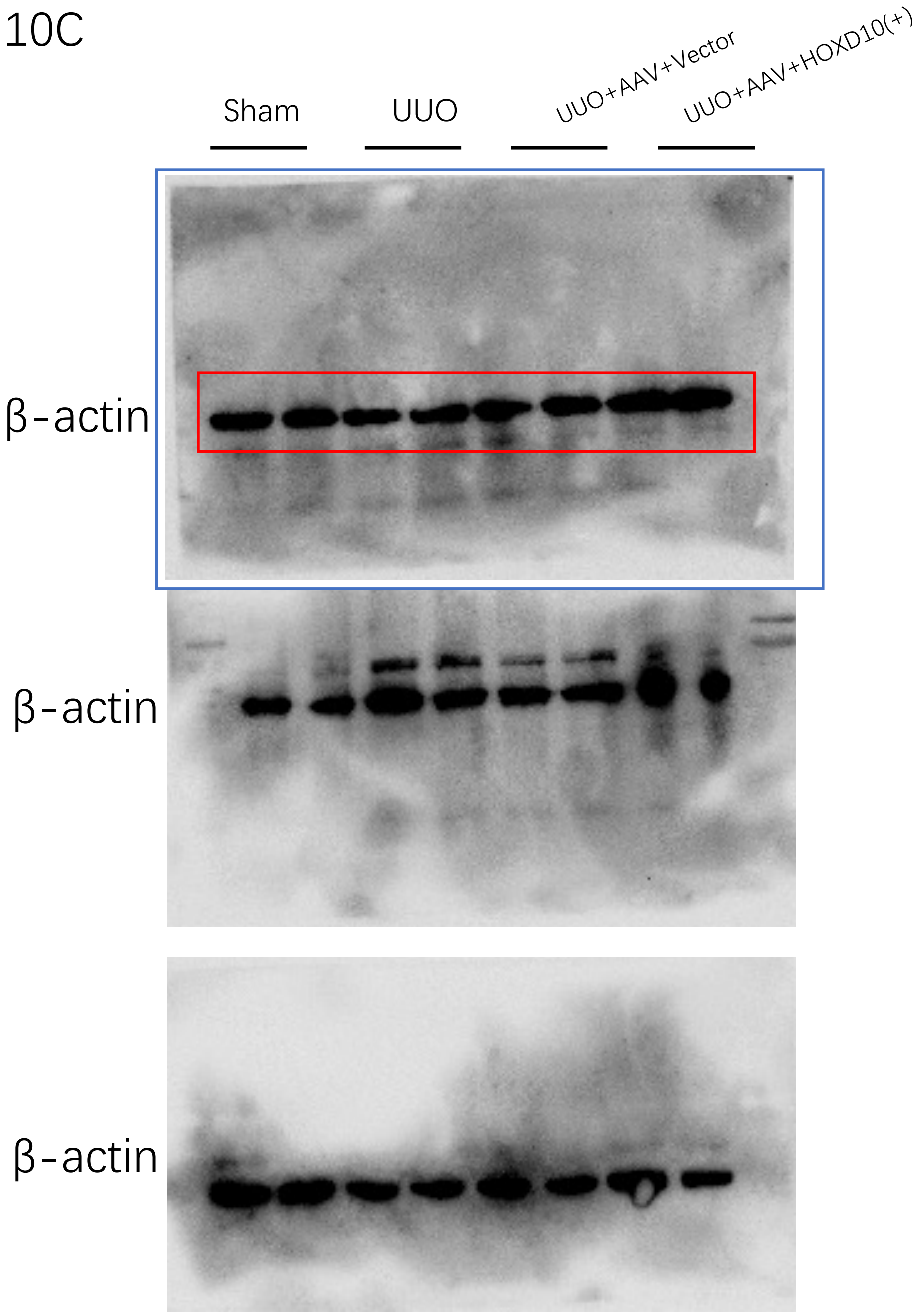

Supplement: Supplementary file 2 — WB GEL1 [file 41419_2024_6780_MOESM2_ESM.pdf]
